# The integrated analysis of RNA-seq and microRNA-seq depicts miRNA-mRNA networks involved in Japanese flounder (*Paralichthys olivaceus*) albinism

**DOI:** 10.1371/journal.pone.0181761

**Published:** 2017-08-04

**Authors:** Na Wang, Ruoqing Wang, Renkai Wang, Yongsheng Tian, Changwei Shao, Xiaodong Jia, Songlin Chen

**Affiliations:** 1 Key Laboratory for Sustainable Development of Marine Fisheries, Ministry of Agriculture, Yellow Sea Fisheries Research Institute, Chinese Academy of Fishery Sciences, Qingdao, China; 2 Laboratory for Marine Fisheries Science and Food Production Processes, Qingdao National Laboratory for Marine Science and Technology, Qingdao, China; 3 College of Fisheries and Life Science, Shanghai Ocean University, Shanghai, China; 4 Joint Laboratory for Translational Medicine Research, Beijing Institute of Genomics, Chinese Academy of Sciences & Liaocheng People’s Hospital, Liaocheng, China; Kunming University of Science and Technology, CHINA

## Abstract

Albinism, a phenomenon characterized by pigmentation deficiency on the ocular side of Japanese flounder (*Paralichthys olivaceus*), has caused significant damage. Limited mRNA and microRNA (miRNA) information is available on fish pigmentation deficiency. In this study, a high-throughput sequencing strategy was employed to identify the mRNA and miRNAs involved in *P*. *olivaceus* albinism. Based on *P*. *olivaceus* genome, RNA-seq identified 21,787 know genes and 711 new genes by transcripts assembly. Of those, 235 genes exhibited significantly different expression pattern (fold change ≥2 or ≤0.5 and q-value≤0.05), including 194 down-regulated genes and 41 up-regulated genes in albino versus normally pigmented individuals. These genes were enriched to 81 GO terms and 9 KEGG pathways (p≤0.05). Among those, the pigmentation related pathways-Melanogenesis and tyrosine metabolism were contained. High-throughput miRNA sequencing identified a total of 475 miRNAs, including 64 novel miRNAs. Furthermore, 33 differentially expressed miRNAs containing 13 up-regulated and 20 down-regulated miRNAs were identified in albino versus normally pigmented individuals (fold change ≥1.5 or ≤0.67 and p≤0.05). The next target prediction discovered a variety of putative target genes, of which, 134 genes including Tyrosinase (TYR), Tyrosinase-related protein 1 (TYRP1), Microphthalmia-associated transcription factor (MITF) were overlapped with differentially expressed genes derived from RNA-seq. These target genes were significantly enriched to 254 GO terms and 103 KEGG pathways (p<0.001). Of those, tyrosine metabolism, lysosomes, phototransduction pathways, etc., attracted considerable attention due to their involvement in regulating skin pigmentation. Expression patterns of differentially expressed mRNA and miRNAs were validated in 10 mRNA and 10 miRNAs by qRT-PCR. With high-throughput mRNA and miRNA sequencing and analysis, a series of interested mRNA and miRNAs involved in fish pigmentation are identified. And the miRNA-mRNA regulatory network also provides a solid starting point for further elucidation of fish pigmentation deficiency.

## Introduction

Albinism, characterized by pigmentation deficiency in the skin, hair and eyes of mammals, has been extensively studied since the early 19th century [1, 2]. Visible pigmentation in mammals results from the biogenesis, transport and transfer of melanin, a type of pigment granule synthesized in the melanocyte [3]. During this process, more than 171 genes have been identified with a list at http://www.espcr.org/micemut, and their mutations have been reported to cause three main types of disorders: hyperpigmentation, hypopigmentation (albinism), or mixed hyper- /hypopigmentation [4–6]. There have been five enzymes identified as being required for human melanin pigmentation, namely tyrosinase (TYR), tyrosinase-related protein-1 (TYRP1), P (OCA2), solute carrier family 45 member 2 (SLC45A2), and G-protein coupled receptor 143 (GPR143), and defects in any of these enzymes could result in albinism [7]. Besides these important enzymes, three melanocyte-specific transcription factors also play central roles in the development, migration and differentiation of melanocytes: microphthalmia-associated transcription factor (MITF), paired box gene 3 (PAX3) and SRY-box containing gene 10 (SOX10) [7–10].

MicroRNAs (miRNAs), a type of non-coding RNA with a length between 22–24 nt, could act as post-transcriptional negative regulators of mRNA stability or translation [11, 12]. Since their initial discovery in the 1990s, miRNAs have been proven to extensively regulate cell differentiation, development and diseases [13–15]. In mammals, over 60% of mRNAs are thought to be regulated by miRNAs [16].

Since the first evidence of miRNA involvement in melanocyte biology was verified in 2010 [17], an increasing number of miRNAs have been found to participate in melanocyte development and diseases of mammals. For example, miR-140, miR-143, and miR-145 play important roles in targeting Dicer, a miRNA processing protein, during the development of the neural crest, from which melanocytes are derived [18, 19]. Additionally, miRNAs including miR-125b, miR-25, miR-137, miR-192, and miR-194 have been shown to regulate melanogenesis by reducing the expression level of MITF [20–23]. Recent studies have also revealed more than 30 miRNAs involved in the development and progression of melanoma, a type of skin cancer that originates in melanocytes [24–27].

Six types of chromatophores (melanophores, xanthophores, iridophores, leucophores, erythrophores and cyanophores) were discovered in fishes [28], suggesting that fish may possess more complicated pigmentation regulatory mechanisms than mammals. Previous studies of zebrafish (*Danio rerio*), medaka (*Oryzias latipes*), Mexican tetra (*Astyanax mexicanus*), and common carp (*Cyprinus carpio*) have identified that more than 100 genes participate in pigmentation [29–31], while only one miRNA has been confirmed to regulate pigmentation [32].

To date, limited studies are available reviewing pigmentation deficiencies of benthic marine fish such as Japanese flounder (*P*. *olivaceus*), a flatfish living in the coastal areas of China, Korea and Japan. Since the 1980s, an increasing frequency of albinism characterized by pigment deficiency on the ocular side of *P*. *olivaceus* has significantly reduced the commercial value and seriously hindered sustainable aquaculture development [33]. Although numerous reports have revealed the participation of miRNAs in the immune defences [34], metamorphosis [35–37], gonad development [38] and muscle development [39] of *P*. *olivaceus*, it remains unclear whether miRNAs play a role in pigmentation and albinism of *P*. *olivaceus*.

In present study, a high-throughput sequencing strategy was employed to screen differentially expressed mRNA and miRNAs between albino and normally pigmented *P*. *olivaceus*. Furthermore, the potential target genes of differentially expressed miRNAs were predicted by *in silico* analysis. The enrichment analysis of the GO term and KEGG pathway for these predicted genes and differentially expressed genes were subsequently conducted in order to gain insight into the function of mRNAs and miRNAs in *P*. *olivaceus* pigmentation deficiency.

## Materials and methods

### Ethical statement

The collection and handling of the animals used in this study was approved by the Animal Care and Use Committee at the Chinese Academy of Fishery Sciences, and all the experimental procedures were performed in accordance with the guidelines for the Care and Use of Laboratory Animals at the Chinese Academy of Fishery Sciences.

### Sample collection and RNA isolation

At the age of 5 months, *P*. *olivaceus* individuals from a full-sib family were obtained from Haiyang Yellow Sea Aquatic Product Co., Ltd. The fish were kept in seawater maintained at 20°C for one week. Skin tissue samples with an area of 2×2 cm^2^ from the ocular side of three normal and three albino individuals were collected and denoted as PO_con1, PO_con2, PO_con3, PO_alb1, PO_alb2, and PO_alb3, respectively. The samples were stored in liquid nitrogen for subsequent miRNA analysis.

The total RNA from each sample was extracted using Trizol reagent (Invitrogen, CA, USA) according to the manufacturer’s instructions. Total RNA integrity was assessed using the RNA Nano 600 Assay Kit of the Bioanalyzer 2100 system (Agilent, CA, USA) for quality control.

### RNA-seq sequencing, gene annotation and novel gene identification

A total amount of 3 μg RNA (RIN>7.0) per sample was used for cDNA paired-end libraries using NEBNext Ultra RNA Library Prep Kit for Illumina (NEB, USA) following manufacturer’s recommendations. In order to select cDNA fragments of 150-200bp in length, the library fragments were purified with AMPure XP system (Beckman Coulter, Beverly, USA). 3 μl USER Enzyme (NEB, USA) was used with size-selected, adaptor-ligated cDNA at 37°C for 15 min followed by 5 min at 95°C before PCR. Then PCR was performed with Phusion High-Fidelity DNA polymerase, Universal PCR primers and Index (X) Primer. At last, PCR products were purified (AMPure XP system) and library quality was assessed on the Agilent Bioanalyzer 2100 system. After cluster generation on a cBot Cluster Generation System using TruSeq PE Cluster Kit v3-cBot-HS (Illumina), the libraries were sequenced on an Illumina Hiseq2000 platform by Novogene Bioinformatics Technology Co. Ltd (Beijing, China) and 100 bp paired-end reads were generated.

The clean reads were obtained by removing reads containing adapter, reads containing Poly-N and low quality reads from raw reads. At the same time, Q20, Q30 and GC content of the clean reads were calculated.

The index of Japanese flounder genome was built using Bowtie v2.0.6 and clean reads were aligned to Japanese flounder genome using TopHat v2.0.9 for gene mapping and annotation.

The Cufflinks v2.1.1 Reference Annotation Based Transcript (RABT) assembly method was used to construct and identify both known and novel transcripts from TopHat alignment results.

### Differentially expressed genes identification and GO/KEGG enrichment analysis

The reads numbers mapped to each gene were counted using HTSeq v0.6.1. Then, RPKM of each gene was calculated based on the length of the gene and reads count mapped to this gene [40].

Differential expression analysis of samples was performed using the DESeq R package (1.10.1). The resulting p-values were adjusted using the Benjamini and Hochberg’s approach for controlling the false discovery rate. Genes with an adjusted p-value <0.05 found by DESeq were assigned as differentially expressed genes involved in *P*. *olivaceus* pigmentation.

Subsequently, based on the pigmentation genes list at http://www.espcr.org/micemut, previous publications [4, 7] and present study, the overlapped information were analysed and listed in a table.

Gene Ontology functional and KEGG pathway enrichment analysis of GeneCodis [41] was used to decipher the major biological processes and pathways involved by the differentially expressed genes. And GO terms and KEGG pathways with corrected p-value less than 0.05 were considered significantly enriched.

### Small RNA library construction, sequencing, and miRNA prediction

Approximately 1 μg RNA of an RNA integrity number (RIN) >7.0 from each sample was used for the subsequent cDNA library construction following the protocol of TruSeq Small RNA Sample Prep Kits (Illumina, San Diego, USA). Single-end sequencing (36 bp) was then performed on an Illumina HiSeq 2500 at the LC-BIO (Hangzhou, China) according to the vendor’s recommended protocol.

Initially, the raw reads were subjected to the Illumina pipeline filter (Solexa 0.3), and the datasets were further processed with an in-house program, ACGT101-miR (LC Sciences, Houston, Texas, USA), to remove adapter dimers, low complexity, common RNA families (rRNA, tRNA, snRNA, snoRNA) and repeats. Subsequently, the remaining clean reads were used to search against miRBase 21.0 (http://www.mirbase.org/search.shtml) for known miRNA prediction. During the alignment, length variation at both the 5’ and 3’ ends and one mismatch inside of the sequence were allowed. For the miRNAs identified in this study, the conservation analysis was conducted through comparisons with 40 species listed in S1 Table.

The unmapped sequences were further searched against *P*. *olivaceus* genomes [42] using RNAfold software (http://rna.tbi.univie.ac.at/cgi-bin/RNAWebSuite/RNAfold.cgi) for novel miRNA prediction. The criteria for secondary structure prediction were similar to a previous study [34].

### Differential analysis of miRNAs

To analyse the expression patterns of miRNAs among the control and albino groups, the frequency of miRNA counts was normalized with a modified global normalization method. Based on the normalization counts, the fold change was calculated as PO_alb versus PO_con, and the expression difference was measured with Student’s t test. The miRNAs with fold change ≥1.5 or fold change ≤0.67 and p ≤0.05 were considered significant. The pheatmap program of R software was utilized to display the differentially expressed miRNAs.

### Prediction and analysis of the target genes of miRNAs

The putative target genes were predicted utilizing two computational target prediction algorithms: TargetScan 6.2 (http://www.targetscan.org) and miRanda (http://www.microrna.org). The parameters of TargetScan and miRanda were set as score>50 and free energy<-10 kcal/mol, respectively. The overlap genes between the two algorithms were regarded as the target genes, whose enrichment analysis was conducted using GO terms (http://www.geneontology.org) and KEGG pathways (http://www.genome.jp/kegg). Based on these results, the miRNA-GO-network and miRNA-KEGG-network were generated. Cytoscape was used for displaying a visualized interaction between miRNAs and mRNAs.

### Real-time quantification of mRNA and miRNA

In order to validate the confidence of high-throughput transcriptome sequencing, 10 differentially expressed genes are selected and analyzed by quantitative real-time PCR assay. *P*. *olivaceus* β-actin gene was used as the internal reference. Primers were listed in S2 Table. The total RNAs for high-throughput transcriptome sequencing were reverse transcripted into cDNA by PrimeScript RT reagent Kit with gDNA Eraser (Takara, Japan). Then qRT-PCR were conducted using SYBR Premix Ex Taq (Takara, Japan) in 20 μl reaction solution containing 10 μl SYBR Premix Ex Taq (2X), 0.4 μl forward primer and 0.4 μl reverse primer, 0.4 μl ROX reference dye, 1μl cDNA and 8.2 μl ddH2O. The PCR amplification procedure was carried out at 95°C for 10 s; 40 cycles of 95°C for 5 s and 60°C for 34 s; followed by disassociation curve analysis in a ABI 7500 fast real-time PCR system (Applied Biosystems, USA). The amplification reaction without the template was used as a no template control. All reactions were performed in triplicate. The relative gene expression was calculated using the 2-ΔΔCt method [43]. Statistical comparison of the levels detected at different time points is carried out by SPSS, and p<0.05 were considered as significant.

To validate the differential expression results from miRNA sequencing, 10 miRNAs were randomly selected for qRT-PCR. Firstly, the total small RNAs from the normal and albino pigmented skins were extracted using a miRcute miRNA Isolation Kit (TianGen, Beijing, China). The poly(A) tail addition and reverse transcription for 2 μg RNA were performed using the miRcute miRNA First-Strand cDNA Synthesis Kit (Tiangen, Beijing, China). The qRT-PCR was then performed by the miRcute miRNA qPCR Detection Kit (SYBR) (Tiangen, Beijing, China). Next, PCRs consisting of 20 μl PCR reaction solution containing 1 μl cDNA, 10 μl 2×miRcute miRNA premix, 1.6 μl 50×ROX Reference Dye, 0.4 μl forward primer, 0.4 μl reverse primer, and 6.6 μl ddH_2_O were carried out on an ABI Prism 7500 Fast Sequence Detection System (ABI, Carlsbad, CA, USA) at 94°C for 2 min, followed by 40 cycles of 94°C for 20 s and 60°C for 34 s. Forward primers were designed based on mature miRNA sequences (S3 Table), and the reverse primer was provided by the miRcute miRNA qPCR Detection Kit (SYBR) (Tiangen, Beijing, China). The 5s rRNA of *P*. *olivaceus* was used as the internal control for the normalization of expression levels. The results were analysed using the 2^-ΔΔCt^ method, with the threshold cycle (Ct) determined by the default threshold settings. The experiment was conducted independently three times.

## Results

### RNA-seq sequencing and analysis

In order to better understand *P*. *olivaceus* albinism mechanism, we conducted a comparative transcriptomic analysis among three normal and albino pigmented individuals. A total of six cDNA libraries, named as PO_con1, PO_con2, PO_con3, PO_alb1, PO_alb2 and PO_alb3, were constructed and sequenced. A mean of 31,655,702 clean reads with Q20 higher than 94.17% were obtained from each library (S4 Table, S1 Fig). Further genome mapping analysis revealed that over 79.13% of reads were mapped with *P*. *olivaceus* genome. Subsequently, a total of 21787 know genes and 711 new genes were identified by further transcripts assembly based on *P*. *olivaceus* genome.

### Differentially expressed genes identification and validation

**Fig 1:**
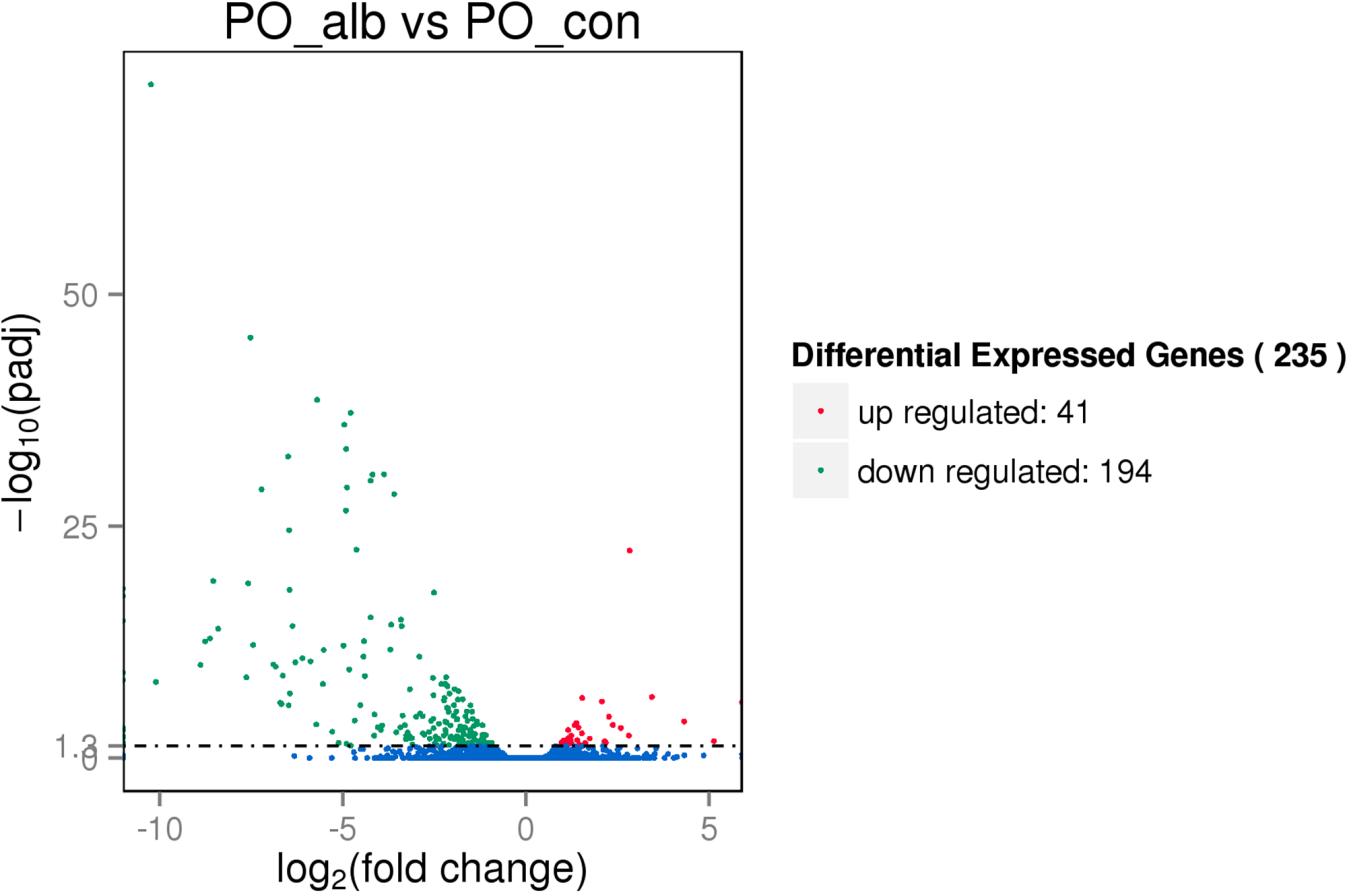
The volcanoplot of differentially expressed genes in RNA-seq. The differentially expressed genes including 41 up-regulated genes and 194 down-regulated genes in PO_alb vs PO_con were illustrated in a volcanoplot (fold change≥2 and q-value≤0.05).

Based on the expression level of each gene in samples, the correlation coefficient was calculated to evaluate the repeatability. The result showed that the pearson correlation coefficient between three replicas was 95.0~97.5% (S2 Fig). The subsequent differential expression showed that 235 genes exhibited significantly different (Fig 1, S5 Table) (fold change≥ 2 or ≤ 0.5 and q-value ≤ 0.05), including 194 down-regulated genes and 41 up-regulated genes in PO_alb vs PO_con.

**Fig 2:**
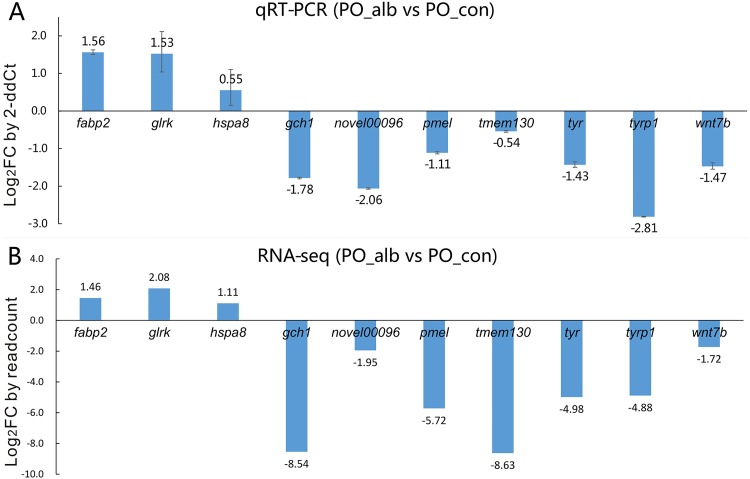
The expression levels of ten genes in qRT-PCR and RNA-seq. The expression fold change (FC) 10 *P*. *olivaceus* genes in PO_alb versus PO_con detected by qRT-PCR and RNA-seq were calculated by 2-ddCt and readcount, respectively. And these genes’ log2FC values of qRT-PCR and RNA-seq are shown in A and B, respectively. In qRT-PCR analysis, β-actin expression levels were used for the internal control, and values are indicated as means ± standard error (SE) derived from triplicate experiments.

Subsequently, 10 genes, including up-regulated genes *fatty acid-binding protein*, *intestinal (fabp2)*, *probable glutamate receptor (glrk)*, *heat shock cognate 71 kDa protein (hspa8)*, and down-regulated genes *GTP cyclohydrolase 1 (gch1)*, *novel00096*, *melanocyte protein (pmel)*, *transmembrane protein 130 (tmem130)*, *tyr*, *tyrp1*, *wnt7b* were selected for qRT-PCR validation experiment. The relative expression levels of these genes were similar with those of high-throughput transcriptome results (Fig 2).

### The overlapped pigmentation related genes between previous publications and present study

In order to further confirm the fidelity of the differentially expressed genes presented in this study, we compared the overlapped pigmentation related genes between previous publications and present study. And the comparison revealed that 30 genes including Endothelin B receptor (EDNRB), GCH1, Mast/stem cell growth factor receptor (KIT), melanocortin 1 receptor (MC1R), MC5R, MITF, OCA2, PAX7, SLC45A2, SOX10, TYR, TYRP1, WNT7B were overlapped, which all showed down-regulated expression pattern in PO_alb vs PO_con (Table 1).

**Table 1:** The overlapped pigmentation related genes between previous study and present study.

Index	Gene_ID	Gene symbol, description and chromosome location	PO_con (readcount)	PO_alb (readcount)	log2 fold change (PO_alb vs PO_con)	padj
1	GS_012501	BMP1, Bone morphogenetic protein 1, chro13	16.47	1.06	-3.96	0.000954
2	GS_012647	EDNRB, Endothelin B receptor, chro11	418.19	166.60	-1.33	0.000218
3	GS_018359	EDNRB, Endothelin B receptor, chro12	561.53	263.69	-1.09	0.003513
4	GS_005903	GCH1, GTP cyclohydrolase 1, chro24	284.36	0.00	Inf	1.65E-15
5	GS_008748	GCH1, GTP cyclohydrolase 1, chro4	803.68	2.16	-8.54	8.45E-20
6	GS_005902	GCH1, GTP cyclohydrolase 1, chro24	1056.71	3.11	-8.41	1.18E-14
7	GS_013228	GCH1, GTP cyclohydrolase 1, chro3	187.56	1.07	-7.45	6.61E-13
8	GS_009046	KIT, Mast/stem cell growth factor receptor, chro5	76.74	17.25	-2.15	3.79E-06
9	GS_018838	K1C13, Keratin, type I cytoskeletal 13, chro10	117.72	30.17	-1.96	2.05E-06
10	GS_014679	K1C13, Keratin, type I cytoskeletal 13, chro5	29391.00	10648.78	-1.46	0.018738
11	GS_012545	K1C18, Keratin, type I cytoskeletal 18, chro13	144.15	5.82	-4.63	3.57E-23
12	GS_007378	MC1R, Melanocortin 1 receptor, chro7	27.32	3.99	-2.78	0.003166
13	GS_011397	MC5R, Melanocortin 5 receptor, chro6	27.61	0.00	Inf	6.14E-10
14	GS_018288	MCHR2, Melanin-concentrating hormone receptor 2, chro12	23.23	2.35	-3.30	0.000297
15	GS_002043	MITF, Microphthalmia-associated transcription factor, chro1	33.44	0.33	-6.68	1.54E-06
16	GS_000869	MAR1, Melanoma antigen recognized by T-cells 1, chro13	277.19	14.69	-4.24	1.26E-30
17	GS_004597	MREG, Melanoregulin, chro10	154.35	33.53	-2.20	1.01E-08
18	GS_001946	MYO5A, Myosin-Va, chro7	296.08	151.63	-0.97	0.047734
19	GS_017193	OCA2, P protein, chro24	21.26	1.40	-3.92	0.000309
20	GS_003876	PAX7, Paired box protein Pax-7, chro17	88.93	40.55	-1.13	0.041166
21	GS_003590	PMEL, Melanocyte protein, chro1	210.22	3.98	-5.72	0.000246
22	GS_012916	SLC45A2, Membrane-associated transporter protein, chro13	35.13	5.84	-2.59	0.000102
23	GS_012661	SOX10, Transcription factor SOX-10, chro10	270.45	81.67	-1.73	4.55E-07
24	GS_014478	TBX19, T-box transcription factor 19, chro14	12.77	0.33	-5.29	0.001515
25	GS_020582	TRPM1, Transient receptor potential cation channel subfamily M member 1, chro19	222.04	57.56	-1.95	3.80E-08
26	GS_020975	TYR, Tyrosinase, chro21	50.90	1.61	-4.98	7.81E-13
27	GS_009038	TYRP1, Tyrosinase-related protein 1, chro5	289.28	9.33	-4.95	1.16E-36
28	GS_012029	TYRP1, Tyrosinase-related protein 1, chro9	292.57	9.91	-4.88	6.72E-30
29	GS_021046	WNT7B, Protein Wnt-7b, chro16	40.65	12.32	-1.72	0.02056
30	GS_014490	XDH, Xanthine dehydrogenase/oxidase, chro14	91.58	7.17	-3.68	4.31E-15

### The GO term and KEGG pathway enrichment analysis of RNA-seq

**Fig 3:**
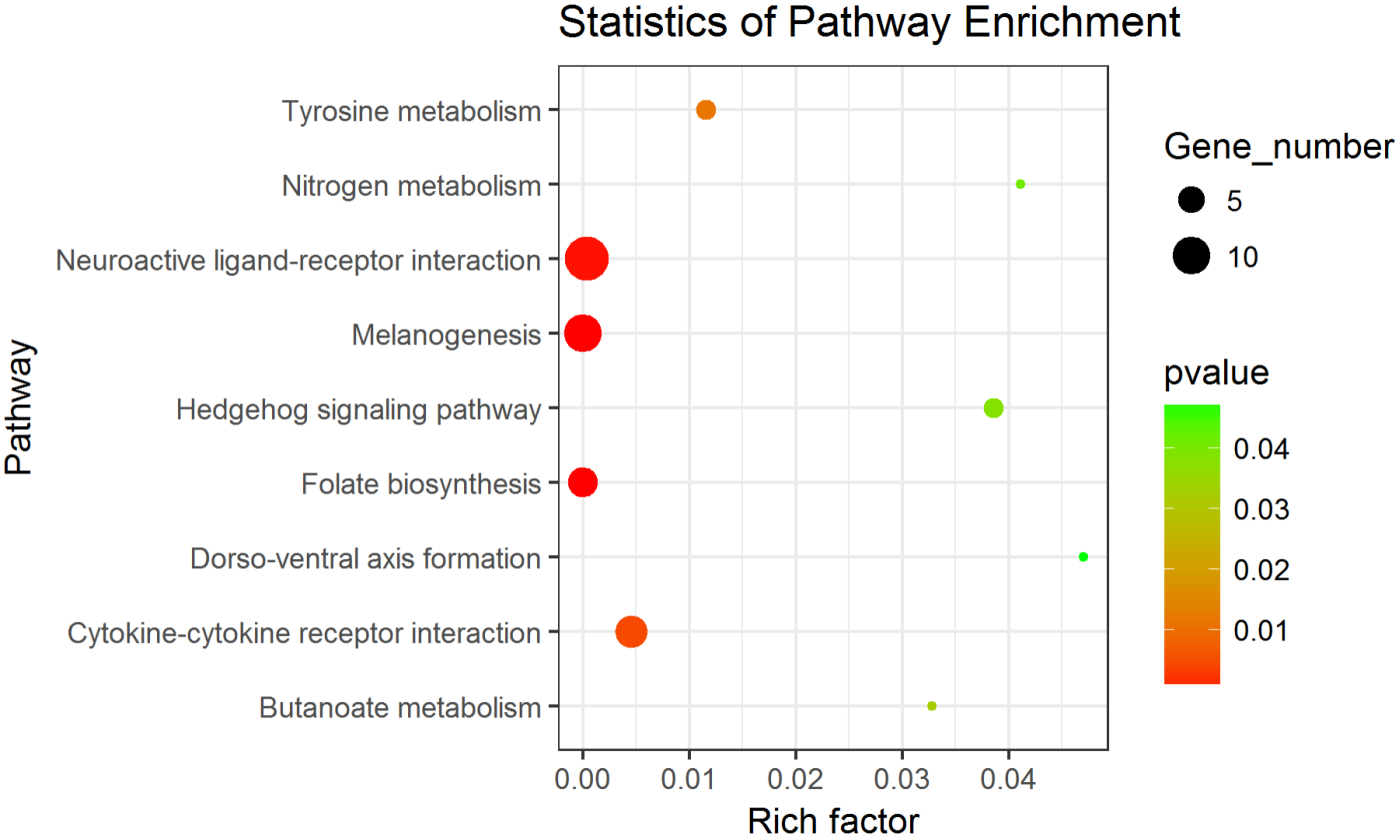
KEGG pathways enrichment in RNA-seq (p<0.05). Gene number: number of target genes in each pathway. Rich factor: the ratio of the number of target genes divided by the number of all the genes in each pathway.

In order to further understand the function of these differentially expressed genes, GO term and KEGG pathway were subsequently carried out. GO term enrichment analysis revealed that 81 GO terms with p≤0.05 were enriched, including extracellular region, transmembrane signalling receptor activity, G-protein coupled receptor signalling pathway, signal transducer activity, molecular transducer activity, cell surface receptor signalling pathway, G-protein coupled receptor activity, heme oxygenase (decyclizing) activity, heme oxidation, receptor activity and so on (S6 Table). While, no GO terms with q≤0.05 were enriched. The KEGG pathway enrichment analysis showed that 9 pathways with p≤0.05 were significantly enriched, including Folate biosynthesis, Melanogenesis, Neuroactive ligand-receptor interaction, Tyrosine metabolism, and so on (Fig 3, S7 Table). Of those, the top three pathways exhibited a more significant difference with q≤0.05.

### MicroRNA library construction and identification

**Fig 4:**
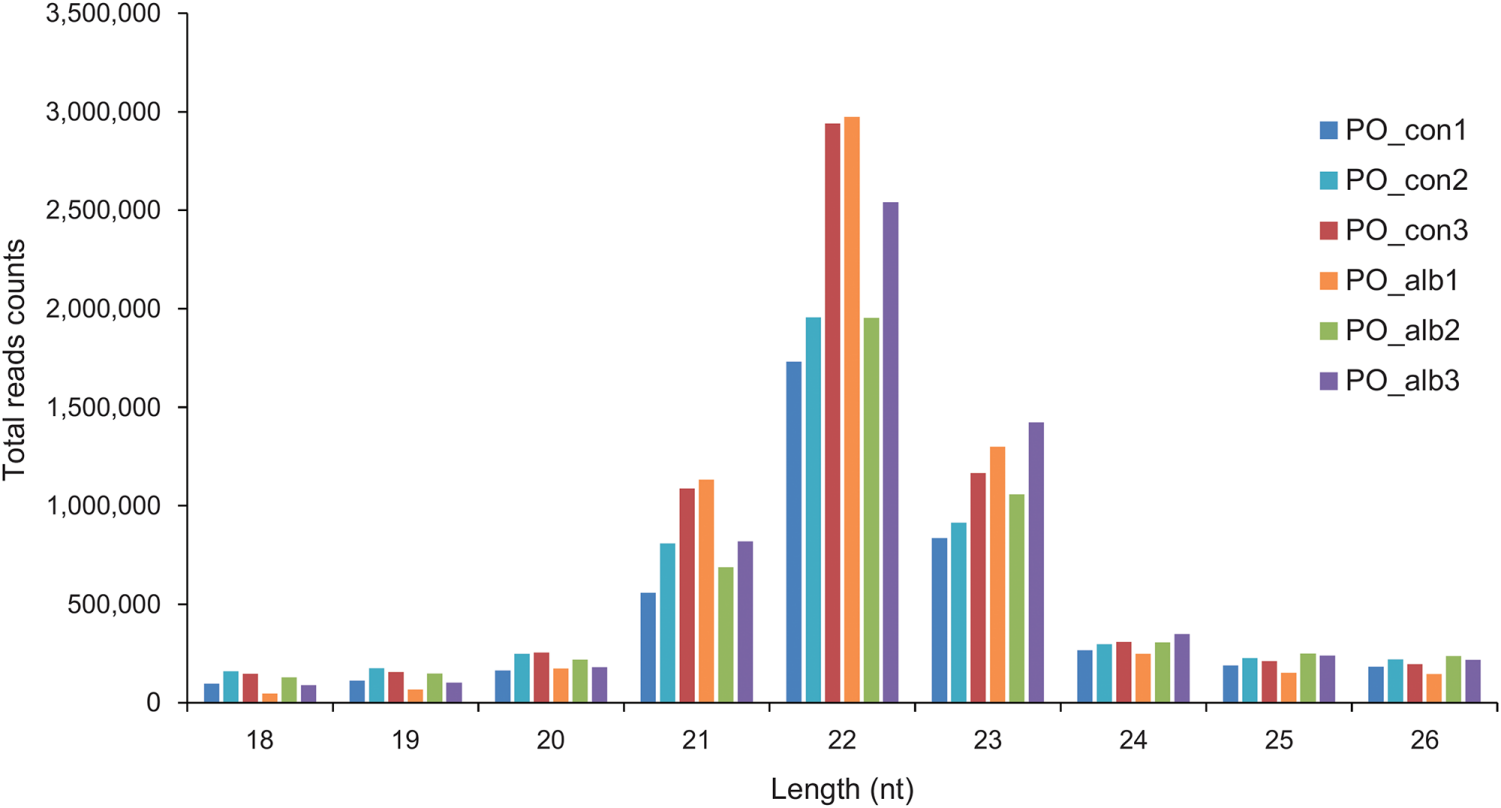
Length distribution of small RNAs. The clean reads of small RNAs were distributed from 18 nt to 26 nt in six libraries including PO_con1, PO_con2, PO_con3, PO_alb1, PO_alb2, and PO_alb3. The reads of 20–24 nt accounted for 84–93% of small RNAs.

To identify miRNAs involved in the albinism of *P*. *olivaceus*, six small RNA libraries, PO_con1, PO_con2, PO_con3, PO_alb1, PO_alb2 and PO_alb3, were constructed and sequenced using Illumina HiSeq 2500. A total of 9,960,534, 10,957,008, 11,060,368, 10,559,741, 9,702,427, and 10,974,117 raw reads were acquired, respectively. The quality scores across all bases before and after trimming showed high accuracy (>99.9%) in all six libraries (S3 Fig). These reads were first adjusted to remove the sequencing artefacts: reads without a 3’ adaptor, reads that were <18 nt and >26 nt, and junk reads (≥2N, ≥7A, ≥8C, ≥6G, ≥7T, ≥10Dimer, ≥6Trimer, or ≥5Tetramer). Furthermore, these sequences were mapped to the RFam database (http://rfam.janelia.org), *P*. *olivaceus* mRNA database [42], and Repbase (http://www.girinst.org/repbase) for filtering the non-coding RNA families except miRNA, mRNA, and repetitive sequences. As a result of data treatment, 4,134,252 (41.51%), 5,004,942 (45.68%), 6,467,034 (58.47%), 6,238,636 (59.08%), 4,989,758 (51.43%), and 5,955,979 (54.27%) clean reads were obtained from the total reads of PO_con1, PO_con2, PO_con3, PO_alb1, PO_alb2 and PO_alb3, respectively (S8 Table). The length analysis indicated that 84–89% of clean read sequences were distributed between 20 and 24 nt (Fig 4), in accordance with the typical characteristics of Dicer processing products [44].

The biological replicate quality check revealed that one albino sample (PO_alb1) exhibited a poor correlation with the other two samples, with R = 0.648–0.668 (S4 Fig). Hence, PO_alb1 and PO_con1 were excluded from subsequent miRNA identification and expression pattern analysis.

### Identification of conserved miRNAs

The clean reads from PO_con2, PO_con3, PO_alb2, and PO_alb3 were aligned with miRbase 21.0 and the *P*. *olivaceus* genome & EST, and 411 conserved miRNAs with normalized reads ≥10 were identified in *P*. *olivaceus*. According to the mapping information, four groups were clustered: gp1a, gp1b, gp2, and gp3. Group 1, gp1a, included 31 unique miRNAs mapping to *P*. *olivaceus* miRNA/pre-miRNAs in miRbase, and the pre-miRNAs further mapped to the genome & EST (S9 Table). Group 2, gp1b, included 240 unique miRNAs mapping to selected species’ (except for *P*. *olivaceus*) miRNAs/pre-miRNAs in miRbase, and the pre-miRNAs further mapped to the genome & EST (S10 Table). Group 3, gp2, included 75 unique miRNAs mapping to selected species’ (except for *P*. *olivaceus*) miRNAs/pre-miRNAs in miRbase, while only miRNA (not pre-miRNA) was mapped to the genome (S11 Table). Group 4, gp3, included 65 unique miRNAs mapping to selected species’ (except for *P*. *olivaceus*) miRNAs/pre-miRNAs in miRbase, but neither pre-miRNA nor miRNA was mapped to the genome (S12 Table).

### The conservation of the identified miRNAs with other species

**Fig 5:**
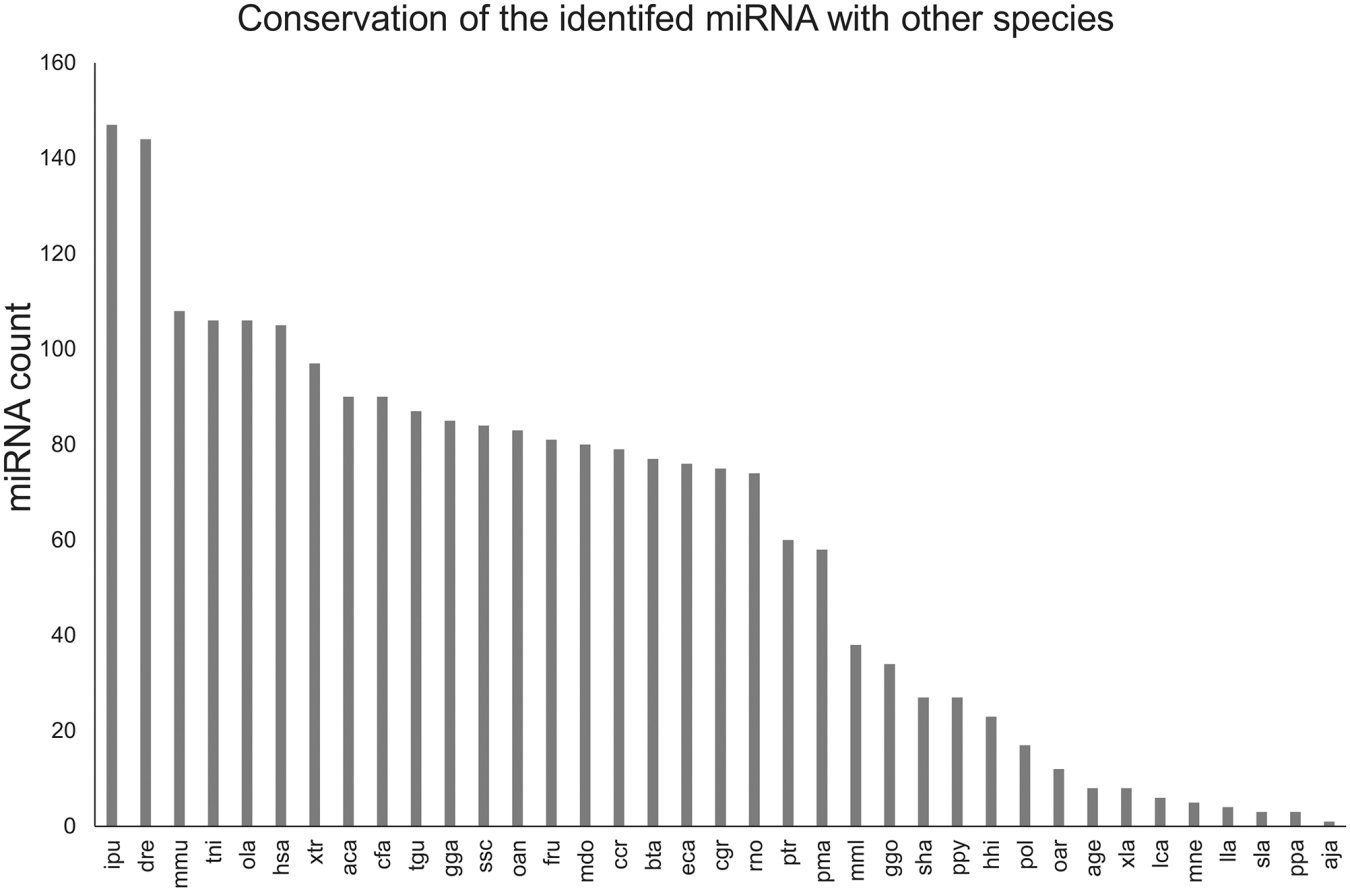
Conservation profile of the identified miRNA. The frequency of identified miRNAs is calculated by mapping with 37 species (horizontal axis) in miRbase, and the miRNA counts are shown on the vertical axis.

The frequency with which 411 conserved miRNAs occurred in other species was calculated, and the result (Fig 5) showed that the top five species were channel catfish (*Ictalurus punctatus*), zebrafish (*Danio rerio*), house mouse (*Mus musculus*), Tetraodon pufferfish (*Tetraodon nigroviridis*), and Japanese killifish (*Oryzias latipes*), with 147, 144, 108, 106 and 106 conserved miRNAs, respectively.

### Identification of putative novel miRNAs

**Fig 6:**
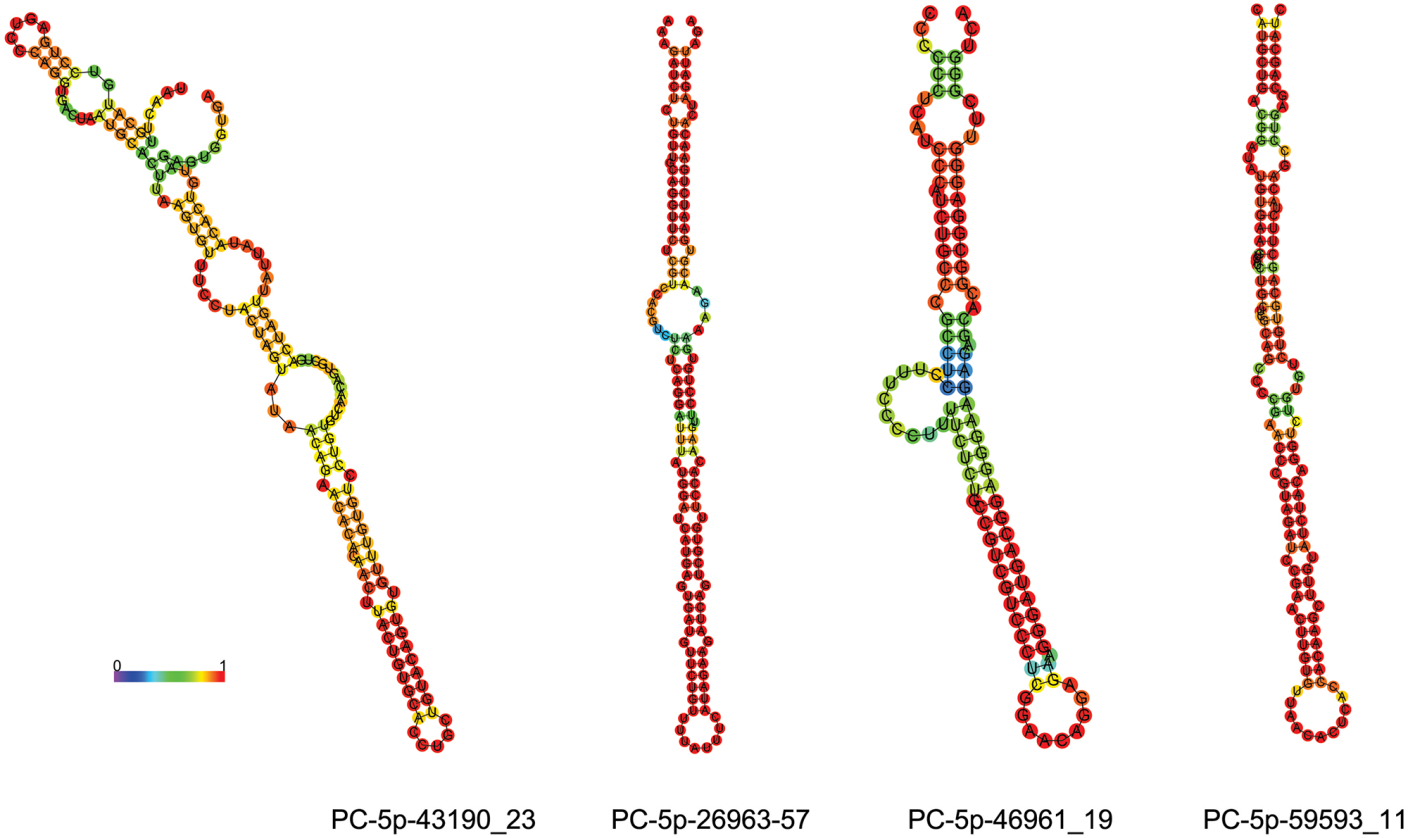
The illustration of four novel miRNAs in *P*. *olivaceus*. The secondary structures formed by four novel miRNA precursors are illustrated by RNAfold. The colour bar represents base-pair probabilities from 0 (blue) to 1 (red).

Following the identification of conserved miRNAs, the unannotated sequences were matched to *P*. *olivaceus* genomes [42] and analysed using RNAfold software. Consequently, 64 putative novel miRNAs were identified (S13 Table), and the hairpin structures formed by the precursor sequences of four novel miRNAs are illustrated in Fig 6.

### miRNA differential expression profiles

By combining the conserved and novel miRNAs, a total of 475 miRNAs were identified. The subsequent differential expression revealed 33 significantly differentially expressed miRNAs in albino versus normally pigmented *P*. *olivaceus* (fold change ≥1.5 or ≤0.67 and p≤0.05), including 13 up-regulated miRNAs and 20 down-regulated miRNAs (Table 2).

**Table 2:** Differential expression of miRNAs in albino and normal Japanese flounder (p<0.05).

Index	miRNA name	miRNA sequence	up/down (PO_alb vs PO_con)	fold change (PO_alb vs PO_con)	pvalue (t_test)	PO_con (mean)	PO_alb (mean)
1	dre-miR-25-3p	CATTGCACTTGTCTCGGTCTGA	up	1.70	4.22E-02	10,546	17,961
2	hsa-miR-205-5p_R+2	TCCTTCATTCCACCGGAGTCTGTT	up	1.79	3.23E-02	527	942
3	hsa-miR-27a-3p_R+1	TTCACAGTGGCTAAGTTCCGCG	up	2.55	3.75E-03	11	28
4	mmu-miR-143-5p_R+2	GGTGCAGTGCTGCATCTCTGGTC	up	1.93	4.19E-02	78	151
5	oan-miR-139-3p_R-1_1ss8AC	TGGAGACCCAGCTCTGTTGGA	up	2.42	4.09E-02	20	48
6	ola-miR-30c_1ss21AG	TGTAAACATCCTACACTCTCGGC	up	1.61	3.33E-02	1,262	2,031
7	ola-miR-99_R-1	CAAGCTCGCCTCTGTGGGTCT	up	2.75	3.08E-02	283	778
8	PC-5p-46961_19	CCCCTTTTCTCTGCCGTCGTCCC	up	1.98	4.45E-02	10	20
9	PC-5p-59593_11	GGACCCGTAGATCCGAACTTGT	up	2.60	1.66E-02	7	19
10	pol-miR-199a-5p_R+1	CCCAGTGTTCAGACTACCTGTTC	up	2.71	2.91E-02	129,672	351,541
11	tni-miR-205	TCCTTCATTCCACCGGAGTCTG	up	1.82	4.64E-02	60,815	110,934
12	xtr-miR-222_R-1	AGCTACATCTGGCTACTGGGTCT	up	1.94	3.53E-02	3,066	5,950
13	xtr-miR-92a_R+4	TATTGCACTTGTCCCGGCCTGTTTT	up	2.41	2.84E-02	370	893
14	dre-miR-18b-5p_1ss11TC	TAAGGTGCATCTAGTGCAGATA	down	0.39	6.23E-03	127	50
15	dre-miR-202-5p_R-1	TTCCTATGCATATACCTCTTT	down	0.07	1.37E-02	13	1
16	dre-miR-203b-3p_L-1R+2_1ss11CT	TGAAATGTTTAGGACCACTTGAT	down	0.67	3.87E-02	90	60
17	dre-miR-204-5p_L+1	TTTCCCTTTGTCATCCTATGCCT	down	0.20	3.73E-02	358	70
18	dre-miR-204-5p_R+2	TTCCCTTTGTCATCCTATGCCTGT	down	0.30	8.67E-03	61	18
19	dre-miR-20a-3p_2ss7GA11GA	ACTGCAATGTAAGCACTTGAAG	down	0.54	3.79E-02	88	47
20	dre-miR-26a-5p_R-1_1ss21CT	TTCAAGTAATCCAGGATAGGT	down	0.48	3.89E-02	632	302
21	dre-miR-9-3p_R+1	TAAAGCTAGATAACCGAAAGTA	down	0.38	3.35E-02	10	4
22	hhi-miR-26_R+1	TTCAAGTAATCCAGGATAGGCT	down	0.55	2.92E-02	31,645	17,508
23	oan-miR-16b-5p_1ss22GA	TAGCAGCACGTAAATATTGGTA	down	0.38	3.00E-02	39	15
24	ola-miR-106a_R+2	TAAAGTGCTTACAGTGCAGGTAG	down	0.43	2.68E-02	1,524	649
25	ola-miR-135b_R+3	TATGGCTTTTTATTCCTACGTG	down	0.21	2.39E-02	55	11
26	ola-miR-16	TAGCAGCACGTAAATATTGGC	down	0.47	6.27E-03	6,999	3,285
27	ola-miR-205_L-1R+1_1ss20TA	GATTTCAGTGGTGTGAAGAGTA	down	0.57	4.18E-02	257	146
28	PC-5p-26963_57	TATGGATCATGAGTGATGTTCT	down	0.45	1.15E-02	24	11
29	PC-5p-43190_23	TAACAGAACACACAACTTACTG	down	0.60	3.46E-02	15	9
30	pma-miR-1c-3p_1ss2GT	TTGAATGTAAAGAAGTATGTAC	down	0.43	3.62E-02	74	32
31	tni-miR-135b_R+1	TATGGCTTTTTATTCCTATCTGA	down	0.19	3.14E-02	35	7
32	tni-miR-15a_R-1	TAGCAGCACGGAATGGTTTGT	down	0.46	4.88E-02	9,843	4,536
33	tni-miR-16_R-1	TAGCAGCACGTAAATATTGGA	down	0.43	3.59E-02	11,582	4,964

**Fig 7:**
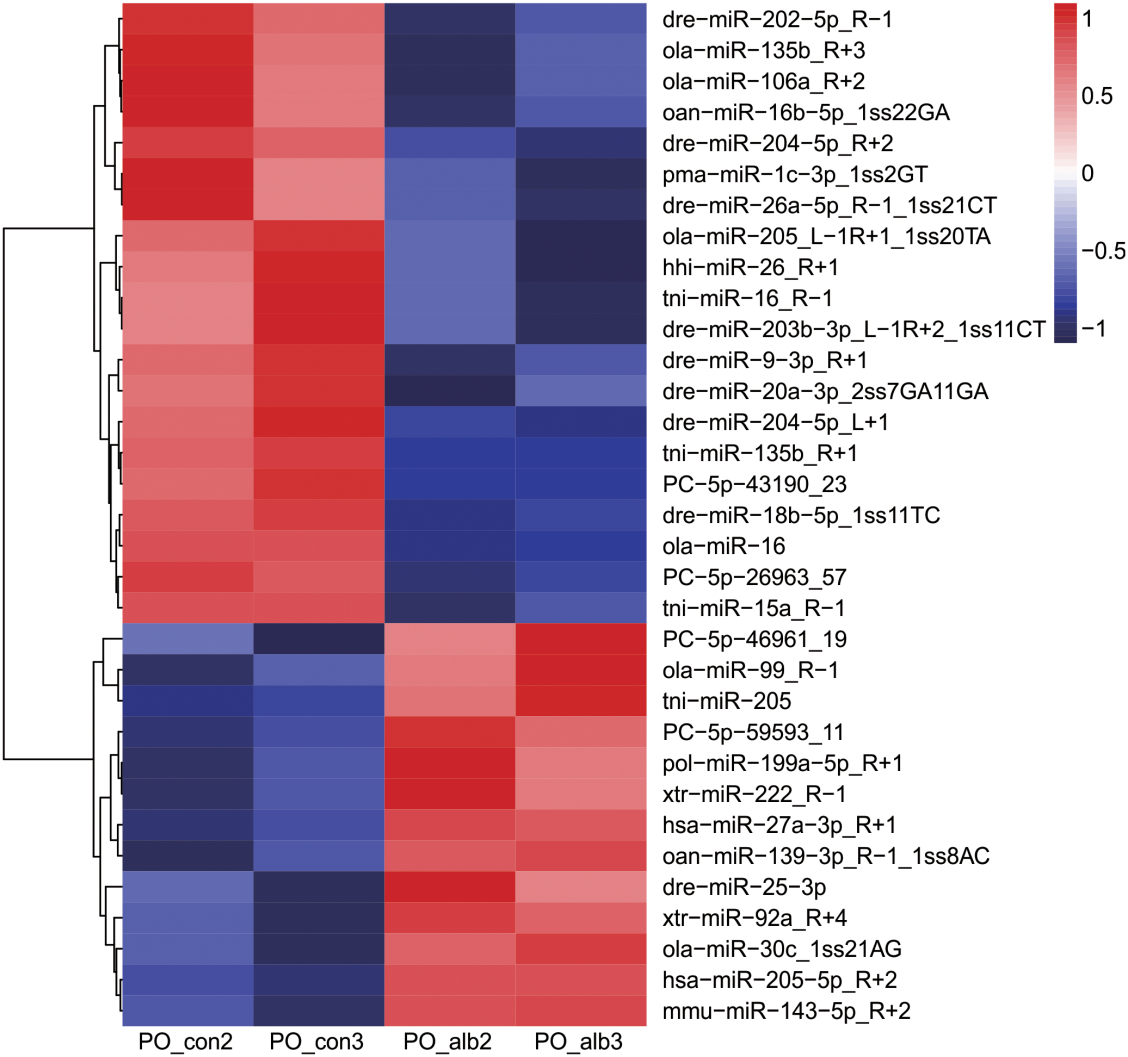
Clustering of expression patterns of 33 differentially expressed miRNAs. The expression patterns of 33 differentially expressed miRNAs (p<0.05) in the PO_con2, PO_con3, PO_alb2, and PO_alb3 libraries are displayed in a pheatmap. Each column represents one library, and the colour bar indicates relative expression level from high (red) to low (green).

A heat map based on the miRNA differential expression pattern (Fig 7) showed two clusters of miRNAs, 13 up-regulated miRNAs and 20 down-regulated miRNAs. Further clustering revealed that two sub-clusters were identified within the down-regulated miRNAs: one contained 7 miRNAs, and the other contained 13 miRNAs (Fig 7). Additionally, the up-regulated miRNAs also contained two sub-clusters, one consisting of 3 miRNAs and the other consisting of 10 miRNAs (Fig 7).

### Real-time quantitative RT-PCR

**Fig 8:**
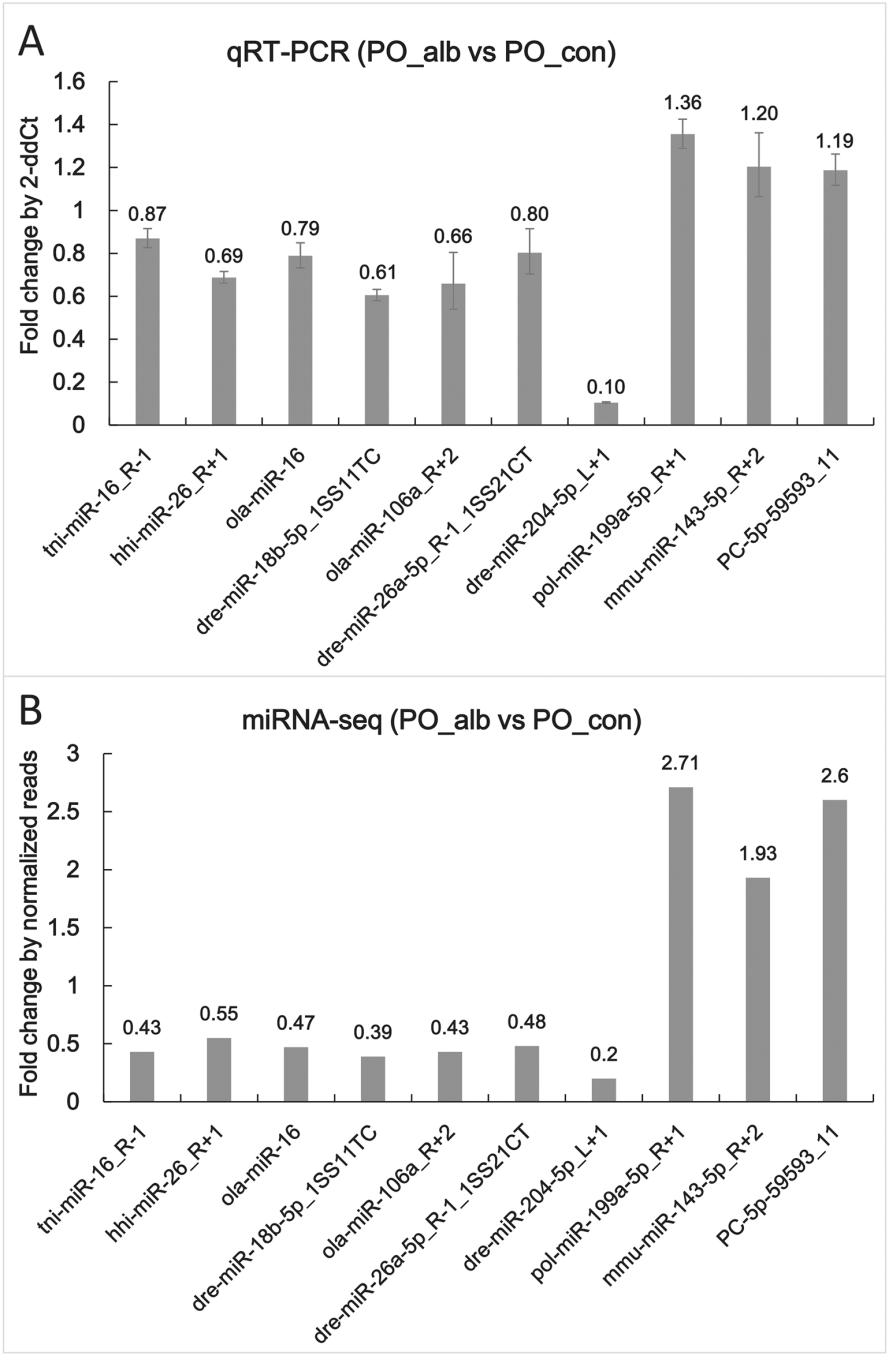
The expression levels of 10 randomly selected miRNAs in qRT-PCR and miRNA-seq. The expression fold changes of 10 *P*. *olivaceus* miRNAs in PO_alb versus PO_con detected by poly (A)-tailed qRT-PCR and high-throughput sequencing are shown in A and B, respectively. In qRT-PCR analysis, 5s rRNA expression levels were used for the internal control, and values are indicated as means ± standard error (SE) derived from triplicate experiments.

To validate the expression patterns of the miRNAs, qRT-PCR was utilized to detect the expression of 10 randomly selected miRNAs in which both up-regulated and down-regulated miRNAs were included. The three up-regulated miRNAs were pol-miR-199a-5p_R+1, mmu-miR-143-5p_R+2 and PC-5p-59593_11. The seven down-regulated miRNAs were tni-miR-16_R-1, ola-miR-26_R+1, ola-miR-16, dre-miR-18b-5p_1ss11TC, ola-miR-106a_R+2, dre-miR-26a-5p_R-1_1ss21CT and dre-miR-204-5p_L+1. These results verified the similar expression pattern trends of all 10 miRNAs between qRT-PCR and miR-seq (Fig 8), although there were slight differences in fold-change.

### Target prediction for significantly differentially expressed miRNAs and function analysis

To provide clues for the possible roles of 33 differentially expressed miRNAs, target prediction analysis was conducted by TargetScan 6.2 and miRanda algorithms. These algorithms identified 14655 putative target genes, including genes that participate in the trafficking of melanosomes such as melanophilin (MELPH) and melanotransferrin (MFI2); genes coding various proteins involved in melanin synthesis such as TYR, TYRP1, TYRP2, MC1R, and tyrosine 3-monooxygenase (TH); and melanocyte-specific transcription factors such as MITF, transcription factor 7-like 2 (TCF7L2), and paired box protein Pax-3-B (PAX3B) [7].

**Fig 9:**
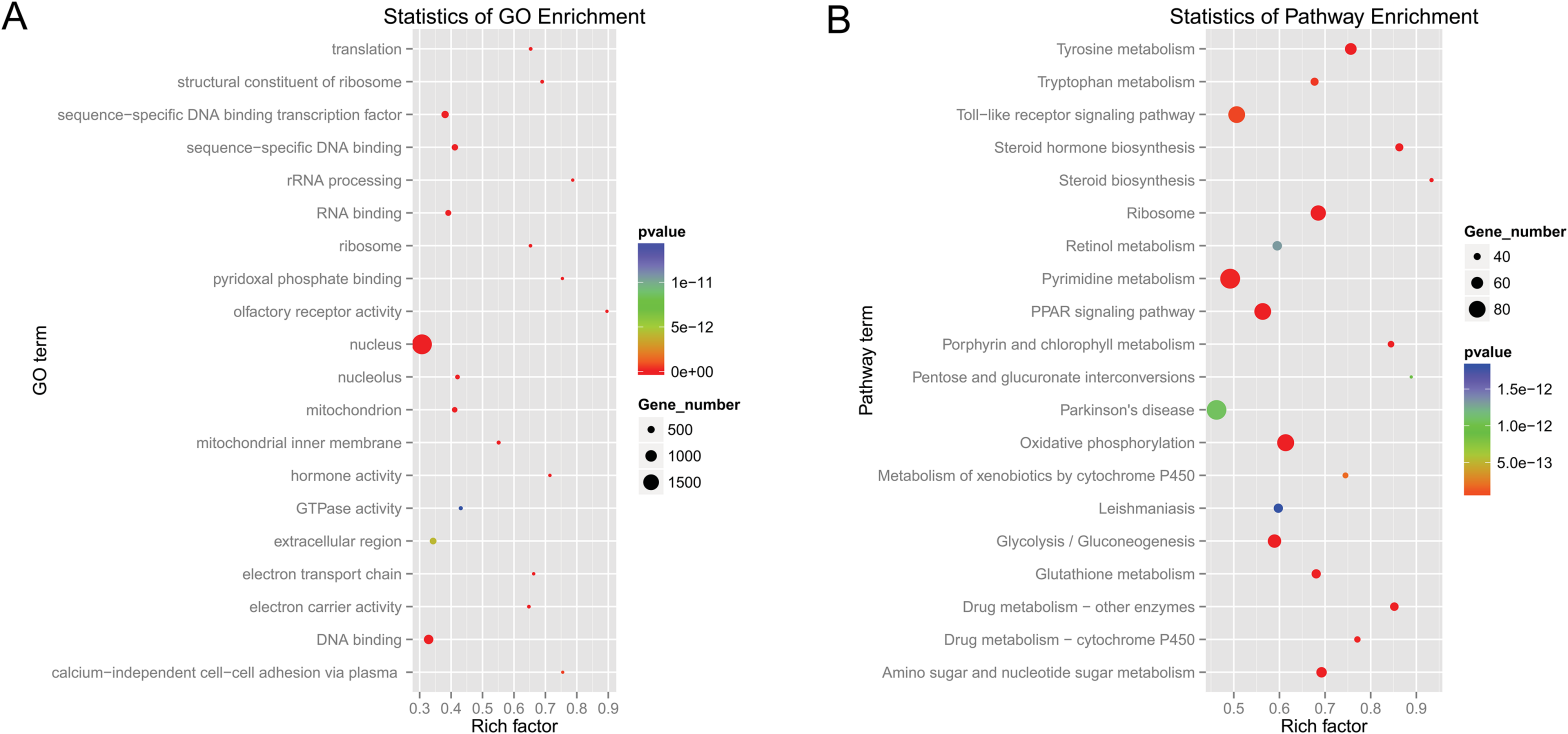
The top 20 enriched GO terms (A) and KEGG pathways (B). Gene number: number of target genes in each term or pathway. Rich factor: the ratio of the number of target genes divided by the number of all the genes in each term or pathway.

To further interpret the possible physiological processes and pathways regulated by these identified miRNAs, their putative target genes were subjected to GO term and KEGG pathway analysis. GO term analysis indicated that a total of 254 out of the 7263 clustered GO terms were significantly enriched (p<0.001) (S14 Table). The top 20 enriched terms were mainly involved in biological processes (translation, rRNA processing, electron transport chain, calcium-independent cell-cell adhesion via plasma membrane cell-adhesion molecules), cellular components (mitochondrion, mitochondrial inner membrane, ribosome, nucleus, nucleus, nucleolus, extracellular complex) and molecular functions (sequence-specific DNA binding, sequence-specific DNA binding transcription factor activity, structural constituent of ribosome, electron carrier activity, olfactory receptor activity, hormone activity, RNA binding, pyridoxal phosphate binding, DNA binding, GTPase activity) (Fig 9A).

Using KEGG pathway analysis, 14655 predicted genes were grouped into 256 pathways. Of these 256 pathways, 103 pathways were significantly enriched (p<0.001) (S15 Table), including tyrosine metabolism, PPAR signalling pathways, lysosomes, and phototransduction. The top 20 enriched pathway terms are shown in Fig 9B.

**Fig 10:**
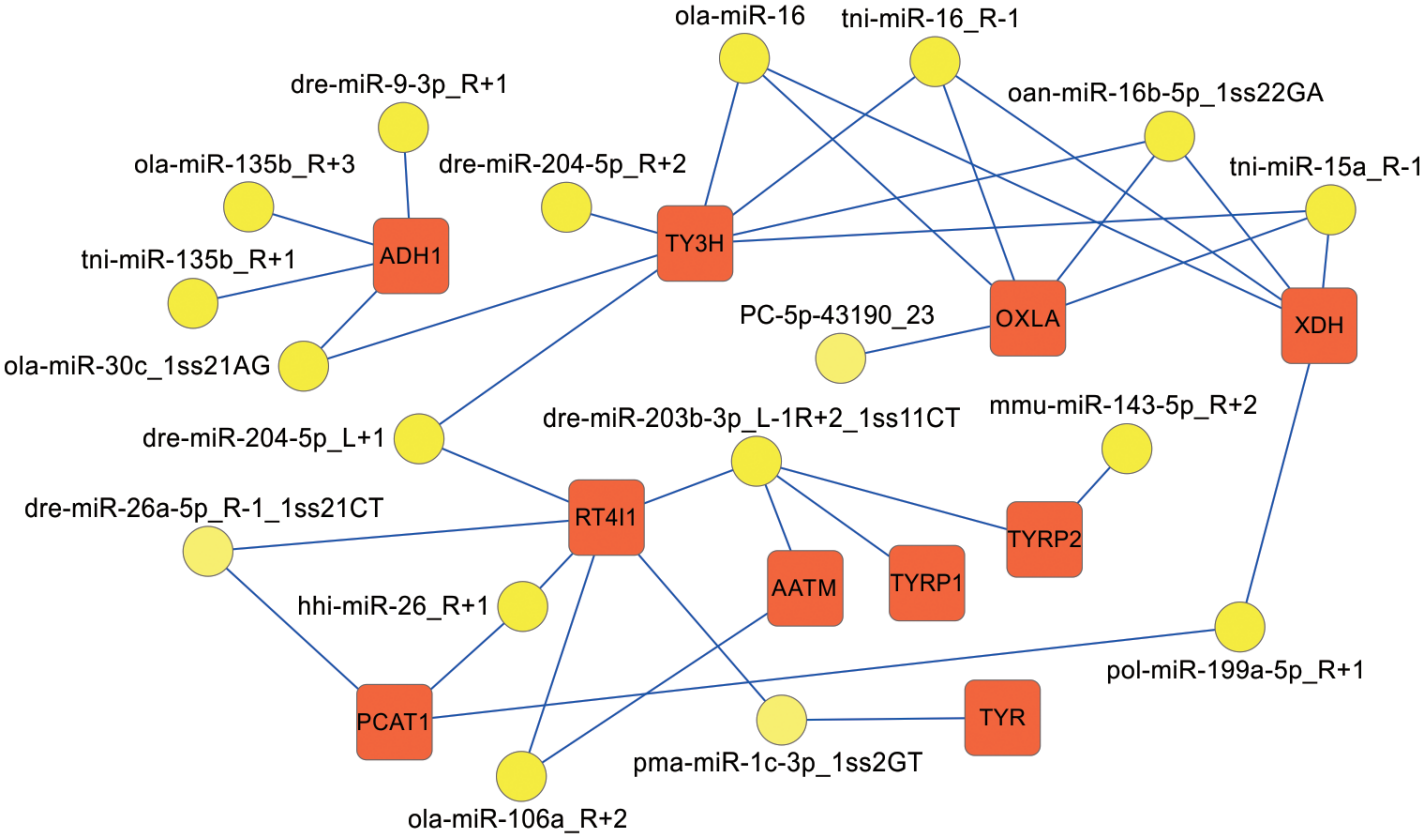
A proposed network of putative interactions between miRNAs and mRNAs in tyrosine metabolism. The regulation network of miRNAs and mRNAs involved in tyrosine metabolism is illustrated by Cytoscape. Yellow ellipses represent miRNAs, and red rectangles indicate their target genes associated with tyrosine metabolism. Abbreviations of the genes are detailed in the text.

Based on these results, the relevant miRNA-GO-network and miRNA-KEGG-network were finally generated. For instance, for tyrosine metabolism, TYR was regulated by pma-miR-1c-3p-1ss2GT; TYRP1 was regulated by dre-miR-203b-3p_L-1R+2_1SS11CT; TYRP2 was regulated by dre-miR-203B-3P_L-1R+2_1SS11CT and mmu-miR-143-5p_R+2; tyrosine 3-monooxygenase (TY3H) was regulated by ola-miR-30C_1ss21AG, dre-miR-204-5p_L+1, dre-miR-204-5p_R+2, ola-miR-16, tni-miR-16_R-1, oan-miR-16b-5p_1ss22GA, tni-miR-15a_R-1; and L-amino-acid oxidase (OXLA) was targeted by ola-miR-16, tni-miR-16_R-1, oan-miR-16b-5p_1ss22GA, tni-miR-15a_R-1, and PC-5p-43190_23 (Fig 10). Tyrosine metabolism interested us due to its indispensable role in melanin biosynthesis and because its disorder has been associated with albinism in mammals [45].

### The comparison of expression profiles between miRNA and the predicted miRNA targets

Based on the genes information derived from RNA-seq and miRNA targeted mRNAs in miRNA-seq, 133 predicted target genes were also found to be changed in albinism versus normal skin tissue (S16 Table). A series of pigmented related genes including MITF, MC1R, GCH1, XDH, BMP1, TRY, TYRP1, EDNRB, TBX19, and WNT7B were contained. Further, the comparison of expression profiles between miRNA and the predicted miRNA targets revealed that 206 mRNA-miRNA pairs changed in the same direction and 130 mRNA-miRNA pairs changed in the opposite direction (S17 Table).

## Discussion

In this study, we have identified and investigated the expression profile of mRNAs and miRNAs from albino and normally pigmented *P*. *olivaceus*. By introducing the genome sequences of *P*. *olivaceus* as a reference, 22,498 genes containing 711 new genes and 475 miRNAs containing 64 novel miRNAs were obtained. Compared with previous fish studies, this is the largest number of miRNAs identified from a single experiment [34–38]. To date, there are 20 miRNAs known in *P*. *olivaceus* based on miRbase 21.0, derived from the miRNA libraries of the metamorphosis period [37]. The high conservation of the known 411 detected miRNAs with 37 other species’ miRNAs in miRBase 21.0 suggested the conservation of miRNA sequences in the evolution process of different species [46]. On the other hand, 316 out of 411 (76.89%) miRNAs showed differences including base deletion or addition or base substitution, which also revealed the diversity of miRNAs in different species. Additionally, the identifications of 711 novel genes and 64 novel miRNAs are important supplements to *P*. *olivaceus* genome [42] and the known pool of 1044 miRNAs of fish [47].

The expression profile of mRNA and miRNAs was further investigated to gain insight into their possible roles in *P*. *olivaceus* albinism. By comparison with previous pigmentation related genes, at least 30 genes exhibiting down-regulated expression pattern were screened in differentially expressed genes of RNA-seq. As the major enzymes participated in mammals albinism [7], TYR, TYRP1, OCA2 and SLC45A2 also exhibited down regulation in albino pigmented *P*. *olivaceus* individuals. Although no differential expression of GPR143 was observed as in mammals’ albinism [48], the identification of other G-protein coupled receptor including GPR21 and GPR61 may imply their possible involvement in fish albinism. Among the overlapped genes, more than one paralogs of EDNRB, GCH1, KRT13, and TYRP1 were observed in *P*. *olivaceus*, which is consistent with the duplication of pigmentation genes in fish [49, 50].

Although a large number of miRNAs have been identified in the melanocyte biology of mammals [51], the amount of miRNA in fish pigmentation has rarely been studied [32]. The present study discovered 33 differentially expressed miRNAs in albino versus normally pigmented *P*. *olivaceus*, which suggested that miRNAs may also be involved in flatfish pigmentation. In addition, the miRNAs identified in this study have shed light on potential regulatory mechanisms of fish pigmentation deficiency. For example, miR-25 has been identified as an important regulator of pigmentation by targeting MITF in alpaca (*Lama pocos*) [21]; the homologue of miR-25 (dre-miR-25-3p) was also detected in *P*. *olivaceus*, with 1.70-fold up-regulation in albino skin. Additionally, the putative target genes of dre-miR-25-3p contains pax3b, a key transcription factor involved in melanocytes [9] and MC1R, a G protein-coupled receptor involved in regulating mammalian skin and hair colour [51].

Interaction networks between miRNAs, target genes and transcription factors are critical for an appropriate balance of gene expression in the melanocytes of mammals [52]. The target gene prediction of 33 differentially expressed miRNAs in the present study revealed 14655 putative target genes. The overlapped genes list of these targeted genes and differentially expressed genes in RNA-seq, as well as the mRNA-miRNA pairs exhibited opposite expression profiles, both provided important clues for further research. One of these putative target genes, MC1R, a G protein-coupled receptor located on the plasma membrane of melanocytes, has been reported to be involved in regulating mammalian skin and hair colour by binding to adrenocorticotropic hormone (ACTH) and melanocyte-stimulating hormone (MSH) [51]. In *P*. *olivaceus*, MC1R mRNA exhibited 2.7759 log2FoldChange down-regulation in albino skin. And it was predicted to be the target gene of 10 miRNAs, five of which showed up-regulated expression pattern. As a key transcription factor in regulating melanocytes, MITF has been predicted to be an important target gene for numerous miRNAs in mammals’ pigmentation [20–23]. Our study also suggested that 3 miRNAs including one up-regulated miRNAs targeted MITF in *P*. *olivaceus*, and its mRNA correspondingly decreased by 6.6756 log2FoldChange in albino skin. Mutations in MELPH, which is known as a downstream gene regulated by MITF, result in the dilute coat colour phenotype in dogs and cats [53–55]. In this study, MELPH was identified as a potential target of 2 miRNAs. Another target gene, TY3H, is responsible for the conversion from the amino acid L-tyrosine to L-3,4-dihydroxyphenylalanine (L-DOPA), which is the substrate for melanin synthesis [56, 57]. There have been 5 miRNAs identified that regulate TY3H in *P*. *olivaceus*.

As a phenomenon of mass depigmentation on the ocular side, albinism was also observed and investigated in other flatfish, such as turbot (*Scophthmlmus maximus*), Southern flounder (*Paralichthys lethostigma*) and Summer flounder (*Paralichthys dentatus*). Many factors, including light and nutritional factors, have been suggested to influence the occurrence of flatfish albinism [58–60]. The feeding of diets deficient in vitamin A, docosahexaenoic acid (DHA) and phospholipids will inhibit the rhodopsin production in the retina of flatfish, which will further lead to the interruption of melanin synthesis [61]. In the present study, several phospholipid-related pathways including glycosylphosphatidylinositol and glycerophospholipid metabolism were significantly enriched, which suggested an intrinsic correlation between phospholipid metabolism and abnormal pigmentation in *P*. *olivaceus*. In invertebrates and lower vertebrates, light first activates melanopsin-expressing retinal ganglion cells, which then initiate a neuro-endocrine circuit that regulates melanin dispersion/aggregation in skin melanophores through the secretion of α-melanocyte stimulating hormone (α-MSH) [62–65]. In the present study, the phototransduction pathway was significantly enriched, including the Green-sensitive opsin-4 (OPSG4), which was targeted by 2 miRNAs: dre-miR-26a-5p_L-1_1ss21CT and hhi-miR-26_R+1. Another gene, Blue-sensitive opsin (OPSB), was targeted by 4 miRNAs: dre-miR-26a-5p_L-1_1ss21CT, hhi-miR-26_R+1, xtr-miR-222_R-1, and ola-miR-106a_R+2. These results suggest the important role of the phototransduction pathway in the pigmentation of *P*. *olivaceus*.

Previous research has revealed that lysosomes share common precursors and endosomal pathways with melanosomes [66, 67], which implied that the regulation network involved in lysosomes would eventually affect melanosome biogenesis. In the present study, the observation of significant enrichment in the lysosome pathways in KEGG analysis further indicates the close relevance between lysosomes and melanosome biogenesis. The enrichment of the peroxisome proliferator-activated receptor (PPAR) signalling pathway interested us because PPARs have been shown to inhibit melanocyte growth and stimulate melanogenesis through a connection with α-MSH [67–69].

Taken together, these results provide novel insight into the albinism mechanism of *P*. *olivaceus*. The quantitative mRNA/miRNA data, network and pathway information presented here offer a solid starting point for the elucidation of detailed functions of mRNAs and miRNAs in fish albinism.

In conclusion, we constructed six cDNA libraries and six small RNA libraries of skin tissues from three albinos and three normally pigmented individuals of *P*. *olivaceus*. As a result of sequencing and data treatment, a total of 22,498 genes including 711 new genes, and a total of 475 miRNAs including 64 novel miRNAs were both identified. A total of 235 genes and 33 miRNAs exhibited differential expression patterns in albino versus normally pigmented *P*. *olivaceus*. The differentially expressed genes were significantly enriched in Folate biosynthesis, Melanogenesis, and Neuroactive ligand-receptor interaction pathways. The subsequent *in silico* analysis predicted 14655 putative target genes, of which a variety of genes related to melanin metabolism interested us, including MITF, TYR, MC1R, TYRP1, TYRP2, TY3H, and MELPH. The GO and KEGG analyses revealed that these target genes were significantly enriched in 254 GO terms and 103 pathways. The network mapping of albinism-related miRNAs and their target genes will serve as substantial clues for future research on the mechanism of pigmentation deficiency in *P*. *olivaceus*.

## Supporting information

S1 Fig Error rate distribution along reads in six libraries of RNA-seq. (PDF)

S2 Fig The pearson correlation between samples in RNA-seq. (PDF)

S3 Fig The FastQC analysis before and after trimming in six small RNA libraries. (PDF)

S4 Fig The correlation analysis in six small RNA libraries. (PDF)

S1 Table Species priority from vertebrate subdivision mapped to miRBase 21.0. (PDF)

S2 Table Primers used for qRT-PCR of genes. (PDF)

S3 Table Primers used for qRT-PCR of microRNAs. (PDF)

S4 Table The summary information of RNA-seq reads. (PDF)

S5 Table The differentially expressed genes between PO_alb and PO_con (p≤0.05). (PDF)

S6 Table The GO term enrichment for differentially expressed genes in RNA-seq (p≤0.05). (PDF)

S7 Table The KEGG pathway enrichment for differentially expressed genes in RNA-seq (p≤0.05). (PDF)

S8 Table Overview of reads from raw data to cleaned sequences in miRNA-seq. (PDF)

S9 Table 31 unique miRNAs mapping to Japanese flounder miRNA/pre-miRNAs in miRbase and the pre-miRNAs further map to genome & EST. (PDF)

S10 Table 240 unique miRNAs mapping to selected species (except for Japanese flounder) miRNAs/pre-miRNAs in miRbase and the pre-miRNAs further mapping to the genome & EST. (PDF)

S11 Table 75 unique miRNAs mapping to selected species (except for Japanese flounder) miRNAs/pre-miRNAs in miRbase, while only miRNA not pre-miRNAs mapping to the genome. (PDF)

S12 Table 65 unique miRNAs mapping to selected species (except for Japanese flounder) miRNAs/pre-miRNAs in miRbase, but both pre-miRNA and miRNA do not map to the genome. (PDF)

S13 Table 64 novel unique miRNAs which do not map to selected pre-miRNAs in miRbase, but the reads map to genome & the extended genome sequences from genome may form hairpins. (PDF)

S14 Table The significant enrichment of GO terms for target genes of miRNAs (p≤0.001). (PDF)

S15 Table The significant enrichment of KEGG pathways for target genes of miRNAs (p≤0.001). (PDF)

S16 Table The overlapped genes between differentially expressed genes in RNA-seq and miRNAs’ predicted target genes in miRNA-seq. (PDF)

S17 Table The comparison of expression profiles between miRNAs and the predicted mRNA targets in PO_alb versus PO_con. (PDF)
