# Supplementary figures and images for: The integrated analysis of RNA-seq and microRNA-seq depicts miRNA-mRNA networks involved in Japanese flounder (Paralichthys olivaceus) albinism
Source: PLoS One. 2017 Aug 4;12(8):e0181761. doi: 10.1371/journal.pone.0181761 (PMC5544202; doi:10.1371/journal.pone.0181761)

S1 Fig. Error rate distribution along reads in six libraries of RNA-seq.

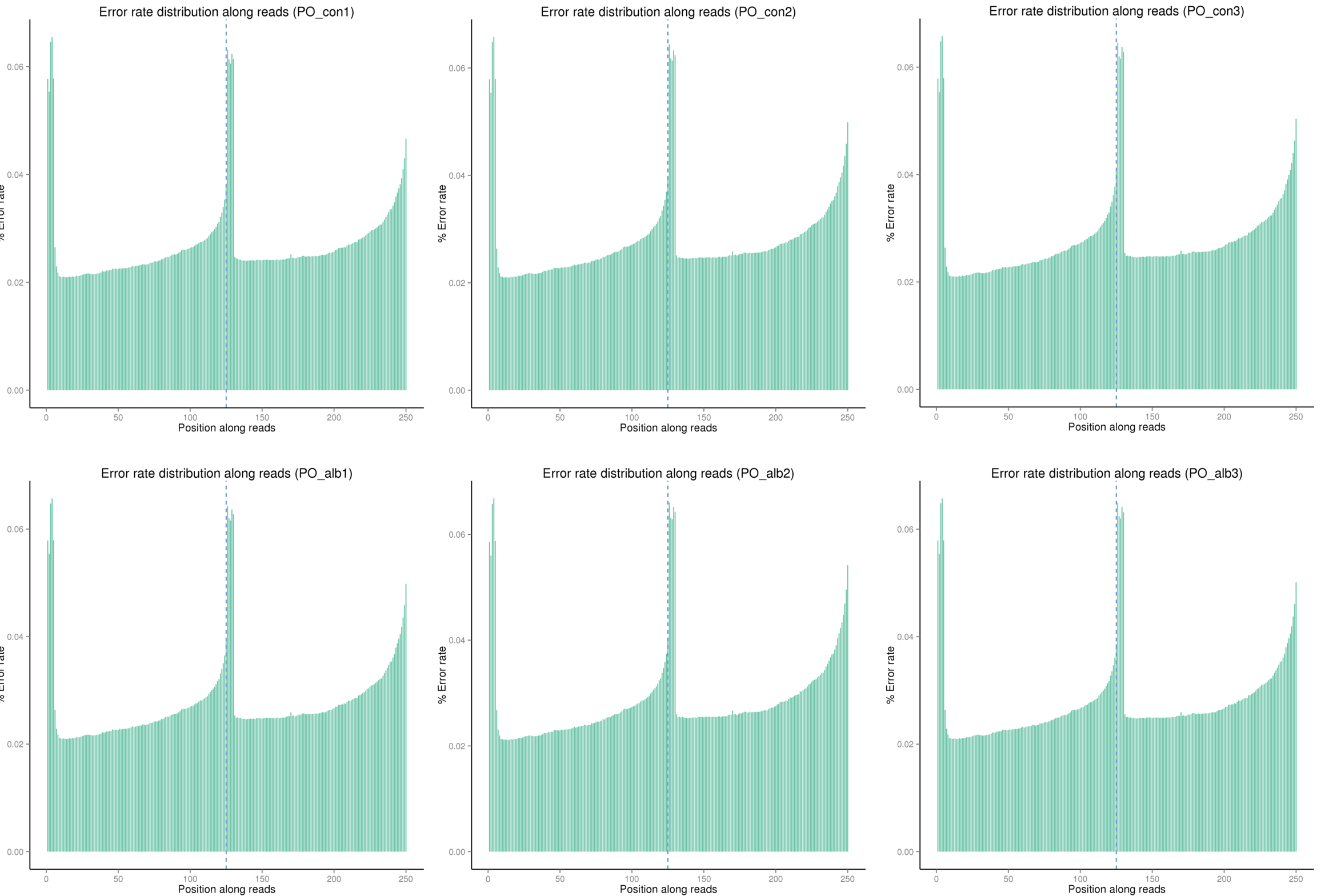

Supplement: S1 Fig — (PDF) [file pone.0181761.s001.pdf]

S2 Fig. The pearson correlation between samples in RNA-seq.

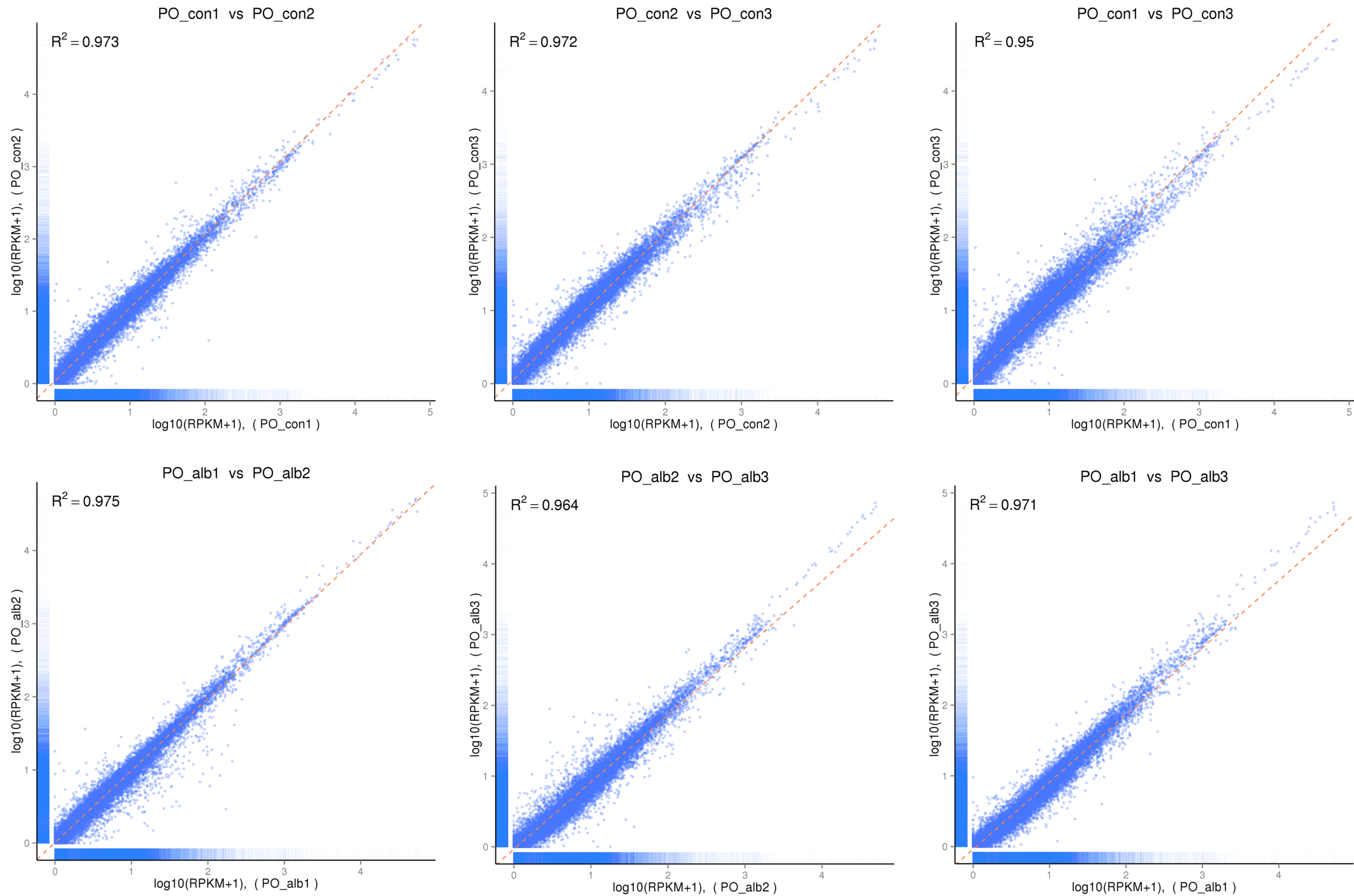

Supplement: S2 Fig — (PDF) [file pone.0181761.s002.pdf]

S3 Fig. The FastQC analysis before and after trimming in six small RNA libraries.

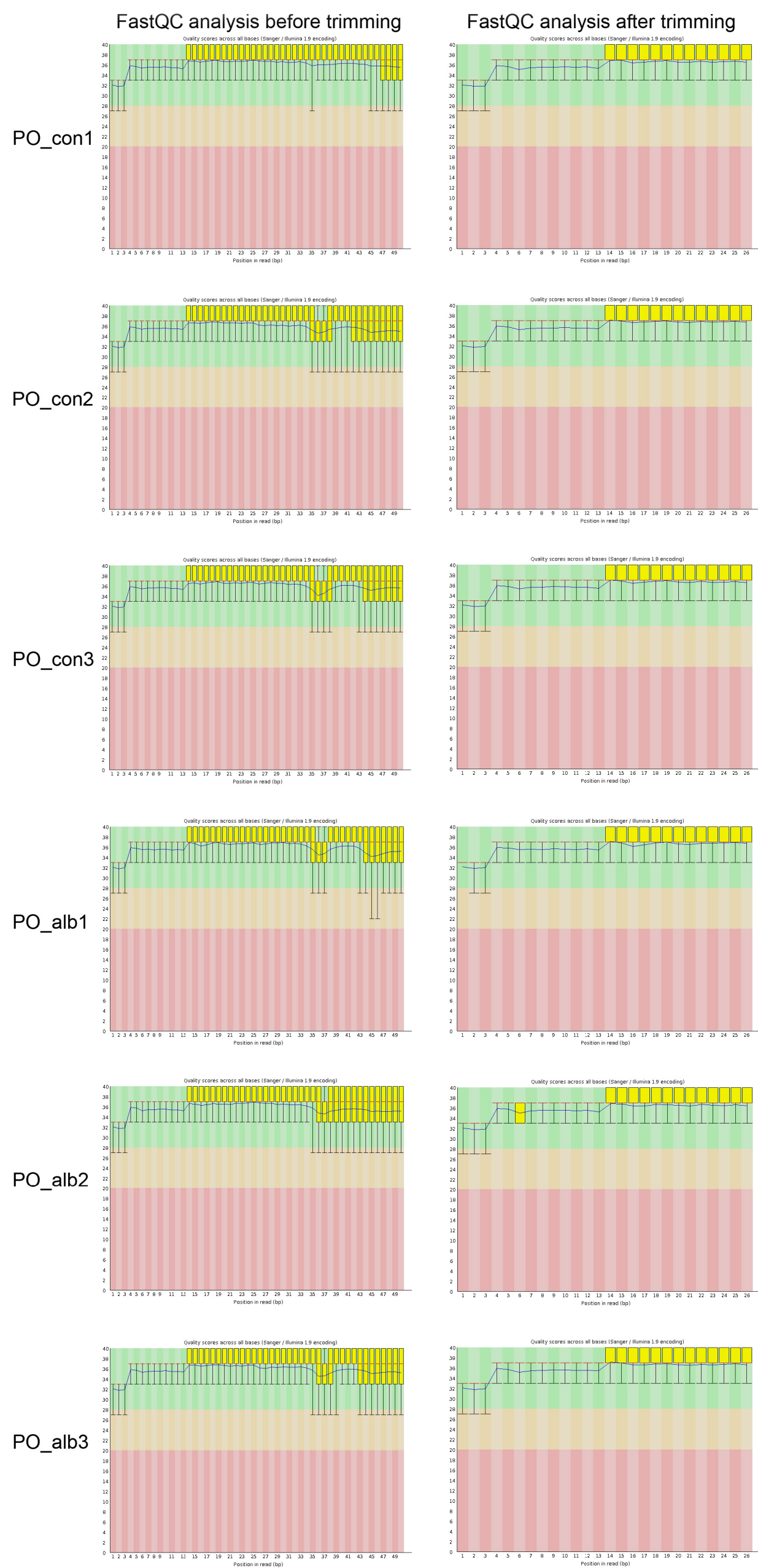

Supplement: S3 Fig — (PDF) [file pone.0181761.s003.pdf]

S4 Fig. The correlation analysis in six small RNA libraries.

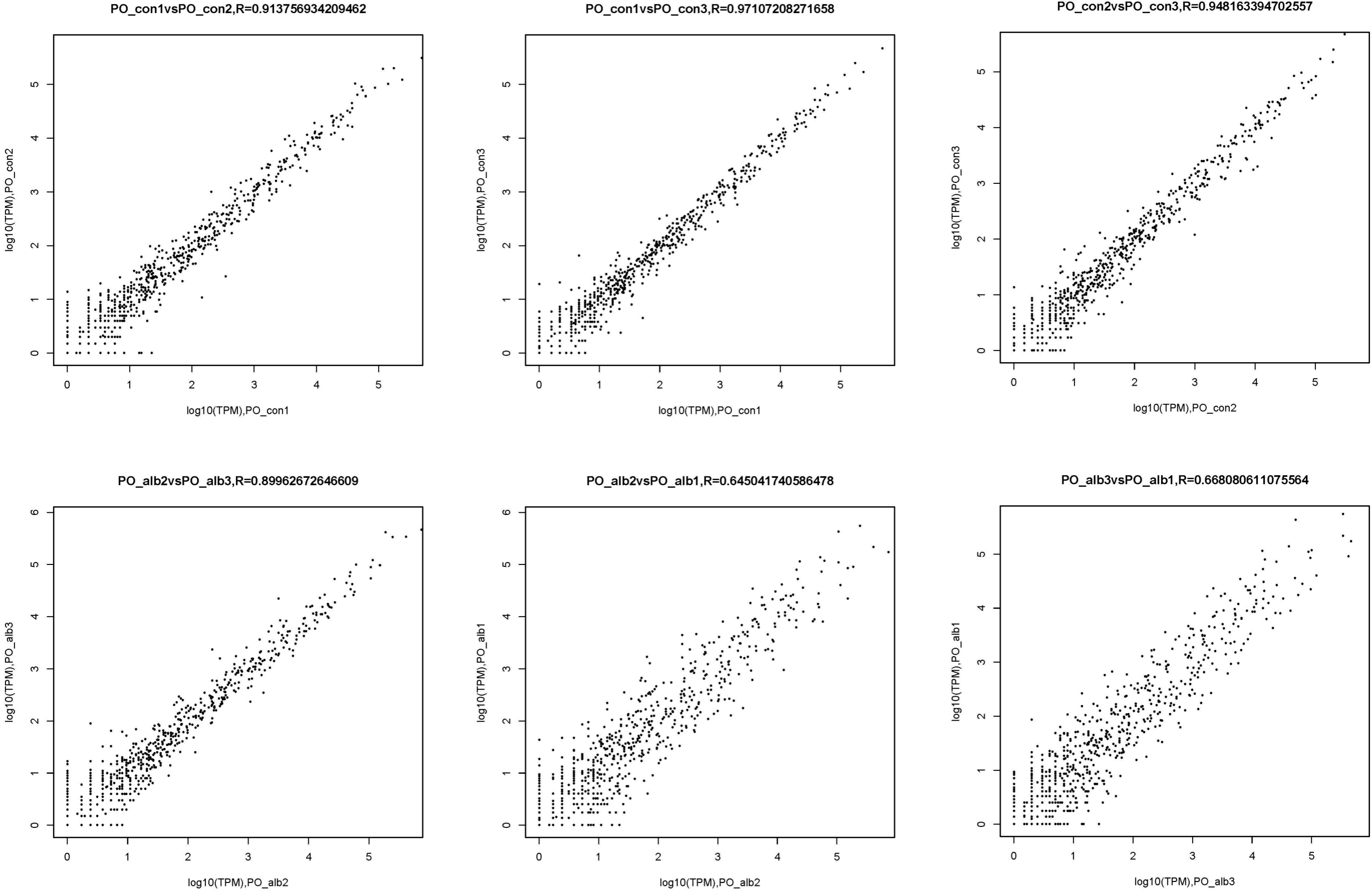

Supplement: S4 Fig — (PDF) [file pone.0181761.s004.pdf]
