# Supplementary material for: The integrated analysis of RNA-seq and microRNA-seq depicts miRNA-mRNA networks involved in Japanese flounder (Paralichthys olivaceus) albinism
Source: PLoS One. 2017 Aug 4;12(8):e0181761. doi: 10.1371/journal.pone.0181761 (PMC5544202; doi:10.1371/journal.pone.0181761)
Supplement: S1 Table — (PDF) [file pone.0181761.s005.pdf]

S1 Table. Species priority from vertebrate subdivision mapped to miRBase 21.0.

Class	Organism	Common Name	3 letter name
Pisces	Paralichthys olivaceus	Olive flounder	pol
Pisces	Danio rerio	Zebrafish	dre
Pisces	Ictalurus punctatus	channel catfish	ipu
Pisces	Oryzias latipes	Japanese killifish	ola
Pisces	Cyprinus carpio	Common carp	ccr
Pisces	Tetraodon nigroviridis	Tetraodon Pufferfish	tni
Pisces	Fugu rubripes	Fugu Pufferfish	fru
Pisces	Hippoglossus hippoglossus	Atlantic halibut	hhi
Mammalia	Homo sapiens	Human	hsa
Mammalia	Mus musculus	Mouse	mmu
Mammalia	Bos taurus	Cow	bta
Mammalia	Pongo pygmaeus	Orangutan	ppy
Mammalia	Pan troglodytes	Common Chimpanzee	ptr
Mammalia	Macaca mulatta	Rhesus Monkey	mml
Mammalia	Monodelphis domestica	Gray Short-tailed Opossum	mdo
Mammalia	Rattus norvegicus	Rat	rno
Mammalia	Ornithorhynchus anatinus	Platypus	oan
Mammalia	Equus caballus	Horse	eca
Mammalia	Canis familiaris	Dog	cfa
Mammalia	Gorilla gorilla	Gorilla	ggo
Mammalia	Sus scrofa	Pig	ssc
Mammalia	Cricetulus griseus	Chinese Hamster	cgr
Mammalia	Ovis aries	Sheep	oar
Mammalia	Pan paniscus	Pygmy Chimpanzee	ppa
Mammalia	Macaca nemestrina	Pig-tailed macaque	mne
Mammalia	Sarcophilus harrisii	Tasmanian devil	sha
Mammalia	Ateles geoffroyi	Spider monkey	age
Mammalia	Lagothrix lagotricha	Woolly monkey	lla
Mammalia	Saguinus labiatus	White-lipped Tamarin	sla
Mammalia	Artibeus jamaicensis	Mexican fruit bat	aja
Mammalia	Lemur catta	Ring-tailed Lemur	lca
Mammalia	Pygathrix bieti	Black snub-nosed monkey	pbi
Mammalia	Symphalangus syndactylus	Siamang	ssy
Mammalia	Macropus eugenii	Tammar wallaby	meu
Aves	Gallus gallus	Chicken	gga
Aves	Taeniopygia guttata	Zebra Finch	tgu
Amphibia	Xenopus tropicalis	Pipid Frog	xtr
Amphibia	Xenopus laevis	Frog	xla
Agnathostomata	Petromyzon marinus	Petromyzon marinus	pma
	Anolis carolinensis	Carolina anole	aca
