# Supplementary material for: The integrated analysis of RNA-seq and microRNA-seq depicts miRNA-mRNA networks involved in Japanese flounder (Paralichthys olivaceus) albinism
Source: PLoS One. 2017 Aug 4;12(8):e0181761. doi: 10.1371/journal.pone.0181761 (PMC5544202; doi:10.1371/journal.pone.0181761)
Supplement: S2 Table — (PDF) [file pone.0181761.s006.pdf]

S2 Table. Primers used for qRT-PCR of genes.

primers	sequences (5'-3')
fabp2-F	CCGCTCACGACAACCTCAAGAT
fabp2-R	ATCTGCAAGGCTGTACTCGAATGT
glrk-F	CTGGTGGCTGTTTGCTCTAATGC
glrk-R	TCCTGGTTGGCGAGGTCTTCA
hspa8-F	ACAAGAACCAGACAGCAGAGAAGG
hsp8-R	AACCACCAGGCATCCCACCA
gch1-F	ATGCGAGGCGTCCAGAAGATG
gch1-R	TGCTGTGAAAGGGTCAAGAACTCT
novel00096-F	GCTGCTACTGAGGCTAAGAGACG
novle00096-R	GGAACCTGTGCTGATGTCATTGTG
pmel-F	GCGAGTGCCAGAGTCAGTGTAA
pmel-R	CAACAGACGACACAGGCGAGAA
tmem130-F	TCTCCTTCATCGCAGTCATCGC
tmem130-R	GAATCGCACTCTGGTCGTCTGA
tyr-F	GAGTTCCTGACGCCATACCTCG
tyr-R	CCACTTCCTCCTCGCAACAACA
tyrp1-F	ACAACCTGGCACATCTCTTCCT
tyrp1-R	ACTCTGTCTCCTCAACCATTCATCA
wnt7b-F	GCGGTTCAAGTGGAGCCTGT
wnt7b-R	CGTGTCCTCCTCGCAGTAGTTG
