# Supplementary material for: The integrated analysis of RNA-seq and microRNA-seq depicts miRNA-mRNA networks involved in Japanese flounder (Paralichthys olivaceus) albinism
Source: PLoS One. 2017 Aug 4;12(8):e0181761. doi: 10.1371/journal.pone.0181761 (PMC5544202; doi:10.1371/journal.pone.0181761)
Supplement: S3 Table — (PDF) [file pone.0181761.s007.pdf]

S3 Table. Primers used for qRT-PCR of microRNAs.

miRNAs	primer sequences (5'-3')
tni-miR-16_R-1	GGCTAGCAGCACGTAAATATTGGA
hhi-miR-26_R+1	GGCTTCAAGTAATCCAGGATAGGCT
ola-miR-16	GGCTAGCAGCACGTAAATATTGGC
dre-miR-18b-5p_1ss11TC	GCCTAAGGTGCATCTAGTGCAGATA
ola-miR-106a_R+2	GCTAAAGTGCTTACAGTGCAGGTAG
dre-miR-26a-5p_R-1_1ss21CT	CGCGTTCAAGTAATCCAGGATAGGT
dre-miR-204-5p_L+1	CCTTTCCCTTTGTCATCCTATGCCT
pol-miR-199a-5p_R+1	CCCCAGTGTTCAGACTACCTGTTC
mmu-miR-143-5p_R+2	GCAGTGCTGCATCTCTGGTC
PC-5p-59593_11	GGACCCGTAGATCCGAACTTGT
5s rRNA	GCTTACGGCCATACCACCCT
