# Supplementary material for: The integrated analysis of RNA-seq and microRNA-seq depicts miRNA-mRNA networks involved in Japanese flounder (Paralichthys olivaceus) albinism
Source: PLoS One. 2017 Aug 4;12(8):e0181761. doi: 10.1371/journal.pone.0181761 (PMC5544202; doi:10.1371/journal.pone.0181761)
Supplement: S4 Table — (PDF) [file pone.0181761.s008.pdf]

S4 Table. The summary information of RNA-seq reads.

Sample name	Raw reads	Clean reads	Clean bases	Error rate(%)	Q20(%)	Q30(%)	GC content(%)
PO_con1_1	35916787	34279317	4.28G	0.03	96.33	92.5	51.39
PO_con1_2	35916787	34279317	4.28G	0.04	95.03	90.52	51.4
PO_con2_1	32490818	31723503	3.97G	0.03	96.12	92.18	50.37
PO_con2_2	32490818	31723503	3.97G	0.04	94.62	89.86	50.38
PO_con3_1	31290871	30636852	3.83G	0.03	96.12	92.14	50.49
PO_con3_2	31290871	30636852	3.83G	0.04	94.57	89.79	50.5
PO_alb1_1	31563378	30708267	3.84G	0.03	96.17	92.26	50.47
PO_alb1_2	31563378	30708267	3.84G	0.04	94.58	89.8	50.48
PO_alb2_1	33093738	31968556	4G	0.03	96.04	92.01	50.73
PO_alb2_2	33093738	31968556	4G	0.04	94.17	89.11	50.74
PO_alb3_1	31370982	30617716	3.83G	0.03	96.23	92.34	51
PO_alb3_2	31370982	30617716	3.83G	0.04	94.54	89.74	51
