# Supplementary material for: The integrated analysis of RNA-seq and microRNA-seq depicts miRNA-mRNA networks involved in Japanese flounder (Paralichthys olivaceus) albinism
Source: PLoS One. 2017 Aug 4;12(8):e0181761. doi: 10.1371/journal.pone.0181761 (PMC5544202; doi:10.1371/journal.pone.0181761)
Supplement: S5 Table — (PDF) [file pone.0181761.s009.pdf]

**S5 Table. The differentially expressed genes between PO_alb and PO_con (p≤0.05).**

| Gene_id | readcount PO_alb | readcount PO_con | log2FC | pval | padj | gene description |
|---|---|---|---|---|---|---|
| GS_001665 | 0 | 7.951319 | Inf | 0.00023 | 0.02402 | DLGP2_MOUSE Disks large-associated protein 2 Dlgap2 |
| GS_005670 | 0 | 9.378123 | Inf | 4.83E-05 | 0.0062 | EZRI_HUMAN Ezrin EZR |
| GS_014569 | 0 | 9.507661 | Inf | 4.57E-05 | 0.00591 | HMOX2_RAT Heme oxygenase 2 Hmox2 |
| GS_001664 | 0 | 9.712871 | Inf | 3.47E-05 | 0.00462 | CYTSA_CHICK Cytospin-A SPECC1L |
| GS_019678 | 0 | 11.42221 | Inf | 6.67E-06 | 0.00105 | NHRF3_RAT Na(+)/H(+) exchange regulatory cofactor NHE-RF3 Pdzk1 |
| GS_010503 | 0 | 11.20372 | Inf | 6.06E-06 | 0.00098 | |
| GS_003950 | 0 | 36.61633 | Inf | 3.09E-06 | 0.00054 | S13A5_HUMAN Solute carrier family 13 member 5 SLC13A5 |
| GS_019288 | 0 | 25.51627 | Inf | 1.04E-11 | 4.09E-09 | S23A1_MOUSE Solute carrier family 23 member 1 Slc23a1 |
| GS_011397 | 0 | 27.60594 | Inf | 1.39E-12 | 6.14E-10 | MC5R_PANTR Melanocortin receptor 5 MC5R |
| GS_005903 | 0 | 284.3595 | Inf | 2.06E-18 | 1.65E-15 | GCH1_HUMAN GTP cyclohydrolase 1 GCH1 |
| GS_000806 | 0 | 101.0247 | Inf | 3.59E-21 | 3.25E-18 | |
| GS_000805 | 0 | 55.58731 | Inf | 5.38E-22 | 5.60E-19 | |
| GS_000807 | 0.432359 | 521.7506 | -10.237 | 1.14E-77 | 2.37E-73 | |
| GS_011014 | 0.759496 | 834.1087 | -10.101 | 1.63E-11 | 6.28E-09 | |
| GS_013312 | 1.058333 | 500.5233 | -8.8855 | 2.01E-13 | 9.51E-11 | DFP_MANSE Putative defense protein Hdd11-like |
| GS_011013 | 2.722316 | 1179.75 | -8.7594 | 4.27E-16 | 2.69E-13 | HEBP2_HUMAN Heme-binding protein 2 HEBP2 |
| GS_020687 | 0.96726 | 381.9457 | -8.6252 | 1.95E-16 | 1.31E-13 | TM130_HUMAN Transmembrane protein 130 TMEM130 |
| GS_008748 | 2.159115 | 803.6782 | -8.54 | 7.31E-23 | 8.45E-20 | GCH1_HUMAN GTP cyclohydrolase 1 GCH1 |
| GS_005902 | 3.114906 | 1056.709 | -8.4062 | 1.71E-17 | 1.18E-14 | GCH1_RAT GTP cyclohydrolase 1 Gch1 |
| Novel00024 | 15.73442 | 3120.104 | -7.6315 | 5.00E-12 | 2.04E-09 | |
| GS_020150 | 0.640124 | 122.8345 | -7.5841 | 1.37E-22 | 1.50E-19 | KINH_RAT Kinesin-1 heavy chain Kif5b |
| Novel00023 | 1.308547 | 240.4501 | -7.5216 | 4.63E-50 | 4.82E-46 | |
| GS_013228 | 1.072483 | 187.5607 | -7.4503 | 1.08E-15 | 6.61E-13 | GCH1_HUMAN GTP cyclohydrolase 1 GCH1 |
| GS_007330 | 0.745346 | 111.0871 | -7.2196 | 6.15E-33 | 1.07E-29 | NCKX3_HUMAN Sodium/potassium/calcium exchanger 3 SLC24A3 |
| Novel00307 | 3.126376 | 373.3751 | -6.9 | 1.71E-13 | 8.27E-11 | |
| GS_006908 | 1.920371 | 219.4224 | -6.8362 | 3.14E-13 | 1.45E-10 | PLPL2_HUMAN Patatin-like phospholipase domain-containing protein 2 PNPLA2 |
| GS_008590 | 5.388033 | 562.8665 | -6.7069 | 4.05E-09 | 1.16E-06 | FICA_AGKHB Salmorin subunit A |
| GS_002043 | 0.327137 | 33.44125 | -6.6756 | 5.47E-09 | 1.54E-06 | MITF_MOUSE Microphthalmia-associated transcription factor Mitf |
| GS_003739 | 4.588768 | 456.8645 | -6.6375 | 3.12E-12 | 1.35E-09 | BDH_MOUSE D-beta-hydroxybutyrate dehydrogenase, mitochondrial Bdh1 |
| GS_001154 | 2.799239 | 252.6781 | -6.4961 | 1.06E-36 | 3.14E-33 | KINH_HUMAN Kinesin-1 heavy chain KIF5B |
| GS_002818 | 0.654274 | 58.31492 | -6.4778 | 7.91E-09 | 2.14E-06 | S6A11_RAT Sodium- and chloride-dependent GABA transporter 3 Slc6a11 |
| GS_018011 | 4.565765 | 402.1071 | -6.4606 | 1.97E-28 | 2.74E-25 | GTR11_HUMAN Solute carrier family 2, facilitated glucose transporter member 11 SLC2A11 |

| | | | | | | |
|---|---|---|---|---|---|---|
| GS_007124 | 3.035303 | 266.3156 | -6.4552 | 7.74E-22 | 7.67E-19 | SPRE_XENLA Sepiapterin reductase spr |
| GS_007057 | 0.327137 | 28.5148 | -6.4457 | 3.32E-10 | 1.11E-07 | S41A2_CHICK Solute carrier family 41 member 2 SLC41A2 |
| GS_020470 | 9.043201 | 750.6705 | -6.3752 | 8.34E-18 | 5.99E-15 | TT39B_XENTR Tetratricopeptide repeat protein 39B ttc39b |
| GS_005953 | 0.432359 | 33.98929 | -6.2967 | 1.02E-13 | 5.03E-11 | PHOS_MOUSE Phosducin Pdc |
| Novel00653 | 0.864718 | 59.51106 | -6.1048 | 3.38E-14 | 1.76E-11 | |
| GS_007052 | 16.76795 | 988.6148 | -5.8816 | 7.78E-14 | 3.95E-11 | DYR_SHV21 Viral dihydrofolate reductase DHFR |
| GS_003590 | 3.976944 | 210.2207 | -5.7241 | 1.28E-06 | 0.00025 | PMEL_CHICK Melanocyte protein PMEL PMEL |
| GS_015726 | 4.542825 | 236.5917 | -5.7027 | 3.39E-43 | 2.35E-39 | RFT2_SALSA Riboflavin transporter 2 r rft2 |
| GS_001157 | 0.640124 | 29.92103 | -5.5467 | 2.88E-11 | 1.05E-08 | ABP1_RAT Amiloride-sensitive amine oxidase [copper-containing] Abp1 |
| GS_005047 | 4.204218 | 193.1395 | -5.5217 | 4.05E-15 | 2.28E-12 | PLIN2_BOVIN Perilipin-2 PLIN2 |
| GS_014478 | 0.327137 | 12.77179 | -5.2869 | 9.75E-06 | 0.00152 | TBX19_CANFA T-box transcription factor TBX19 TBX19 |
| GS_001546 | 3.976944 | 137.5623 | -5.1123 | 0.00022 | 0.02347 | OSTCN_SPAAU Osteocalcin bglap |
| GS_020975 | 1.610064 | 50.89513 | -4.9823 | 1.31E-15 | 7.81E-13 | TYRO_ORYLA Tyrosinase tyr |
| GS_009038 | 9.327824 | 289.2796 | -4.9548 | 2.79E-40 | 1.16E-36 | TYRP1_HUMAN 5,6-dihydroxyindole-2-carboxylic acid oxidase TYRP1 |
| GS_010733 | 4.813363 | 145.2044 | -4.9149 | 1.45E-30 | 2.15E-27 | RDH11_MOUSE Retinol dehydrogenase 11 Rdh11 |
| GS_007143 | 7.979508 | 240.0816 | -4.9111 | 1.38E-37 | 4.80E-34 | ADA2B_DANRE Alpha-2B adrenergic receptor adra2b |
| GS_016824 | 0.312987 | 9.342832 | -4.8997 | 0.00029 | 0.02924 | KACB_RAT Ig kappa chain C region, B allele |
| GS_012029 | 9.905239 | 292.5663 | -4.8844 | 3.55E-33 | 6.72E-30 | TYRP1_BOVIN 5,6-dihydroxyindole-2-carboxylic acid oxidase TYRP1 |
| GS_000169 | 3.766499 | 106.9919 | -4.8281 | 6.44E-13 | 2.91E-10 | RGS8_DANRE Regulator of G-protein signaling 8 rgs8 |
| GS_004712 | 12.51965 | 345.3098 | -4.7856 | 1.18E-41 | 6.12E-38 | F123A_HUMAN Protein FAM123A FAM123A |
| GS_017984 | 0.312987 | 8.626111 | -4.7845 | 0.00055 | 0.04891 | LIX1_CHICK Protein limb expression 1 LIX1 |
| GS_000623.[illegible] | 3.083926 | 78.97306 | -4.6785 | 4.28E-07 | 9.10E-05 | HVCM_HETFR Ig heavy chain C region, membrane-bound form |
| GS_012545 | 5.823886 | 144.1497 | -4.6294 | 2.74E-26 | 3.57E-23 | K1C18_PROAT Keratin, type I cytoskeletal 18 krt18 |
| GS_003949 | 7.12283 | 163.5666 | -4.5213 | 7.24E-09 | 2.01E-06 | S47A1_DANRE Multidrug and toxin extrusion protein 1 slc47a1 |
| GS_017127 | 8.138649 | 176.0336 | -4.4349 | 2.22E-14 | 1.19E-11 | CCD18_HUMAN Coiled-coil domain-containing protein 18 CCDC18 |
| GS_014384 | 4.306761 | 92.26403 | -4.4211 | 3.81E-16 | 2.48E-13 | RETST_DANRE Putative all-trans-retinol 13,14-reductase retsat |
| GS_019504 | 1.817829 | 38.3572 | -4.3992 | 3.53E-12 | 1.50E-09 | TRPC2_RAT Short transient receptor potential channel 2 Trpc2 |
| GS_007375 | 4.213008 | 79.84825 | -4.2443 | 8.11E-19 | 7.04E-16 | S22A7_BOVIN Solute carrier family 22 member 7 SLC22A7 |
| GS_000869 | 14.69292 | 277.1858 | -4.2377 | 6.07E-34 | 1.26E-30 | MAR1_HUMAN Melanoma antigen recognized by T-cells 1 MLANA |
| GS_011724 | 17.27374 | 314.8135 | -4.1878 | 1.19E-34 | 2.76E-31 | KAP1_HUMAN cAMP-dependent protein kinase type I-beta regulatory subunit PRKAR1B |
| GS_013459 | 2.932761 | 51.79571 | -4.1425 | 2.95E-05 | 0.00401 | SIX3_CHICK Homeobox protein SIX3 SIX3 |
| GS_014376 | 1.803679 | 31.72671 | -4.1367 | 8.52E-08 | 2.06E-05 | S6A13_MOUSE Sodium- and chloride-dependent GABA transporter 2 Slc6a13 |
| GS_013990 | 1.058333 | 17.52303 | -4.0494 | 2.63E-06 | 0.00047 | FSTL5_HUMAN Follistatin-related protein 5 FSTL5 |
| GS_012501 | 1.058333 | 16.47145 | -3.9601 | 5.86E-06 | 0.00095 | BMP1_HUMAN Bone morphogenetic protein 1 BMP1 |
| GS_017193 | 1.39962 | 21.25818 | -3.9249 | 1.69E-06 | 0.00031 | P_PIG P protein Oca2 |
| GS_000839 | 30.67226 | 448.7595 | -3.8709 | 9.72E-35 | 2.53E-31 | VIME_ONCMY Vimentin vim |
| GS_008733 | 5.271341 | 68.7828 | -3.7058 | 3.55E-15 | 2.05E-12 | |

| | | | | | | |
|---|---|---|---|---|---|---|
| GS_014490 | 7.166093 | 91.58415 | -3.6758 | 5.59E-18 | 4.31E-15 | XDH_HUMAN Xanthine dehydrogenase/oxidase XDH |
| GS_020483 | 44.4031 | 537.9631 | -3.5988 | 2.19E-32 | 3.50E-29 | LX15B_RAT Arachidonate 15-lipoxygenase B Alox15b |
| GS_010033 | 1.504842 | 17.47724 | -3.5378 | 1.10E-05 | 0.00169 | ARL4C_MOUSE ADP-ribosylation factor-like protein 4C Arl4c |
| GS_020215 | 4.184709 | 44.96788 | -3.4257 | 2.79E-06 | 0.00049 | PO2F3_RAT POU domain, class 2, transcription factor 3 Pou2f3 |
| GS_006323 | 11.31982 | 120.5895 | -3.4132 | 1.49E-18 | 1.24E-15 | DLRB2_BOVIN Dynein light chain roadblock-type 2 DYNLRB2 |
| GS_006432 | 22.31251 | 234.4571 | -3.3934 | 8.17E-18 | 5.99E-15 | ADCY5_HUMAN Adenylate cyclase type 5 ADCY5 |
| GS_019698 | 2.770939 | 28.61049 | -3.3681 | 1.14E-07 | 2.71E-05 | MAP6_CHICK Microtubule-associated protein 6 homolog MAP6 |
| GS_018288 | 2.35273 | 23.23004 | -3.3036 | 1.58E-06 | 0.0003 | MCHR2_MACFA Melanin-concentrating hormone receptor 2 MCHR2 |
| GS_019585 | 2.665717 | 25.8945 | -3.28 | 7.92E-05 | 0.00942 | ENDD1_HUMAN Endonuclease domain-containing 1 protein ENDOD1 |
| GS_020808 | 7.997151 | 76.89337 | -3.2653 | 2.76E-05 | 0.00384 | DCE1_HUMAN Glutamate decarboxylase 1 GAD1 |
| GS_002681 | 1.951351 | 18.31033 | -3.2301 | 1.22E-05 | 0.00185 | DRD5L_TAKRU D(5)-like dopamine receptor dl |
| Novel00277 | 6.876046 | 62.79535 | -3.191 | 5.63E-05 | 0.00706 | |
| GS_020430 | 1.831979 | 16.65331 | -3.1843 | 3.54E-05 | 0.00467 | Y8948_DICDI Putative methyltransferase DDB_G0268948 DDB_G0268948 |
| GS_014685 | 5.777943 | 52.01708 | -3.1704 | 1.10E-10 | 3.82E-08 | GTR9_HUMAN Solute carrier family 2, facilitated glucose transporter member 9 SLC2A9 |
| GS_015091 | 3.098076 | 26.82902 | -3.1143 | 5.87E-05 | 0.00727 | PI3R6_MOUSE Phosphoinositide 3-kinase regulatory subunit 6 Pik3r6 |
| GS_002625 | 10.80973 | 92.73437 | -3.1008 | 0.00035 | 0.03402 | IL1B_ONCMY Interleukin-1 beta il1b |
| GS_014684 | 4.121936 | 32.90253 | -2.9968 | 1.53E-07 | 3.49E-05 | GTR9_PONAB Solute carrier family 2, facilitated glucose transporter member 9 SLC2A9 |
| Novel00559 | 14.39757 | 108.2886 | -2.911 | 2.23E-14 | 1.19E-11 | |
| GS_012394 | 16.78746 | 124.5129 | -2.8908 | 6.45E-08 | 1.58E-05 | TRY3_CHICK Trypsin II-P29 |
| GS_000962 | 5.436657 | 38.26595 | -2.8153 | 1.36E-07 | 3.19E-05 | SPIR1_DANRE Protein spire homolog 1 spire1 |
| GS_013701 | 3.485306 | 24.51692 | -2.8144 | 1.56E-05 | 0.00228 | RASFA_HUMAN Ras association domain-containing protein 10 RASSF10 |
| GS_007378 | 3.988414 | 27.31687 | -2.7759 | 2.22E-05 | 0.00317 | MSHR_CHICK Melanocyte-stimulating hormone receptor MC1R |
| GS_000153 | 4.392473 | 27.54853 | -2.6489 | 1.03E-05 | 0.00159 | NCAM2_HUMAN Neural cell adhesion molecule 2 NCAM2 |
| GS_016413 | 3.411063 | 21.09315 | -2.6285 | 6.74E-05 | 0.0082 | CA106_HUMAN Uncharacterized protein C1orf106 C1orf106 |
| GS_012916 | 5.837222 | 35.13345 | -2.5895 | 4.89E-07 | 0.0001 | S45A2_MOUSE Membrane-associated transporter protein Slc45a2 |
| GS_000622 | 18.59026 | 108.0145 | -2.5386 | 5.74E-12 | 2.30E-09 | MUCM_ICTPU Ig mu chain C region membrane-bound form |
| GS_000397 | 6.853106 | 39.47936 | -2.5263 | 2.70E-07 | 5.99E-05 | CNTN4_MOUSE Contactin-4 Cntn4 |
| GS_002614 | 12.57445 | 72.26562 | -2.5228 | 5.32E-10 | 1.73E-07 | GPR21_HUMAN Probable G-protein coupled receptor 21 GPR21 |
| GS_014714 | 22.41773 | 128.6525 | -2.5208 | 3.47E-06 | 0.00059 | XYLT1_PANTR Xylosyltransferase 1 XYLT1 |
| GS_016285 | 239.1776 | 1363.875 | -2.5116 | 1.54E-21 | 1.46E-18 | S10I_ICTPU Ictacalcin |
| GS_021169 | 2.890312 | 16.36028 | -2.5009 | 0.00046 | 0.0425 | NSG2_HUMAN Neuron-specific protein family member 2 NSG2 |
| GS_016151 | 3.259898 | 18.28687 | -2.4879 | 0.00024 | 0.02501 | |
| Novel00658 | 4.71961 | 26.05644 | -2.4649 | 3.87E-05 | 0.00503 | |
| GS_005871 | 4.170559 | 22.57227 | -2.4362 | 7.26E-05 | 0.00868 | REP15_HUMAN Rab15 effector protein REP15 |
| GS_018934 | 3.368614 | 18.11021 | -2.4266 | 0.00046 | 0.0429 | RGR_HUMAN RPE-retinal G protein-coupled receptor RGR |
| GS_017738 | 7.845986 | 40.82365 | -2.3794 | 1.04E-06 | 0.0002 | NPDC1_HUMAN Neural proliferation differentiation and control protein 1 NPDC1 |
| Novel00253 | 4.941525 | 25.54493 | -2.37 | 8.03E-05 | 0.0095 | |

| | | | | | | |
|---|---|---|---|---|---|---|
| GS_006767 | 25.88451 | 128.3609 | -2.31 | 2.86E-11 | 1.05E-08 | ALK_MOUSE ALK tyrosine kinase receptor Alk |
| GS_007760 | 20.2313 | 96.4254 | -2.2528 | 0.00027 | 0.02735 | CP26C_HUMAN Cytochrome P450 26C1 CYP26C1 |
| GS_013111 | 6.878725 | 32.47642 | -2.2392 | 2.80E-05 | 0.00385 | NSMA2_MOUSE Sphingomyelin phosphodiesterase 3 Smpd3 |
| Novel00376 | 81.16629 | 382.6342 | -2.237 | 2.11E-09 | 6.28E-07 | |
| GS_008611 | 21.08001 | 98.59651 | -2.2257 | 1.12E-09 | 3.49E-07 | ADRB1_XENLA Beta-1 adrenergic receptor adrb1 |
| GS_016109 | 20.27195 | 93.37405 | -2.2035 | 6.09E-07 | 0.00012 | SCG3_MOUSE Secretogranin-3 Scg3 |
| GS_004597 | 33.53328 | 154.3529 | -2.2026 | 2.66E-11 | 1.01E-08 | MREG_DANRE Melanoregulin mreg |
| GS_005905 | 41.65073 | 191.2326 | -2.1989 | 5.80E-06 | 0.00095 | GILT_BOVIN Gamma-interferon-inducible lysosomal thiol reductase IFI30 |
| GS_006243 | 46.06882 | 209.6656 | -2.1862 | 1.52E-05 | 0.00226 | S6A13_MACFA Sodium- and chloride-dependent GABA transporter 2 SLC6A13 |
| GS_021002 | 8.633781 | 39.2703 | -2.1854 | 8.05E-06 | 0.00126 | PNPH_HUMAN Purine nucleoside phosphorylase PNP |
| GS_021141 | 37.53072 | 169.4484 | -2.1747 | 4.76E-12 | 1.98E-09 | CXA5_CHICK Gap junction alpha-5 protein GJA5 |
| GS_001783 | 12.06517 | 54.21062 | -2.1677 | 7.03E-07 | 0.00014 | KNG_ANAMI Kininogen (Fragments) |
| GS_009046 | 17.25429 | 76.74461 | -2.1531 | 1.44E-08 | 3.79E-06 | KIT_TAKRU Mast/stem cell growth factor receptor kit |
| GS_008564 | 36.46248 | 160.9072 | -2.1417 | 5.05E-11 | 1.81E-08 | PRLR_CEREL Prolactin receptor PRLR |
| GS_015155 | 18.90942 | 82.57596 | -2.1266 | 1.60E-08 | 4.16E-06 | PCDH9_HUMAN Protocadherin-9 PCDH9 |
| GS_007401 | 18.48673 | 79.84006 | -2.1106 | 4.29E-08 | 1.06E-05 | SPON1_HUMAN Spondin-1 SPON1 |
| GS_013586 | 34.09485 | 146.0954 | -2.0993 | 0.00043 | 0.04078 | RASEF_HUMAN Ras and EF-hand domain-containing protein RASEF |
| GS_011506 | 30.12589 | 128.0548 | -2.0877 | 3.47E-10 | 1.15E-07 | ARG33_HUMAN Rho guanine nucleotide exchange factor 33 ARHGEF33 |
| GS_006925 | 65.93585 | 275.9091 | -2.0651 | 1.66E-06 | 0.00031 | PGS1_CANFA Biglycan BGN |
| GS_005244 | 7.792067 | 32.53383 | -2.0619 | 5.05E-05 | 0.00641 | |
| GS_010316 | 7.47908 | 31.22339 | -2.0617 | 5.45E-05 | 0.00688 | GPR61_MOUSE Probable G-protein coupled receptor 61 Gpr61 |
| GS_003202 | 9.871579 | 40.69411 | -2.0435 | 3.85E-05 | 0.00503 | CRFR1_CHICK Corticotropin-releasing factor receptor 1 CRHR1 |
| GS_008220 | 20.1216 | 79.23461 | -1.9774 | 1.15E-07 | 2.71E-05 | AHNK_HUMAN Neuroblast differentiation-associated protein AHNAK AHNAK |
| GS_018838 | 30.16915 | 117.719 | -1.9642 | 7.49E-09 | 2.05E-06 | K1C13_ONCMY Keratin, type I cytoskeletal 13 krt13 |
| GS_020582 | 57.56468 | 222.0361 | -1.9475 | 1.08E-10 | 3.80E-08 | TRPM1_RAT Transient receptor potential cation channel subfamily M member 1 Trpm1 |
| Novel00096 | 242.7738 | 935.453 | -1.9461 | 8.12E-05 | 0.00955 | |
| GS_009135 | 26.91729 | 100.2918 | -1.8976 | 3.85E-08 | 9.78E-06 | PDE8B_HUMAN High affinity cAMP-specific and IBMX-insensitive 3',5'-cyclic phosphodiesterase 8B |
| GS_015133 | 20.55664 | 76.22935 | -1.8907 | 3.37E-07 | 7.23E-05 | PRDC1_XENLA Phosphoribosyltransferase domain-containing protein 1 prtfdc1 |
| GS_009601 | 12.49217 | 45.93848 | -1.8787 | 3.30E-05 | 0.00444 | PLD3A_DANRE 1-phosphatidylinositol-4,5-bisphosphate phosphodiesterase delta-3-A plcd3a |
| GS_018722 | 37.75956 | 138.3946 | -1.8739 | 3.27E-07 | 7.09E-05 | |
| GS_014594 | 23.51234 | 85.99608 | -1.8709 | 2.94E-07 | 6.44E-05 | FCAMR_MOUSE High affinity immunoglobulin alpha and immunoglobulin mu Fc receptor Fcamr |
| GS_015735 | 47.70114 | 172.5983 | -1.8553 | 1.71E-09 | 5.15E-07 | LMNA_XENLA Lamin-A lmna |
| GS_016610 | 78.78176 | 282.223 | -1.8409 | 1.78E-10 | 6.09E-08 | PDE10_HUMAN cAMP and cAMP-inhibited cGMP 3',5'-cyclic phosphodiesterase 10A PDE10A |
| GS_008205 | 39.50128 | 140.617 | -1.8318 | 6.48E-06 | 0.00103 | FXI1C_XENLA Forkhead box protein I1c foxi1c |
| GS_021037 | 16.10221 | 57.24474 | -1.8299 | 0.00036 | 0.03427 | I17EL_HUMAN Putative interleukin-17 receptor E-like IL17REL |
| GS_011079 | 9.667309 | 34.01151 | -1.8148 | 0.00054 | 0.0485 | SDK2_MOUSE Protein sidekick-2 Sdk2 |
| GS_005575 | 9.313738 | 32.65683 | -1.81 | 0.00013 | 0.01496 | NCF1_BOVIN Neutrophil cytosol factor 1 NCF1 |

| | | | | | | |
|---|---|---|---|---|---|---|
| GS_005605 | 43.67028 | 151.0734 | -1.7905 | 2.17E-06 | 0.00039 | COEA1_MOUSE Collagen alpha-1(XIV) chain Col14a1 |
| Novel00152 | 26.7023 | 91.01107 | -1.7691 | 5.02E-05 | 0.00641 | |
| GS_017609 | 100.2169 | 332.3031 | -1.7294 | 5.80E-06 | 0.00095 | ENTP5_CHICK Ectonucleoside triphosphate diphosphohydrolase 5 ENTPD5 |
| GS_009156 | 13.22255 | 43.79131 | -1.7276 | 9.35E-05 | 0.01093 | FOXD2_XENLA Forkhead box protein D2 foxd2 |
| GS_012661 | 81.67476 | 270.4512 | -1.7274 | 1.49E-09 | 4.55E-07 | SOX10_CHICK Transcription factor SOX-10 SOX10 |
| GS_021046 | 12.31538 | 40.64987 | -1.7228 | 0.00019 | 0.02056 | WNT7B_CHICK Protein Wnt-7b WNT7B |
| GS_013840 | 22.75552 | 74.61966 | -1.7133 | 3.13E-06 | 0.00054 | FMN2_HUMAN Formin-2 FMN2 |
| GS_003635 | 20.40361 | 66.03142 | -1.6943 | 1.82E-05 | 0.00264 | PDIA1_BOVIN Protein disulfide-isomerase P4HB |
| GS_016650 | 27.70322 | 87.15208 | -1.6535 | 3.17E-06 | 0.00054 | BAI3_MOUSE Brain-specific angiogenesis inhibitor 3 Bai3 |
| GS_013618 | 50.197 | 157.0913 | -1.6459 | 1.39E-07 | 3.21E-05 | PRDM1_HUMAN PR domain zinc finger protein 1 PRDM1 |
| GS_021359 | 95.20214 | 290.75 | -1.6107 | 3.71E-08 | 9.53E-06 | PANX3_MOUSE Pannexin-3 Panx3 |
| GS_018129 | 553.4878 | 1676.511 | -1.5988 | 4.62E-07 | 9.72E-05 | KCRB_HUMAN Creatine kinase B-type CKB |
| GS_015243 | 48.87063 | 145.0143 | -1.5692 | 0.00022 | 0.02347 | VTCN1_RAT V-set domain-containing T-cell activation inhibitor 1 Vtcn1 |
| GS_003795 | 127.131 | 373.7051 | -1.5556 | 3.15E-05 | 0.00426 | PTC1_DANRE Protein patched homolog 1 ptch1 |
| GS_015514 | 496.364 | 1438.544 | -1.5351 | 3.48E-05 | 0.00462 | COBA2_HUMAN Collagen alpha-2(XI) chain COL11A2 |
| Novel00572 | 174.3427 | 503.6639 | -1.5305 | 1.23E-05 | 0.00186 | |
| GS_017985 | 12.69033 | 36.59046 | -1.5277 | 0.00053 | 0.0476 | CCD42_BOVIN Coiled-coil domain-containing protein 42A CCDC42 |
| GS_019376 | 36.75521 | 105.5109 | -1.5214 | 6.17E-06 | 0.00099 | HS3SB_HUMAN Heparan sulfate glucosamine 3-O-sulfotransferase 3B1 HS3ST3B1 |
| GS_006912 | 19.46115 | 55.85013 | -1.521 | 0.00024 | 0.02439 | NTF7_DANRE Neurotrophin-7 ntf7 |
| GS_010330 | 124.9547 | 356.2072 | -1.5113 | 8.12E-09 | 2.17E-06 | CAH6_HUMAN Carbonic anhydrase 6 CA6 |
| GS_014863 | 18.42844 | 52.0318 | -1.4975 | 0.00035 | 0.03402 | NEU1A_HUMAN Neuralized-like protein 1A NEURL |
| GS_016931 | 40.33689 | 112.6337 | -1.4815 | 0.00047 | 0.04324 | TAC2N_HUMAN Tandem C2 domains nuclear protein TC2N |
| GS_016790 | 166.6288 | 462.6071 | -1.4731 | 4.06E-08 | 1.02E-05 | AHI1_HUMAN Jouberin AHI1 |
| GS_014679 | 10648.78 | 29391 | -1.4647 | 0.00017 | 0.01874 | K1C13_ONCMY Keratin, type I cytoskeletal 13 krt13 |
| GS_007694 | 73.15349 | 200.1317 | -1.452 | 2.70E-07 | 5.99E-05 | ALKMO_DANRE Alkylglycerol monooxygenase agmo |
| GS_001171 | 38.69591 | 105.7869 | -1.4509 | 1.53E-05 | 0.00226 | ST17A_RABIT Serine/threonine-protein kinase 17A STK17A |
| GS_004260 | 1226.725 | 3348.968 | -1.4489 | 0.00055 | 0.04891 | FYN_XIPHE Tyrosine-protein kinase Fyn fyn |
| GS_002722 | 57.28891 | 153.5481 | -1.4224 | 2.01E-06 | 0.00036 | LAMB2_MOUSE Laminin subunit beta-2 Lamb2 |
| GS_014502 | 29.04007 | 77.51998 | -1.4165 | 7.01E-05 | 0.00849 | CDK15_DANRE Cyclin-dependent kinase 15 cdk15 |
| GS_010852 | 44.20949 | 116.173 | -1.3938 | 1.78E-05 | 0.00259 | TM119_HUMAN Transmembrane protein 119 TMEM119 |
| GS_018226 | 180.2463 | 456.9367 | -1.342 | 5.43E-07 | 0.00011 | ES1_HUMAN ES1 protein homolog, mitochondrial C21orf33 |
| GS_012647 | 166.5968 | 418.1889 | -1.3278 | 1.12E-06 | 0.00022 | EDNRB_HUMAN Endothelin B receptor EDNRB |
| GS_014776 | 31.102 | 76.87559 | -1.3055 | 0.00015 | 0.01621 | PCD10_HUMAN Protocadherin-10 PCDH10 |
| GS_016685 | 69.94283 | 160.3735 | -1.1972 | 7.12E-05 | 0.00856 | MSMB_PIG Beta-microseminoprotein MSMB |
| GS_008156 | 108.119 | 247.3899 | -1.1942 | 2.50E-05 | 0.00351 | D2_DICDI cAMP-regulated D2 protein D2 |
| GS_013252 | 53.51169 | 122.1862 | -1.1912 | 0.00017 | 0.01841 | SATT_BOVIN Neutral amino acid transporter A SLC1A4 |
| GS_003179 | 538.0699 | 1224.406 | -1.1862 | 1.58E-06 | 0.0003 | |

| | | | | | | |
|---|---|---|---|---|---|---|
| GS_020906 | 76.43969 | 170.2609 | -1.1554 | 9.73E-05 | 0.01131 | BACE2_HUMAN Beta-secretase 2 BACE2 |
| GS_011190 | 138.667 | 308.6202 | -1.1542 | 6.12E-05 | 0.00754 | RPGP1_HUMAN Rap1 GTPase-activating protein 1 RAP1GAP |
| GS_014165 | 72.38483 | 160.5437 | -1.1492 | 0.00013 | 0.01444 | HHIP_HUMAN Hedgehog-interacting protein HHIP |
| GS_010295 | 387.5811 | 858.5141 | -1.1473 | 0.00034 | 0.03285 | MMP9_MOUSE Matrix metalloproteinase-9 Mmp9 |
| GS_015779 | 127.813 | 282.9722 | -1.1466 | 2.07E-05 | 0.00297 | CA216_HUMAN UPF0500 protein C1orf216 C1orf216 |
| GS_003876 | 40.54926 | 88.92702 | -1.1329 | 0.00044 | 0.04117 | PAX7_MOUSE Paired box protein Pax-7 Pax7 |
| GS_015428 | 88.89908 | 190.3594 | -1.0985 | 0.00037 | 0.03517 | CNTFR_CHICK Ciliary neurotrophic factor receptor subunit alpha CNTFR |
| GS_018359 | 263.687 | 561.5324 | -1.0905 | 2.49E-05 | 0.00351 | EDNRB_CANFA Endothelin B receptor EDNRB |
| GS_008287 | 143.483 | 295.7553 | -1.0435 | 0.00026 | 0.02601 | PRS35_HUMAN Inactive serine protease 35 PRSS35 |
| GS_010021 | 178.1393 | 349.724 | -0.97321 | 0.00039 | 0.03748 | PHOP1_DANRE Probable phosphatase phospho1 phospho1 |
| GS_008315 | 291.4098 | 569.3749 | -0.96633 | 0.00025 | 0.02507 | CTGF_PIG Connective tissue growth factor CTGF |
| GS_001946 | 151.6316 | 296.0781 | -0.96541 | 0.00053 | 0.04773 | MYO5A_HUMAN Myosin-Va MYO5A |
| GS_005856 | 519.0089 | 988.4966 | -0.92948 | 0.00022 | 0.02329 | SPON1_CHICK Spondin-1 SPON1 |
| GS_009380 | 2184.317 | 4124.294 | -0.91697 | 0.00052 | 0.04692 | FRPA_NEIMB Iron-regulated protein frpA serogroup B frpA |
| GS_015700 | 347.1862 | 177.6938 | 0.96632 | 0.00048 | 0.04383 | DMBT1_HUMAN Deleted in malignant brain tumors 1 protein DMBT1 |
| GS_012001 | 330.5612 | 169.006 | 0.96784 | 0.00021 | 0.02269 | PLIN2_HUMAN Perilipin-2 PLIN2 |
| GS_010268 | 359.7647 | 181.0233 | 0.99088 | 0.0002 | 0.02125 | VGFAA_DANRE Vascular endothelial growth factor A-A vegfaa |
| GS_000538 | 242.3605 | 119.756 | 1.0171 | 0.00026 | 0.02601 | CP2J2_HUMAN Cytochrome P450 2J2 CYP2J2 |
| GS_020318 | 38280.86 | 18843.51 | 1.0226 | 0.00026 | 0.02631 | DESM_CANFA Desmin DES |
| GS_016447 | 1082.05 | 532.0736 | 1.0241 | 0.00013 | 0.01478 | FA8A1_HUMAN Protein FAM8A1 FAM8A1 |
| GS_002994 | 452.2278 | 218.9195 | 1.0466 | 0.00011 | 0.01236 | SAMP_MESAU Serum amyloid P-component APCS |
| GS_018185 | 224.4158 | 108.3004 | 1.0511 | 0.00033 | 0.0326 | DSCAM_HUMAN Down syndrome cell adhesion molecule DSCAM |
| GS_011788 | 1622.507 | 775.9816 | 1.0641 | 0.00034 | 0.03285 | |
| GS_006756 | 220.2531 | 102.7698 | 1.0997 | 0.00013 | 0.0149 | CF162_HUMAN UPF0708 protein C6orf162 C6orf162 |
| GS_002188 | 193.7795 | 90.27409 | 1.102 | 0.00012 | 0.01401 | AQP7_MOUSE Aquaporin-7 Aqp7 |
| GS_018739 | 878.7827 | 408.9483 | 1.1036 | 0.00038 | 0.03652 | EPD_CYPCA Ependymin epd |
| GS_003073 | 55213.96 | 25616.33 | 1.108 | 0.00053 | 0.0476 | HSP7C_RAT Heat shock cognate 71 kDa protein Hspa8 |
| Novel00681 | 91.3119 | 41.68308 | 1.1313 | 0.0005 | 0.04585 | |
| GS_017957 | 638.6058 | 287.0637 | 1.1536 | 5.85E-06 | 0.00095 | SXT2_TAKPA Saxitoxin and tetrodotoxin-binding protein 2 psbp2 |
| GS_002995 | 286.9177 | 128.6611 | 1.1571 | 5.71E-05 | 0.00712 | CRP_RAT C-reactive protein Crp |
| GS_009938 | 143.7901 | 61.7035 | 1.2205 | 0.0001 | 0.0117 | DHB14_BOVIN 17-beta-hydroxysteroid dehydrogenase 14 HSD17B14 |
| GS_015381 | 720.1119 | 304.6461 | 1.2411 | 2.74E-05 | 0.00383 | MT21C_BOVIN Methyltransferase-like protein 21C METTL21C |
| GS_006488 | 104.8874 | 44.36897 | 1.2412 | 0.00026 | 0.02631 | P2RY8_CHICK P2Y purinoceptor 8 P2RY8 |
| GS_007314 | 1520.171 | 603.0046 | 1.334 | 1.62E-06 | 0.0003 | GRN_HUMAN Granulins GRN |
| GS_011843 | 273.7297 | 105.1519 | 1.3803 | 8.79E-07 | 0.00017 | GNS_MOUSE N-acetylglucosamine-6-sulfatase Gns |
| GS_009020 | 86.26458 | 32.67895 | 1.4004 | 0.0001 | 0.01196 | GROA_BOVIN Growth-regulated protein homolog alpha |
| GS_011555 | 1985.88 | 734.3907 | 1.4352 | 3.19E-06 | 0.00054 | LYSC_PAROL Lysozyme C |

| | | | | | | |
|---|---|---|---|---|---|---|
| GS_010525 | 49.83009 | 18.1153 | 1.4598 | 0.00041 | 0.03902 | FABPI_PIG Fatty acid-binding protein, intestinal FABP2 |
| GS_006200 | 44.03189 | 15.75119 | 1.4831 | 0.00044 | 0.04135 | GRM3_RAT Metabotropic glutamate receptor 3 Grm3 |
| GS_009467 | 129.992 | 45.10918 | 1.5269 | 1.47E-05 | 0.00221 | TNNT2_CHICK Troponin T, cardiac muscle isoforms TNNT2 |
| GS_002131 | 772.4685 | 265.5339 | 1.5406 | 1.10E-09 | 3.45E-07 | MSTN1_XENLA Musculoskeletal embryonic nuclear protein 1 mustn1 |
| GS_007697 | 38.96027 | 12.63197 | 1.6249 | 0.00024 | 0.02443 | SOSD1_MOUSE Sclerostin domain-containing protein 1 Sostdc1 |
| GS_010174 | 5231.999 | 1558.213 | 1.7475 | 6.56E-05 | 0.00803 | LYG_PAROL Lysozyme g |
| GS_006975 | 3661.067 | 865.7575 | 2.0802 | 2.78E-09 | 8.15E-07 | GLRK_RANBE Probable glutamate receptor |
| GS_017925 | 177.8423 | 40.07039 | 2.15 | 0.00015 | 0.01607 | CATL_SARPE Cathepsin L |
| GS_004023 | 24.26654 | 5.355341 | 2.1799 | 0.00022 | 0.02292 | ARL14_HUMAN ADP-ribosylation factor-like protein 14 ARL14 |
| GS_000465 | 58.65324 | 12.18461 | 2.2672 | 1.64E-07 | 3.70E-05 | CO1A2_RABIT Collagen alpha-2(I) chain (Fragment) COL1A2 |
| GS_013015 | 36.71626 | 7.076408 | 2.3753 | 1.44E-06 | 0.00027 | PE2R3_BOVIN Prostaglandin E2 receptor EP3 subtype PTGER3 |
| GS_014186 | 360.8196 | 59.91753 | 2.5902 | 3.53E-06 | 0.00059 | MSLNL_HUMAN Mesothelin-like protein MSLNL |
| GS_008785 | 419.4108 | 59.61546 | 2.8146 | 2.81E-05 | 0.00385 | CAH4_BOVIN Carbonic anhydrase 4 CA4 |
| GS_000336 | 1969.69 | 276.7654 | 2.8312 | 3.55E-26 | 4.35E-23 | VIAAT_XENTR Vesicular inhibitory amino acid transporter slc32a1 |
| GS_007481 | 965.9608 | 88.82826 | 3.4429 | 8.49E-10 | 2.72E-07 | KCC1G_RAT Calcium/calmodulin-dependent protein kinase type 1G Camk1g |
| GS_002121 | 32.00992 | 1.603362 | 4.3193 | 5.66E-07 | 0.00012 | CIDEC_HUMAN Cell death activator CIDE-3 CIDEC |
| GS_019550 | 11.37374 | 0.323018 | 5.1379 | 0.00014 | 0.01515 | CXA3_MOUSE Gap junction alpha-3 protein Gja3 |
| GS_001591 | 17.81749 | 0 | Inf | 3.48E-09 | 1.01E-06 | NKX11_HUMAN NK1 transcription factor-related protein 1 NKX1-1 |
