# Supplementary material for: The integrated analysis of RNA-seq and microRNA-seq depicts miRNA-mRNA networks involved in Japanese flounder (Paralichthys olivaceus) albinism
Source: PLoS One. 2017 Aug 4;12(8):e0181761. doi: 10.1371/journal.pone.0181761 (PMC5544202; doi:10.1371/journal.pone.0181761)
Supplement: S6 Table — (PDF) [file pone.0181761.s010.pdf]

S6 Table. The GO term enrichment for differentially expressed genes in RNA-seq (p≤0.05).

GO_accession	Description	Term_type	P-Value	Corrected P-Value	DEG_item	DEG_list	Bg_item	Bg_list
GO:0005576	extracellular region	cellular_component	9.67E-05	0.39588	30	189	1324	17379
GO:0004888	transmembrane signaling receptor activity	molecular_function	0.000387	0.74866	20	189	789	17379
GO:0007186	G-protein coupled receptor signaling	biological_process	0.000744	0.74866	21	189	894	17379
GO:0004871	signal transducer activity	molecular_function	0.00094	0.74866	25	189	1172	17379
GO:0060089	molecular transducer activity	molecular_function	0.00094	0.74866	25	189	1172	17379
GO:0007166	cell surface receptor signaling pathway	biological_process	0.001098	0.74866	23	189	1052	17379
GO:0004930	G-protein coupled receptor activity	molecular_function	0.001539	0.77915	16	189	633	17379
GO:0004392	heme oxygenase (decyclizing) activity	molecular_function	0.001714	0.77915	2	189	6	17379
GO:0006788	heme oxidation	biological_process	0.001714	0.77915	2	189	6	17379
GO:0004872	receptor activity	molecular_function	0.001924	0.78738	21	189	965	17379
GO:0038023	signaling receptor activity	molecular_function	0.002144	0.79752	20	189	908	17379
GO:0005882	intermediate filament	cellular_component	0.002773	0.86163	6	189	128	17379
GO:0045111	intermediate filament cytoskeleton	cellular_component	0.002773	0.86163	6	189	128	17379
GO:0016491	oxidoreductase activity	molecular_function	0.003148	0.86163	23	189	1142	17379
GO:0048038	quinone binding	molecular_function	0.003159	0.86163	2	189	8	17379
GO:0015291	secondary active transmembrane transporter activity	molecular_function	0.00348	0.88988	6	189	134	17379
GO:0015293	symporter activity	molecular_function	0.005138	1	5	189	102	17379
GO:0015294	solute:cation symporter activity	molecular_function	0.005138	1	5	189	102	17379
GO:0044421	extracellular region part	cellular_component	0.005635	1	13	189	533	17379
GO:0008150	biological_process	biological_process	0.006997	1	151	189	12486	17379
GO:0005578	proteinaceous extracellular matrix	cellular_component	0.007527	1	8	189	259	17379
GO:0007155	cell adhesion	biological_process	0.008886	1	11	189	440	17379
GO:0006787	porphyrin-containing compound catabolic process	biological_process	0.009819	1	2	189	14	17379
GO:0033015	tetrapyrrole catabolic process	biological_process	0.009819	1	2	189	14	17379
GO:0005149	interleukin-1 receptor binding	molecular_function	0.010868	1	1	189	1	17379
GO:0015105	arsenite transmembrane transporter	molecular_function	0.010881	1	1	189	1	17379
GO:0015700	arsenite transport	biological_process	0.010881	1	1	189	1	17379
GO:0016021	integral to membrane	cellular_component	0.010964	1	43	189	2808	17379
GO:0051187	cofactor catabolic process	biological_process	0.011249	1	2	189	15	17379
GO:0043043	peptide biosynthetic process	biological_process	0.011257	1	2	189	15	17379

GO:0015081	sodium ion transmembrane transporter activity	molecular_function	0.011486	1	5	189	124	17379
GO:0022610	biological adhesion	biological_process	0.011925	1	11	189	459	17379
GO:0015370	solute:sodium symporter activity	molecular_function	0.012277	1	4	189	82	17379
GO:0031224	intrinsic to membrane	cellular_component	0.012634	1	43	189	2832	17379
GO:0030203	glycosaminoglycan metabolic process	biological_process	0.012783	1	2	189	16	17379
GO:0031012	extracellular matrix	cellular_component	0.012938	1	9	189	343	17379
GO:0042710	biofilm formation	biological_process	0.016058	1	2	189	18	17379
GO:0005326	neurotransmitter transporter activity	molecular_function	0.016179	1	3	189	49	17379
GO:0005328	neurotransmitter:sodium symporter	molecular_function	0.016179	1	3	189	49	17379
GO:0015629	actin cytoskeleton	cellular_component	0.017013	1	6	189	188	17379
GO:0044430	cytoskeletal part	cellular_component	0.018655	1	9	189	365	17379
GO:0016459	myosin complex	cellular_component	0.020121	1	5	189	143	17379
GO:0005125	cytokine activity	molecular_function	0.02089	1	3	189	54	17379
GO:0046406	magnesium protoporphyrin IX methyltransferase activity	molecular_function	0.021608	1	1	189	2	17379
GO:0003871	5-methyltetrahydropteroyltriglutamate-homocysteine S-methyltransferase activity	molecular_function	0.021628	1	1	189	2	17379
GO:0042085	5-methyltetrahydropteroyltri-L-glutamate-dependent methyltransferase activity	molecular_function	0.021628	1	1	189	2	17379
GO:0006106	fumarate metabolic process	biological_process	0.021628	1	1	189	2	17379
GO:0008940	nitrate reductase activity	molecular_function	0.02163	1	1	189	2	17379
GO:0009325	nitrate reductase complex	cellular_component	0.02163	1	1	189	2	17379
GO:0016661	oxidoreductase activity, acting on other nitrogenous compounds as donors	molecular_function	0.02163	1	1	189	2	17379
GO:0005615	extracellular space	cellular_component	0.023517	1	5	189	149	17379
GO:0006836	neurotransmitter transport	biological_process	0.028758	1	3	189	61	17379
GO:0003774	motor activity	molecular_function	0.031587	1	6	189	217	17379
GO:0043604	amide biosynthetic process	biological_process	0.032234	1	2	189	26	17379
GO:0006545	glycine biosynthetic process	biological_process	0.03226	1	1	189	3	17379
GO:0043163	cell envelope organization	biological_process	0.032283	1	1	189	3	17379
GO:0043165	Gram-negative-bacterium-type cell outer membrane assembly	biological_process	0.032283	1	1	189	3	17379
GO:0044091	membrane biogenesis	biological_process	0.032283	1	1	189	3	17379
GO:0071709	membrane assembly	biological_process	0.032283	1	1	189	3	17379
GO:0008745	N-acetylmuramoyl-L-alanine amidase activity	molecular_function	0.032286	1	1	189	3	17379

GO:0008193	tRNA guanylyltransferase activity	molecular_function	0.03229	1	1	189	3	17379
GO:0008131	primary amine oxidase activity	molecular_function	0.032291	1	1	189	3	17379
GO:0030158	protein xylosyltransferase activity	molecular_function	0.032291	1	1	189	3	17379
GO:0035252	UDP-xylosyltransferase activity	molecular_function	0.032291	1	1	189	3	17379
GO:0042285	xylosyltransferase activity	molecular_function	0.032291	1	1	189	3	17379
GO:0022804	active transmembrane transporter activity	molecular_function	0.036017	1	6	189	224	17379
GO:0008009	chemokine activity	molecular_function	0.041853	1	2	189	30	17379
GO:0042379	chemokine receptor binding	molecular_function	0.041853	1	2	189	30	17379
GO:0005215	transporter activity	molecular_function	0.041977	1	25	189	1602	17379
GO:0001881	receptor recycling	biological_process	0.042774	1	1	189	4	17379
GO:0031047	gene silencing by RNA	biological_process	0.042781	1	1	189	4	17379
GO:0009070	serine family amino acid biosynthetic	biological_process	0.042792	1	1	189	4	17379
GO:0004363	glutathione synthase activity	molecular_function	0.042795	1	1	189	4	17379
GO:0003796	lysozyme activity	molecular_function	0.042797	1	1	189	4	17379
GO:0009086	methionine biosynthetic process	biological_process	0.042806	1	1	189	4	17379
GO:0042393	histone binding	molecular_function	0.042809	1	1	189	4	17379
GO:0006024	glycosaminoglycan biosynthetic process	biological_process	0.042821	1	1	189	4	17379
GO:0006814	sodium ion transport	biological_process	0.043742	1	3	189	72	17379
GO:0005856	cytoskeleton	cellular_component	0.046457	1	9	189	431	17379
GO:0044425	membrane part	cellular_component	0.047625	1	46	189	3342	17379
GO:0009611	response to wounding	biological_process	0.047652	1	4	189	125	17379
