# Supplementary material for: The integrated analysis of RNA-seq and microRNA-seq depicts miRNA-mRNA networks involved in Japanese flounder (Paralichthys olivaceus) albinism
Source: PLoS One. 2017 Aug 4;12(8):e0181761. doi: 10.1371/journal.pone.0181761 (PMC5544202; doi:10.1371/journal.pone.0181761)
Supplement: S7 Table — (PDF) [file pone.0181761.s011.pdf]

S7 Table. The KEGG pathway enrichment for differentially expressed genes in RNA-seq ($p \leqslant 0.05$).

Pathways	ID	Input number	Background number	P-Value	Corrected P-Value
Folate biosynthesis	tru00790	6	19	1.61E-07	9.04E-06
Melanogenesis	tru04916	10	131	1.90E-06	5.31E-05
Neuroactive ligand-receptor interaction	tru04080	11	379	0.002331	0.043509408
Tyrosine metabolism	tru00350	3	35	0.006939	0.078470987
Cytokine-cytokine receptor interaction	tru04060	6	158	0.007006	0.078470987
Butanoate metabolism	tru00650	2	21	0.023155	0.180845259
Hedgehog signaling pathway	tru04340	3	57	0.023907	0.180845259
Purine metabolism	tru00230	6	213	0.025835	0.180845259
Dorso-ventral axis formation	tru04320	2	26	0.033461	0.208202432
