# Supplementary material for: The integrated analysis of RNA-seq and microRNA-seq depicts miRNA-mRNA networks involved in Japanese flounder (Paralichthys olivaceus) albinism
Source: PLoS One. 2017 Aug 4;12(8):e0181761. doi: 10.1371/journal.pone.0181761 (PMC5544202; doi:10.1371/journal.pone.0181761)
Supplement: S8 Table — (PDF) [file pone.0181761.s012.pdf]

S8 Table. Overview of reads from raw data to cleaned sequences in miRNA-seq.

	PO_con1				PO_con2				PO_con3			
lib	Total	% of Total	uniq	% of uniq	Total	% of Total	uniq	% of uniq	Total	% of Total	uniq	% of uniq
Raw reads	9,960,543	100.00	1,340,590	100	10,957,008	100.00	1,714,953	100.00	11,060,368	100.00	1,325,433	100.00
3ADT&length filter	5,115,072	51.35	963,763	71.89	4,879,960	44.54	1,244,396	72.56	3,790,145	34.27	869,460	65.60
Junk reads	27,464	0.28	5,125	0.38	27,423	0.25	5,602	0.33	29,957	0.27	5,891	0.44
Rfam	503,971	5.06	35,578	2.65	797,449	7.28	48,701	2.84	559,333	5.06	40,774	3.08
mRNA	187,144	1.88	97,143	7.25	254,150	2.32	108,869	6.35	217,359	1.97	115,892	8.74
Repeats	304	0.00	83	0.01	456	0.00	106	0.01	533	0.00	122	0.01
valid reads	4,134,252	41.51	239,765	17.89	5,004,942	45.68	308,437	17.99	6,467,034	58.47	294,457	22.22
rRNA	423,701	4.25	23,141	0.23	672,384	6.14	30,968	0.28	469,513	4.25	25,728	0.23
tRNA	41,274	0.41	5,213	0.05	65,413	0.60	8,360	0.08	35,322	0.32	6,340	0.06
snoRNA	4,663	0.05	1,555	0.02	5,636	0.05	1,830	0.02	5,896	0.05	1,844	0.02
snRNA	4,243	0.04	1,586	0.02	6,252	0.06	2,161	0.02	6,704	0.06	2,117	0.02
other Rfam RNA	30,090	0.30	4,083	0.04	47,764	0.44	5,382	0.05	41,898	0.38	4,745	0.04

	PO_alb1				PO_alb2				PO_alb3			
lib	Total	% of Total	uniq	% of uniq	Total	% of Total	uniq	% of uniq	Total	% of Total	uniq	% of uniq
Raw reads	10,559,741	100.00	1,100,934	100.00	9,702,427	100.00	1,397,359	100.00	10,974,117	100.00	1,325,208	100.00
3ADT&length filter	3,600,852	34.10	776,366	70.52	3,587,089	36.97	970,881	69.48	4,102,638	37.38	949,402	71.64
Junk reads	8,889	0.08	3,009	0.27	26,253	0.27	4,819	0.34	26,556	0.24	4,725	0.36
Rfam	498,640	4.72	30,245	2.75	823,819	8.49	39,896	2.86	669,893	6.10	36,012	2.72
mRNA	216,013	2.05	73,351	6.66	278,528	2.87	119,548	8.56	221,772	2.02	90,765	6.85
Repeats	79	0.00	46	0.00	414	0.00	101	0.01	299	0.00	102	0.01
valid reads	6,238,636	59.08	218,445	19.84	4,989,758	51.43	263,097	18.83	5,955,979	54.27	245,000	18.49
rRNA	426,821	4.04	19,195	0.18	723,251	7.45	25,469	0.26	597,114	5.44	23,295	0.21
tRNA	39,404	0.37	5,413	0.05	50,548	0.52	6,616	0.07	36,278	0.33	5,991	0.05
snoRNA	2,332	0.02	1,043	0.01	4,084	0.04	1,455	0.01	3,535	0.03	1,367	0.01
snRNA	2,770	0.03	1,287	0.01	4,146	0.04	1,532	0.02	4,177	0.04	1,406	0.01
other Rfam RNA	27,313	0.26	3,307	0.03	41,790	0.43	4,824	0.05	28,789	0.26	3,953	0.04
