# Supplementary material for: The integrated analysis of RNA-seq and microRNA-seq depicts miRNA-mRNA networks involved in Japanese flounder (Paralichthys olivaceus) albinism
Source: PLoS One. 2017 Aug 4;12(8):e0181761. doi: 10.1371/journal.pone.0181761 (PMC5544202; doi:10.1371/journal.pone.0181761)
Supplement: S9 Table — (PDF) [file pone.0181761.s013.pdf]

S9 Table. 31 unique miRNAs mapping to Japanese flounder miRNA/pre-miRNAs in miRbase and the pre-miRNAs further map to genome & EST.

miRNA_Index	miR_name	miR_seq	genomeID	strand	start	end	group	Expression level
1	pol-miR-1-5p	ACATACTTCTTTATATGCCCATA	scaffold37.1	-	1,918,726	1,918,822	gp1a	middle
2	pol-miR-1-3p_1ss2GT	TTGAATGTAAAGAAGTATGTAT	scaffold37.1	-	1,918,726	1,918,822	gp1a	high
3	pol-let-7a-5p_1ss19GA	TGAGGTAGTAGGTTGTATAGTT	scaffold41.1	-	2,315,191	2,315,286	gp1a	high
4	tni-let-7a-3-p3_1ss22AT	TACAGTCTATTACCTTCCTTGT	scaffold41.1	-	2,315,191	2,315,286	gp1a	middle
5	pol-let-7b-5p	TGAGGTAGTAGGTTGTGTGGTT	scaffold84.1	+	3,267,040	3,267,130	gp1a	high
6	pol-let-7b-3p_R+1	CTATACAACCTACTGCCTTCCC	scaffold84.1	+	3,267,040	3,267,130	gp1a	middle
7	pol-let-7d-5p	TGAGGTAGTTGGTTGTATGGTT	scaffold30.1	-	634,790	634,885	gp1a	high
8	pol-let-7d-3p	CTGTACAACCTTCTAGCTTTCC	scaffold30.1	-	634,790	634,885	gp1a	middle
9	pol-miR-9b-5p_R-2	TCTTTGGTTATCTAGCTGTAT	scaffold32.1	+	637,060	637,150	gp1a	middle
10	pol-miR-9b-3p	TAAAGCTAGAGAACCGAAAGTA	scaffold32.1	+	637,060	637,150	gp1a	middle
11	pol-miR-10b-5p_1ss19GT	TACCCTGTAGAACCGAATTTGT	scaffold50.1	+	1,275,563	1,275,664	gp1a	high
12	pol-miR-10b-3p_L+2R-2	ACAGATTCGATTCTAGGGGAGT	scaffold50.1	+	1,275,563	1,275,664	gp1a	middle
13	pol-miR-21-5p_R+1	TAGCTTATCAGACTGGTGTTGGC	scaffold10.1	+	9,156,699	9,156,779	gp1a	high
14	pol-miR-21-3p_R-1_1ss14A	CGACAACAGTCTGTAGGCTGT	scaffold10.1	+	9,156,699	9,156,779	gp1a	middle
15	pol-miR-22-5p_R-1	AGTTCTTCACTGGCAAGCTTT	scaffold12.1	-	10,545,611	10,545,704	gp1a	middle
16	pol-miR-22-3p	AAGCTGCCAGCTGAAGAACTGT	scaffold12.1	-	10,545,611	10,545,704	gp1a	high
17	pol-miR-122-5p_R+1	TGGAGTGTGACAATGGTGTTTGT	scaffold25.1	-	8,220,260	8,220,366	gp1a	middle
18	pol-miR-133-5p	GCTGGTCAAACGGAACCAAGTC	scaffold14.1	+	5,270,995	5,271,088	gp1a	middle
19	pol-miR-133-3p	TTTGGTCCCCTTCAACCAGCT	scaffold14.1	+	5,270,995	5,271,088	gp1a	high
20	pol-miR-140-5p	CAGTGGTTTTACCCTATGGTAG	scaffold104.1	-	798,530	798,629	gp1a	middle
21	pol-miR-140-3p_R+1	ACCACAGGGTAGAACCACGGACT	scaffold104.1	-	798,530	798,629	gp1a	middle
22	pol-miR-144-5p	GGATATCATCTTATACTGTAAGT	scaffold10.1	-	8,160,012	8,160,085	gp1a	middle
23	pol-miR-144-3p_L+1R-2	CTACAGTATAGATGATGTACT	scaffold10.1	-	8,160,012	8,160,085	gp1a	middle
24	pol-miR-182-5p_R+2	TTTGGCAATGGTAGAACTCACAC	scaffold84.1	+	6,721,855	6,721,950	gp1a	middle
25	pol-miR-199a-5p_R+1	CCCAGTGTTCAGACTACCTGTTC	scaffold25.1	+	10,067,215	10,067,305	gp1a	high
26	pol-miR-199a-3p_R-1	ACAGTAGTCTGCACATTGGTT	scaffold25.1	+	10,067,215	10,067,305	gp1a	high
27	pol-miR-203-5p_R-1	AGTGGTTCTCAACAGTTCAACA	scaffold15.1	-	2,069,503	2,069,574	gp1a	middle
28	pol-miR-203-3p	GTGAAATGTTTAGGACCACTTG	scaffold15.1	-	2,069,503	2,069,574	gp1a	high
29	pol-miR-206-5p	ACATGCTTCCTTATATCCCCAT	scaffold14.1	+	5,270,156	5,270,247	gp1a	middle
30	pol-miR-206-3p_1ss2GT	TTGAATGTAAGGAAGTGTGTGG	scaffold14.1	+	5,270,156	5,270,247	gp1a	high
31	pol-miR-221-3p	AGCTACATTGTCTGCTGGGTTT	scaffold131.1	-	542,505	542,572	gp1a	high
