# Supplementary material for: The integrated analysis of RNA-seq and microRNA-seq depicts miRNA-mRNA networks involved in Japanese flounder (Paralichthys olivaceus) albinism
Source: PLoS One. 2017 Aug 4;12(8):e0181761. doi: 10.1371/journal.pone.0181761 (PMC5544202; doi:10.1371/journal.pone.0181761)
Supplement: S10 Table — (PDF) [file pone.0181761.s014.pdf]

S10 Table. 240 unique miRNAs mapping to selected species (except for Japanese flounder) miRNAs/pre-miRNAs in miRbase and the pre-miRNAs further mapping to the genome & EST.

miRNA_Index	miR_name	miR_seq	genomeID	strand	start	end	group	Expression level
1	mmu-miR-1a-2-5p_R-1	ACATACTTCTTTATGTACCCAT	scaffold44.1	-	1,462,613	1,462,708	gp1b	middle
2	dre-miR-1_R+2_1ss2GT	TTGAATGTAAAGAAGTATGTATTT	scaffold44.1	-	1,462,613	1,462,708	gp1b	middle
3	dre-let-7i	TGAGGTAGTAGTTTGTGCTGTT	scaffold6.1	-	5,626,251	5,626,344	gp1b	high
4	hsa-let-7i-3p	CTGCGCAAGCTACTGCCTTGCT	scaffold6.1	-	5,626,251	5,626,344	gp1b	middle
5	dre-let-7a_R+2	TGAGGTAGTAGGTTGTATAGTTTT	scaffold6.1	+	2,425,339	2,425,444	gp1b	middle
6	ola-let-7a-3p_L-1R+2	TATACAACTTACTGTCTTTCCT	scaffold6.1	+	2,425,339	2,425,444	gp1b	middle
7	dre-let-7c-5p_L+1R-1	TTGAGGTAGTAGGTTGTATGGT	scaffold10.1	+	10,697,574	10,697,669	gp1b	middle
8	dre-let-7c-2-p3	GTTAACTGTACAACCTTCTAGC	scaffold10.1	+	10,697,574	10,697,669	gp1b	middle
9	ipu-let-7a_R+4	TGAGGTAGTAGGTTGTATAGTTTT[illegible]	scaffold63.1	+	1,022,579	1,022,675	gp1b	middle
10	gga-let-7j-3p	CTATACAGTCTATTGCCTTCCT	scaffold63.1	+	1,022,579	1,022,675	gp1b	middle
11	dre-let-7a_L+1	CTGAGGTAGTAGGTTGTATAGTT	scaffold190.1	+	666,164	666,242	gp1b	middle
12	mml-let-7a-2-3p_1ss8GA	CTGTACAACCTCCTAGCTTTCC	scaffold190.1	+	666,164	666,242	gp1b	middle
13	fru-miR-7_R+3	TGGAAGACTAGTGATTTTGTTGTT	scaffold95.1	+	235,572	235,662	gp1b	middle
14	tgu-miR-7-1-3p_R+1_1ss21TA	CAACAAATCACAGTCTGCCAAA	scaffold95.1	+	235,572	235,662	gp1b	middle
15	hsa-let-7b-3p_1ss22CT	CTATACAACCTACTGCCTTCCT	scaffold6.1	+	2,425,979	2,426,072	gp1b	middle
16	ipu-let-7e	TGAGGTAGTAGATTGAATAGTT	scaffold41.1	-	2,315,483	2,315,579	gp1b	high
17	ipu-let-7e-2-p3	CTATACAATCTACTGTCTTTCT	scaffold63.1	+	1,022,293	1,022,392	gp1b	middle
18	dre-let-7g	TGAGGTAGTAGTTTGTATAGTT	scaffold179.1	+	78,126	78,211	gp1b	high
19	tni-let-7j_1ss11TG	TGAGGTAGTTGTTTGTACAGTT	scaffold60.1	-	2,345,744	2,345,826	gp1b	high
20	tni-let-7j-p3_1ss22CT	CTGTACAAGTGACTGCCTTGCT	scaffold60.1	-	2,345,744	2,345,826	gp1b	middle
21	ipu-let-7f	TGAGGTAGTAGATTGTATAGTT	scaffold61.1	-	1,455,204	1,455,289	gp1b	high
22	hsa-let-7f-1-3p_1ss22CT	CTATACAATCTATTGCCTTCCT	scaffold61.1	-	1,455,204	1,455,289	gp1b	middle
23	ipu-let-7a_R+1	TGAGGTAGTAGGTTGTATAGTTG	scaffold10.1	-	4,237,574	4,237,669	gp1b	middle
24	dre-let-7d-5p_R+2	TGAGGTAGTTGGTTGTATGGTTTT	scaffold69.1	+	273,081	273,156	gp1b	middle
25	hsa-let-7f-2-3p_1ss22CT	CTATACAGTCTACTGTCTTTCT	scaffold100.1	-	494,766	494,855	gp1b	middle
26	ipu-let-7h	TGAGGTAGTAAGTTGTGTTGTT	scaffold100.1	-	494,487	494,582	gp1b	high

27	tni-let-7h-p3	CTATACAACTTACTGCCTTCCT	scaffold100.1	-	494,487	494,582	gp1b	middle
28	dre-let-7a-1-p3_1ss5AT	CTATTCAACCTACTGTCTTTCT	scaffold61.1	-	1,455,400	1,455,489	gp1b	middle
29	dre-miR-9-3p_R+1	TAAAGCTAGATAACCGAAAGTA	scaffold170.1	+	603,112	603,215	gp1b	middle
30	dre-miR-9-4-3p	TAAAGCTAGAGAACCGAATGTA	scaffold3.1	+	7,442,086	7,442,181	gp1b	middle
31	tni-miR-10c	TACCCTGTAGATCCGGATTTGT	scaffold27.1	-	608,983	609,084	gp1b	middle
32	tni-mir-10c-p3	CAAATTCGCTTCTAGGGGAGT	scaffold27.1	-	608,983	609,084	gp1b	middle
33	dre-miR-10b-5p_L+1R-1	GTACCCTGTAGAACCGAATTTGT	scaffold63.1	+	393,026	393,111	gp1b	middle
34	dre-miR-10b-2-3p	CAAATACGTCTCTACAGGAAT	scaffold63.1	+	393,026	393,111	gp1b	middle
35	ola-mir-10d-p3	CAGATTGGGTTCTAGGGGAGTC	scaffold91.1	+	738,762	738,867	gp1b	middle
36	tni-miR-15a_R-1	TAGCAGCACGGAATGGTTTGT	scaffold24.1	+	3,010,360	3,010,426	gp1b	high
37	tni-miR-15b_R-1_1ss21TA	TAGCAGCGCATCATGGTTTGA	scaffold12.1	+	7,352,918	7,353,007	gp1b	high
38	tni-mir-15b-p3_1ss4AG	TGCGAACCATAATTTGCTGCTT	scaffold12.1	+	7,352,918	7,353,007	gp1b	middle
39	ola-miR-16	TAGCAGCACGTAAATATTGGC	scaffold12.1	+	7,353,138	7,353,232	gp1b	high
40	ola-mir-16-p3_1ss10AG	CCCAATATTGGCAGTGCTGCTT	scaffold12.1	+	7,353,138	7,353,232	gp1b	middle
41	tni-miR-16_R-1	TAGCAGCACGTAAATATTGGA	scaffold24.1	+	3,010,583	3,010,654	gp1b	high
42	tni-mir-16-p3_1ss5GA	TCCAATATTGATCGTGCTGCTG	scaffold24.1	+	3,010,583	3,010,654	gp1b	middle
43	ola-miR-17_R+4	CAAAGTGCTTACAGTGCAGGTA	scaffold50.1	-	2,083,359	2,083,449	gp1b	middle
44	ola-mir-17-p3	CTGCAGTGAAGGCACTTTCAGC	scaffold50.1	-	2,083,359	2,083,449	gp1b	middle
45	ipu-miR-17b_L-1_2ss19CA20AC	CTGCAGTGGAGGCACTTACAGC	scaffold145.1	+	643,746	643,823	gp1b	middle
46	dre-miR-18b-5p_1ss11TC	TAAGGTGCATCTAGTGCAGATA	scaffold145.1	+	643,869	643,960	gp1b	middle
47	dre-miR-18b-3p_L+1R-2_1ss15C[illegible]	ACTGCCCTAAGTGCTCCTTCT	scaffold145.1	+	643,869	643,960	gp1b	middle
48	ipu-miR-19b	AGTTTTGCTGGTTTGCATTCAGC	scaffold145.1	+	644,377	644,438	gp1b	middle
49	ipu-mir-19b-p3	TGTGCAAATCCATGCAAAACTG	scaffold145.1	+	644,377	644,438	gp1b	middle
50	rno-miR-19a-3p	TGTGCAAATCTATGCAAAACTGA	scaffold50.1	-	2,082,732	2,082,805	gp1b	middle
51	dre-miR-19a-5p_L+1R-1	GCTAGTTTTGCATAGTTGCACT	scaffold145.1	+	644,014	644,096	gp1b	middle
52	ola-mir-19d-p5	AGCTTTGCAGGGTGGGCAGTCAGC	scaffold46.1	+	1,401,209	1,401,298	gp1b	middle
53	ola-miR-19d	TGTGCAAACCCATGCAAAACTG	scaffold46.1	+	1,401,209	1,401,298	gp1b	middle
54	ola-mir-19b-1-p5	AGTTTTGCTGGTTTGCTTTCAGC	scaffold50.1	-	2,082,427	2,082,515	gp1b	middle
55	tni-miR-20	TAAAGTGCTTATAGTGCAGGTAG	scaffold50.1	-	2,082,558	2,082,634	gp1b	high
56	hhi-miR-21_L+3	ATCTAGCTTATCAGACTGGTGTTG[illegible]	scaffold18.1	+	3,108,771	3,108,855	gp1b	middle
57	hhi-mir-21-p3_1ss8AG	CAACACTGGTTTGTAAGCTGGC	scaffold18.1	+	3,108,771	3,108,855	gp1b	middle
58	ola-miR-22_R+1	AAGCTGCCAGCTGAAGAACTGC	scaffold74.1	-	153,368	153,457	gp1b	middle

59	tni-mir-22b-p5	CGTTCTTCACTGGCTAGCTTTA	scaffold94.1	+	1,257,066	1,257,151	gp1b	middle
60	tni-miR-22b	AAGCTGCCAGTTGAAGAGCTGT	scaffold94.1	+	1,257,066	1,257,151	gp1b	middle
61	dre-miR-23a-3-5p	GGATTCCTGGCAGAGTGATTT	scaffold44.1	-	1,750,784	1,750,859	gp1b	middle
62	dre-miR-23a-3p_R-1	ATCACATTGCCAGGGATTTCC	scaffold44.1	-	1,750,784	1,750,859	gp1b	high
63	dre-mir-23a-1-p5_1ss15CG	AGGGTTCCTGGCACGGTGATTT	scaffold22.1	-	245,675	245,762	gp1b	middle
64	hhi-mir-23a-p5	GGAATTCCTGGCAGGGTGATTT	328,521,904	+	307	401	gp1b	middle
65	bta-miR-23b-5p_1ss12AG	GGGTTCCTGGCGTGCTGATTT	scaffold95.1	+	1,759,104	1,759,209	gp1b	middle
66	ipu-miR-23b	ATCACATTGCCAGGGATTACC	scaffold95.1	+	1,759,104	1,759,209	gp1b	middle
67	dre-mir-24-5-p5	TGCCTGCTGTGCTGATAATCAGT	scaffold90.1	+	688,364	688,447	gp1b	middle
68	dre-miR-24_R+1	TGGCTCAGTTCAGCAGGAACAGT	scaffold90.1	+	688,364	688,447	gp1b	high
69	dre-miR-25-5p_2ss18GA19CT	AGGCGGAGACTTGGGCAATTGCC	scaffold46.1	+	1,401,404	1,401,490	gp1b	middle
70	dre-miR-25-3p	CATTGCACTTGTCTCGGTCTGA	scaffold46.1	+	1,401,404	1,401,490	gp1b	high
71	hhi-miR-26_R+1	TTCAAGTAATCCAGGATAGGCT	scaffold23.1	+	1,249,072	1,249,160	gp1b	high
72	hhi-mir-26-2-p3	CCTATTCAGGACGACTTGGTTC	scaffold23.1	+	1,249,072	1,249,160	gp1b	middle
73	ola-mir-26-2-p3	CCTATTCATGATTACTTGCACT	scaffold32.1	-	5,486,548	5,486,633	gp1b	middle
74	ola-mir-26-1-p3	CCTATTCTTGATTACTTGTTTC	scaffold62.1	-	2,247,962	2,248,043	gp1b	middle
75	hhi-mir-26-1-p3	CCTATGCTTGATTACTTGCACT	scaffold35.1	+	6,605,496	6,605,585	gp1b	middle
76	mmu-miR-27b-5p	AGAGCTTAGCTGATTGGTGAAC	scaffold95.1	+	1,759,465	1,759,539	gp1b	middle
77	ipu-miR-27b	TTCACAGTGGCTAAGTTCTGC	scaffold95.1	+	1,759,465	1,759,539	gp1b	high
78	ola-miR-27b-5p	AGAGCTTAGCTAATTGGTGAGC	scaffold22.1	-	245,038	245,137	gp1b	middle
79	ola-miR-27d-3p_R+3	TTCACAGTGGCTAAGTTCAGT	scaffold22.1	-	245,038	245,137	gp1b	high
80	tni-mir-27c-p5_1ss9AG	CAGGACTTGACCCACATGTGAACA	328,521,904	+	529	602	gp1b	middle
81	tni-miR-27c	TTCACAGTGGTTAAGTTCTGC	328,521,904	+	529	602	gp1b	high
82	ola-miR-27a_R+1	TTCACAGTGGCTAAGTTCCGC	scaffold44.1	-	1,749,981	1,750,063	gp1b	high
83	hsa-miR-29b-1-5p_2ss11TG19TC	GCTGGTTTCAGATGGTGGCTTAGA	scaffold62.1	+	883,204	883,297	gp1b	middle
84	ola-miR-29b	TAGCACCATTTGAAATCAGTGT	scaffold62.1	+	883,204	883,297	gp1b	middle
85	mdo-miR-29a-1-5p_L+1_1ss3TC	ACCGATTTCTTTTGGTGTTCAGA	scaffold62.1	+	883,445	883,529	gp1b	middle
86	ola-miR-29c_R+4	TAGCACCATTTGAAATCGGTTA	scaffold62.1	+	883,445	883,529	gp1b	middle
87	cfa-miR-30d_R-1	TGTAAACATCCCCGACTGGAAGC	scaffold9.1	+	3,842,751	3,842,812	gp1b	high
88	dre-miR-30e-5p_R+2	TGTAAACATCCTTGACTGGAAGCT	scaffold7.1	-	4,318,671	4,318,765	gp1b	high
89	dre-miR-30e-3p	CTTTCAGTCGGATGTTTGCAGC	scaffold7.1	-	4,318,671	4,318,765	gp1b	middle
90	tni-miR-30b	TGTAAACATCCTACACTCAGCT	scaffold9.1	+	3,842,972	3,843,046	gp1b	high

91	tni-mir-30b-p3_1ss22AT	TGAGAGAAGGTTGTTTACTTGT	scaffold9.1	+	3,842,972	3,843,046	gp1b	middle
92	mdo-miR-30c-5p_R+1	TGTAAACATCCTACACTCTCAGCT	scaffold9.1	+	10,248,987	10,249,043	gp1b	high
93	xtr-miR-33a_R+1	GTGCATTGTAGTTGCATTGC	scaffold92.1	+	1,994,279	1,994,367	gp1b	middle
94	dre-miR-34a	TGGCAGTGTCTTAGCTGGTTGT	scaffold19.1	-	7,523,852	7,523,937	gp1b	middle
95	dre-mir-34a-p3_1ss5TA	TGTGAGGAGTGAGAACGAAGC	scaffold19.1	-	7,523,852	7,523,937	gp1b	middle
96	tni-miR-92	TATTGCACTTGTCCCGGCCTGT	scaffold145.1	+	644,564	644,626	gp1b	high
97	dre-miR-92a-5p_2ss10TG11TA	AGGTTGGGAGAGGTAGCAATGCT	scaffold50.1	-	2,082,332	2,082,409	gp1b	middle
98	ipu-miR-96	TTTGGCACTAGCACATTTTTGCT	scaffold84.1	+	6,721,303	6,721,397	gp1b	middle
99	ola-mir-99-p5	AACCCGTAGATCCGATCTTGTG	scaffold10.1	+	10,697,202	10,697,290	gp1b	high
100	ola-miR-99_R-1	CAAGCTCGCCTCTGTGGGTCT	scaffold10.1	+	10,697,202	10,697,290	gp1b	middle
101	dre-miR-100-5p	AACCCGTAGATCCGAACTTGTG	scaffold10.1	-	4,238,207	4,238,304	gp1b	high
102	dre-miR-100-2-3p_1ss10GA	CAAGCTCGTATCTATAGGTATG	scaffold10.1	-	4,238,207	4,238,304	gp1b	middle
103	ola-miR-101b-5p	TCAGTTATCATGGTACCGGTGCT	scaffold45.1	+	2,136,392	2,136,483	gp1b	middle
104	ipu-miR-101b_R+1_1ss10AG	TACAGTACTGTGATAACTGAAG	scaffold45.1	+	2,136,392	2,136,483	gp1b	middle
105	ola-miR-101a-5p	TCAGTTATCACAGTGCTGATGC	scaffold29.1	+	2,136,133	2,136,237	gp1b	middle
106	ola-miR-103_R+2	AGCAGCATTGTACAGGGCTATGA	scaffold46.1	+	1,686,947	1,687,036	gp1b	middle
107	ipu-mir-103-1-p5	AGCCTCTTTACGGTGCTGCCTTG	scaffold178.1	-	455,699	455,764	gp1b	middle
108	dre-miR-107a-3p_R-1	AGCAGCATTGTACAGGGCTATC	scaffold91.1	+	2,266,392	2,266,476	gp1b	middle
109	ccr-miR-124a_R+2	TCAAGGTCCGCTGTGAACACGA	scaffold77.1	-	3,053,679	3,053,744	gp1b	middle
110	dre-miR-125b-5p	TCCCTGAGACCCTAACTTGTGA	scaffold10.1	-	4,232,832	4,232,929	gp1b	high
111	dre-miR-125b-2-3p_L+1_1ss15C	ACGGGTTGGGTTCTTGGGAGCT	scaffold10.1	-	4,232,832	4,232,929	gp1b	middle
112	ola-mir-125b-2-p3	ACGGGTTAGGCTCTTGGGACGC	scaffold10.1	+	10,705,640	10,705,747	gp1b	middle
113	dre-miR-125a	TCCCTGAGACCCTTAACCTGTG	scaffold30.1	-	628,609	628,693	gp1b	high
114	ola-miR-125a-3p_R-1	ACAGGTGAGGTCCTCGGGAAC	scaffold30.1	-	628,609	628,693	gp1b	middle
115	ipu-mir-125a-2-p3	CAGGTGAGGTCCTTGGGAAC	scaffold69.1	+	277,246	277,306	gp1b	middle
116	ola-mir-125c-p3_1ss8TA	ACGGGTTAGGCTCTCGGGAGCT	scaffold190.1	+	671,578	671,671	gp1b	middle
117	ipu-miR-126b	CATTATTACTTTTGGTACGCG	scaffold25.1	+	5,187,933	5,188,032	gp1b	middle
118	ipu-miR-126a	TCGTACCGTGAGTAATAATGCA	scaffold25.1	+	5,187,933	5,188,032	gp1b	high
119	tni-mir-128-1-p5_1ss18TC	CGGGGCCGGGACGCTGTCTGAGA	scaffold203.1	-	226,615	226,686	gp1b	middle
120	tni-miR-128_R-1	TCACAGTGAACCGGTCTCTTT	scaffold203.1	-	226,615	226,686	gp1b	middle
121	ola-mir-128-p5	GGGGGCCGTTACACTGTCAGAGA	scaffold9.1	-	10,037,570	10,037,657	gp1b	middle
122	dre-miR-129-5p_R-1	CTTTTTGCGGTCTGGGCTTGC	scaffold48.1	+	2,599,195	2,599,271	gp1b	middle

123	dre-miR-129-1-3p_L-1R+2	AAGCCCTTACCCCAAAAAGTAT	scaffold48.1	+	2,599,195	2,599,271	gp1b	middle
124	dre-miR-129-3p	AAGCCCTTACCCCAAAAAGCAT	scaffold6.1	+	3,602,564	3,602,655	gp1b	middle
125	gga-miR-130a-5p_1ss16TC	GCCCTTTTTCTGTTGCACTACT	scaffold45.1	-	1,205,380	1,205,483	gp1b	middle
126	ola-miR-130c_R+3	CAGTGCAATATTAAAAGGGCAT	scaffold45.1	-	1,205,380	1,205,483	gp1b	middle
127	dre-miR-130c-5p	GCCCTTTTTCTGTTGTACTACT	scaffold2.1	-	9,898,806	9,898,887	gp1b	middle
128	ccr-miR-132b	ACCATGGCTGTAGACTGTTACC	scaffold74.1	-	582,727	582,814	gp1b	middle
129	dre-miR-132-3p	TAACAGTCTACAGCCATGGTCG	scaffold12.1	-	5,452,002	5,452,091	gp1b	middle
130	dre-miR-133a-5p	AGCTGGTAAAATGGAACCAAAT	scaffold37.1	-	1,904,021	1,904,118	gp1b	middle
131	dre-miR-133a-3p_L-1R+1	TTGGTCCCCTTCAACCAGCTGT	scaffold37.1	-	1,904,021	1,904,118	gp1b	high
132	tni-miR-135b_R+1	TATGGCTTTTTATTCCTATCTGA	scaffold19.1	+	3,902,521	3,902,605	gp1b	middle
133	ola-miR-135b_R+3	TATGGCTTTTTATTCCTACGTG	scaffold84.1	+	5,812,433	5,812,522	gp1b	middle
134	dre-miR-135c	TATGGCTTTCTATTCCTATGTG	scaffold61.1	-	427,876	427,966	gp1b	middle
135	dre-miR-137-3p_L-1R+1	TATTGCTTAAGAATACGCGTAG	scaffold117.1	+	580,472	580,559	gp1b	middle
136	tni-miR-137_R-1	TTATTGCTTGAGAATACGCGT	scaffold1.1	-	101,140	101,209	gp1b	middle
137	dre-miR-138-5p_R+1	AGCTGGTGTTGTGAATCAGGCCG	scaffold93.1	-	681,018	681,110	gp1b	middle
138	ola-miR-139_R+2	TCTACAGTGCATGTGTCTCCAGT	scaffold18.1	-	5,201,436	5,201,528	gp1b	middle
139	ipu-miR-141_R+1	TAACACTGTCTGGTAACGATGT	scaffold19.1	+	2,364,687	2,364,778	gp1b	high
140	tni-mir-200a-p5_1ss11GT	CATCTTACCTTACAGTGCTGGA	scaffold19.1	+	2,364,687	2,364,778	gp1b	middle
141	dre-miR-142a-5p	CATAAAGTAGAAAGCACTACT	scaffold12.1	-	4,710,550	4,710,645	gp1b	middle
142	dre-miR-142a-3p_L-1	GTAGTGTTTCCTACTTTATGGA	scaffold12.1	-	4,710,550	4,710,645	gp1b	middle
143	mmu-miR-143-5p_R+2	GGTGCAGTGCTGCATCTCTGGTC	scaffold53.12	-	599,891	599,948	gp1b	middle
144	ipu-miR-143	TGAGATGAAGCACTGTAGCTC	scaffold53.12	-	599,891	599,948	gp1b	middle
145	ipu-miR-145	GTCCAGTTTTCCCAGGAATCCCT	scaffold53.12	-	598,659	598,721	gp1b	high
146	ipu-mir-145-p3	GGATTCCTGGAAATACTGTTCT	scaffold53.12	-	598,659	598,721	gp1b	middle
147	hhi-miR-147b_L-3R+1_1ss12CA	TGCGGAAAAGCTTCTGCTCCA	scaffold80.1	+	459,129	459,211	gp1b	middle
148	ola-mir-148-p5	AAGTTCTGTGATACACTCAGACT	scaffold50.1	+	1,403,375	1,403,468	gp1b	middle
149	dre-miR-148_R-1	TCAGTGCATTACAGAACTTTG	scaffold50.1	+	1,403,375	1,403,468	gp1b	middle
150	tni-mir-152-p5	AAGTTCTGTGATACACTCTGACT	scaffold27.1	-	366,161	366,232	gp1b	middle
151	tni-miR-152	TCAGTGCATAACAGAACTTTGT	scaffold27.1	-	366,161	366,232	gp1b	middle
152	gga-miR-155_R+1	TTAATGCTAATCGTGATAGGGGT	scaffold145.1	+	624,805	624,862	gp1b	middle
153	mmu-miR-181b-5p	AACATTCATTGCTGTCGGTGGGT	scaffold32.1	-	353,174	353,256	gp1b	middle
154	oan-miR-181a-5p	AACATTCAACGCTGTCGGTGAGT	scaffold53.11	+	1,278,407	1,278,467	gp1b	high

155	ipu-mir-181a-3-p3	ACCATCGACCGTTGACTGTGCC	scaffold32.1	-	353,392	353,456	gp1b	middle
156	mdo-miR-181b-2-3p_L+1_1ss21A	CTCACTGATCAATGAATGCAGA	scaffold53.11	+	1,278,627	1,278,733	gp1b	middle
157	tni-miR-181a-3p_1ss20AT	ACCATCGACCGTTGATTGTTCC	scaffold55.1	+	1,154,664	1,154,736	gp1b	middle
158	ola-miR-183-5p_L+1R+3	TATGGCACTGGTAGAATTCACTG	scaffold48.1	-	1,418,024	1,418,127	gp1b	middle
159	ola-miR-184-5p_L-1R+2	CCTTATCACTTTTCCAGCCCAGC	scaffold52.1	+	3,182,499	3,182,596	gp1b	middle
160	dre-miR-184	TGGACGGAGAACTGATAAGGGC	scaffold52.1	+	3,182,499	3,182,596	gp1b	middle
161	ola-miR-187_R+4	GGCTGCAACACAGGACATGGGT	scaffold9.1	-	9,650,883	9,650,979	gp1b	middle
162	dre-miR-187_R+3	TCGTGTCTTGTGTTGCAGCCAGT	scaffold9.1	-	9,650,883	9,650,979	gp1b	middle
163	dre-miR-190a_R+1	TGATATGTTTGATATATTAGGTT	scaffold52.1	+	2,856,315	2,856,429	gp1b	middle
164	hsa-miR-190a-3p_L+1	ACTATATATCAAACATATTCCT	scaffold52.1	+	2,856,315	2,856,429	gp1b	middle
165	ipu-miR-190b	TGATATGTTTGATATTCGGTTG	scaffold31.1	+	4,133,970	4,134,034	gp1b	middle
166	ipu-miR-192	ATGACCTATGAATTGACAGCC	scaffold10.1	+	7,345,600	7,345,672	gp1b	middle
167	ipu-mir-192-p3	CCTGTCAGTTCTGTAGGCCACT	scaffold10.1	+	7,345,600	7,345,672	gp1b	middle
168	tni-mir-193-p5	TGGGTCTTTGCGGGCAAGGTGA	scaffold1.1	-	333,232	333,317	gp1b	middle
169	tni-miR-193_R-2	AACTGGCCTACAAAGTCCCA	scaffold1.1	-	333,232	333,317	gp1b	middle
170	hhi-miR-196_R+1	TAGGTAGTCTCATGTTGTTGGGT	scaffold30.1	-	1,431,710	1,431,796	gp1b	middle
171	hhi-mir-196-p3	CCACAACACGAAACTGCCTTGA	scaffold30.1	-	1,431,710	1,431,796	gp1b	middle
172	dre-miR-196a-5p	TAGGTAGTTTCATGTTGTTGGG	scaffold9.1	+	7,809,835	7,809,910	gp1b	middle
173	dre-miR-196a-3p_L+1R-1_1ss9G	TCTGCAACATGAAACTGTCTTA	scaffold9.1	+	7,809,835	7,809,910	gp1b	middle
174	dre-miR-196b	TAGGTAGTTTCAAGTTGTTGGG	scaffold27.1	-	711,920	711,988	gp1b	middle
175	dre-mir-196b-p3	ACAGGAACCTGAAACTGCCTGA	scaffold27.1	-	711,920	711,988	gp1b	middle
176	dre-miR-199-5p_R-1_1ss17CG	CCCAGTGTTCAGACTAGCTGTT	scaffold82.1	+	752,238	752,332	gp1b	middle
177	dre-miR-199-3-3p_L+1_1ss11CT	AACAGTAGTCTGCACATTGGTT	scaffold82.1	+	752,238	752,332	gp1b	middle
178	dre-miR-199-5p_L+2R-2	ATCCCAGTGTTCAGACTACCTGT	scaffold3.1	+	6,341,316	6,341,405	gp1b	middle
179	tni-mir-200b-p5	CATCTTACGAGGCAGCATTGGA	scaffold19.1	+	2,364,523	2,364,597	gp1b	middle
180	tni-miR-200b	TAATACTGCCTGGTAATGATGA	scaffold19.1	+	2,364,523	2,364,597	gp1b	high
181	dre-miR-202-5p_R-1	TTCCTATGCATATACCTCTTT	scaffold7.1	+	2,489,674	2,489,747	gp1b	middle
182	dre-miR-204-5p	TTCCCTTTGTCATCCTATGCCT	scaffold2.1	-	1,829,752	1,829,838	gp1b	high
183	tni-mir-204a-p3	GCAGGGACAGCAAAGGGATGCT	scaffold52.1	-	2,330,441	2,330,533	gp1b	middle
184	tni-miR-205	TCCTTCATTCCACCGGAGTCTG	scaffold62.1	-	578,650	578,729	gp1b	high
185	ola-miR-205_L-1R+1_1ss20TA	GATTTCAGTGGTGTGAAGAGTA	scaffold62.1	-	578,650	578,729	gp1b	middle
186	dre-miR-210-5p	AGCCACTGACTAACGCACATTG	scaffold57.1	-	2,639,115	2,639,211	gp1b	middle

187	dre-miR-210-3p_R+1	CTGTGCGTGTGACAGCGGCTAAT	scaffold57.1	-	2,639,115	2,639,211	gp1b	middle
188	ipu-miR-212	ACCTTGGCTCTAGACTGCTTACT	scaffold74.1	+	582,372	582,438	gp1b	middle
189	cgr-miR-214-5p	TGCCTGTCTACACTTGCTGTGC	scaffold3.1	+	6,343,701	6,343,797	gp1b	middle
190	ipu-miR-214_1ss22AT	ACAGCAGGCACAGACAGGCAGT	scaffold3.1	+	6,343,701	6,343,797	gp1b	high
191	tni-miR-216b	TAATCTCTGCAGGCAACTGTGA	scaffold16.1	-	2,445,520	2,445,586	gp1b	middle
192	tni-miR-216a	AAATCTCAGCTGGCAACTGTGA	scaffold16.1	-	2,444,783	2,444,855	gp1b	middle
193	tni-miR-217_R+1	TACTGCATCAGGAACTGATTGGC	scaffold16.1	-	2,444,199	2,444,293	gp1b	middle
194	ola-miR-218b_R+2	TTGTGCTTGATCTAACCATGTG	scaffold185.1	-	18,356	18,452	gp1b	middle
195	ipu-miR-218a	TTGTGCTTGATCTAACCATG	scaffold45.1	-	1,139,168	1,139,247	gp1b	middle
196	ipu-mir-221-1-p5	ACCTGGCATACAATGTAGATTT	scaffold129.1	+	462,014	462,082	gp1b	middle
197	xtr-miR-222_R-1	AGCTACATCTGGCTACTGGGTCT	scaffold131.1	-	542,752	542,813	gp1b	high
198	ipu-miR-222a_1ss11TG	TGCTCAGTAGGCAGTGTAGATC	scaffold129.1	+	461,567	461,630	gp1b	middle
199	ola-mir-223-p5_1ss18GA	TGTATTTGACAAGCTGAATTGG	scaffold10.1	-	5,466,813	5,466,909	gp1b	middle
200	ola-miR-223_R+2	TGTCAGTTTGTCAAATACCCCA	scaffold10.1	-	5,466,813	5,466,909	gp1b	middle
201	dre-miR-301c-5p_R+1_2ss4CT9C	GCTTTGACAATGTTGCACTACT	scaffold45.1	-	1,204,926	1,205,020	gp1b	middle
202	dre-miR-301c-3p_R+1	CAGTGCAATAGTATTGTCATAGC	scaffold45.1	-	1,204,926	1,205,020	gp1b	middle
203	ipu-miR-301a	CAGTGCAATAGTATTGTCAAAGC	scaffold210.1	-	152,441	152,539	gp1b	middle
204	ipu-mir-338-3-p5	AACAATATCCTGGTGCTGCCTGAGT	scaffold123.1	+	2,279,197	2,279,255	gp1b	middle
205	ipu-miR-338_R-1	TCCAGCATCAGTGATTTTGTT	scaffold123.1	+	2,279,197	2,279,255	gp1b	middle
206	tni-mir-338-p5	AACAACATCCTGGTGCTGCCTGAGT	scaffold82.1	-	1,440,088	1,440,163	gp1b	middle
207	ipu-miR-365_R+3	AGGGACTTTTAGGGGCAGCTGTG	scaffold1.1	-	330,505	330,602	gp1b	middle
208	dre-miR-365	TAATGCCCCTAAAAATCCTTAT	scaffold1.1	-	330,505	330,602	gp1b	high
209	tni-miR-375_R-1	TTTGTTCGTTCGGCTCGCGTT	scaffold8.1	-	5,438,710	5,438,781	gp1b	high
210	dre-miR-429a	TAATACTGTCTGGTAATGCCGT	scaffold19.1	+	2,366,230	2,366,316	gp1b	middle
211	ccr-mir-454b-p5	ACCCTATCAATATTGCCTCTGCT	scaffold2.1	-	9,892,572	9,892,642	gp1b	middle
212	ccr-miR-454b_R+2	TAGTGCAATATTGCTTATAGGGT	scaffold2.1	-	9,892,572	9,892,642	gp1b	middle
213	dre-miR-455-5p	TATGTGCCCTTGGACTACATCG	scaffold147.1	+	105,384	105,471	gp1b	middle
214	dre-miR-455-3p_L-1	TGCAGTCCATGGGCATATACAC	scaffold147.1	+	105,384	105,471	gp1b	high
215	ola-miR-460-5p_R+1	CCTGCATTGTACACACTGTGC	scaffold44.1	-	1,182,061	1,182,145	gp1b	middle
216	ola-miR-460-3p_L+1	CACAGCGCATACAATGTGGATG	scaffold44.1	-	1,182,061	1,182,145	gp1b	middle
217	ola-miR-462_L-1R+5	TAACGGAACCCATAATGCAGCTG	scaffold19.1	+	3,953,874	3,953,941	gp1b	middle
218	ola-mir-462-p3_1ss12GC	GCTGGTTATGGCGTCCGTTTCC	scaffold19.1	+	3,953,874	3,953,941	gp1b	middle

219	gga-miR-489-5p	TGGTCGTATGTATGACGTCATT	scaffold9.1	+	8,711,846	8,711,944	gp1b	middle
220	dre-miR-489_L-1	GTGACATCATATGTACGGCTGC	scaffold9.1	+	8,711,846	8,711,944	gp1b	middle
221	ipu-miR-499	TTAAGACTTGCAGTGATGTTT	scaffold11.1	+	2,482,179	2,482,270	gp1b	high
222	ola-miR-499_R+1	AACATCACTTTAAGTCTGTGCT	scaffold11.1	+	2,482,179	2,482,270	gp1b	middle
223	ccr-mir-722-p5	TTTGAAACGTTTTAGCCAAAA	scaffold82.1	+	923,657	923,750	gp1b	middle
224	dre-miR-722_L-2	TTTTGCAGAAACGTTTCAGATT	scaffold82.1	+	923,657	923,750	gp1b	middle
225	dre-miR-724_L-1	TAAAGGGAATTTGCGACTGTT	scaffold56.1	-	2,792,183	2,792,267	gp1b	middle
226	dre-miR-727-5p_1ss11AG	TCAGTCTTCAGTTCCTCCCAGC	scaffold32.1	+	4,670,644	4,670,737	gp1b	middle
227	dre-miR-727-3p_1ss22AC	GTTGAGGCGAGTTGAAGACTTC	scaffold32.1	+	4,670,644	4,670,737	gp1b	middle
228	hhi-mir-728-p5	AAATGTAGTAGACTTTAAGTATAC	scaffold58.1	+	3,303,018	3,303,099	gp1b	middle
229	hhi-miR-728_R+1	ATACTAAGTACACTACGTTTAT	scaffold58.1	+	3,303,018	3,303,099	gp1b	middle
230	hhi-mir-737-p5_1ss2TG	GGTTTTTTAGGTTTTGATTTTT	scaffold151.1	-	2,706,049	2,706,118	gp1b	middle
231	ssc-mir-1285-p5_1ss23CA	GTGGGATCGCGCCTGTGAATAGAC	scaffold14.1	-	4,803,175	4,803,249	gp1b	middle
232	dre-miR-1306_R-1	CCACCTCCCCTGCAAACGTCC	scaffold2.1	-	6,610,229	6,610,345	gp1b	middle
233	ipu-miR-1388_R-1	ATCTCAGGTTCGTCAGCCCATG	scaffold33.1	-	4,635,895	4,635,953	gp1b	middle
234	hhi-miR-1788	GGCTTGTTTTAAGTTGCCTGCG	scaffold8.1	-	5,426,238	5,426,339	gp1b	middle
235	hhi-mir-1788-p3	CAGGCAGCTAAAGCAAGTCT	scaffold8.1	-	5,426,238	5,426,339	gp1b	middle
236	dre-miR-2187-5p_R-1	TTAATTAGTATAGCCTGTTTT	scaffold13.12	+	114,583	114,680	gp1b	middle
237	bta-mir-3596-p5_1ss6CT	GAAGGTAGTAGGTTGTATAGTT	scaffold6.1	-	2,425,986	2,426,063	gp1b	middle
238	dre-miR-7147	TGTACCATGCTGGTAGCCAGT	scaffold71.1	+	979,907	979,988	gp1b	middle
239	hhi-mir-7641-p5	ACGCCCGATCTCGTCCGATCTCGGA	scaffold12.1	+	10,347,295	10,347,365	gp1b	high
240	hsa-mir-7641-2-p3	CTCGGAAGCTAAGCAGGGTCGGGC	scaffold12.1	+	10,347,295	10,347,365	gp1b	high
