# Supplementary material for: The integrated analysis of RNA-seq and microRNA-seq depicts miRNA-mRNA networks involved in Japanese flounder (Paralichthys olivaceus) albinism
Source: PLoS One. 2017 Aug 4;12(8):e0181761. doi: 10.1371/journal.pone.0181761 (PMC5544202; doi:10.1371/journal.pone.0181761)
Supplement: S11 Table — (PDF) [file pone.0181761.s015.pdf]

S11 Table. 75 unique miRNAs mapping to selected species (except for Japanese flounder) miRNAs/pre-miRNAs in miRbase, while only miRNA not pre-miRNAs mapping to the genome.

miRNA_Index	miR_name	miR_seq	genomeID	strand	start	end	group	Expression level
1	dre-miR-10a-5p	TACCCTGTAGATCCGAATTTGT	scaffold27.1	-	609,033	609,116	gp2	middle
2	dre-miR-16a_R-1	TAGCAGCACGTAAATATTGGT	scaffold12.1	+	7,353,140	7,353,226	gp2	middle
3	ola-mir-19a-p5_1ss17AG	AGTTTTGCATAGTTGCGCTGC	scaffold50.1	-	2,082,762	2,082,848	gp2	middle
4	dre-miR-20a-5p_R-1_1ss21TC	TAAAGTGCTTATAGTGCAGGCA	scaffold145.1	+	644,216	644,321	gp2	middle
5	dre-miR-20a-3p_2ss7GA11GA	ACTGCAATGTAAGCACTTGAAG	scaffold145.1	+	644,216	644,321	gp2	middle
6	dre-miR-21_1ss23CA	TAGCTTATCAGACTGGTGTTGGA	scaffold10.1	+	9,156,697	9,156,788	gp2	middle
7	ola-miR-22_R+1_1ss11CT	AAGCTGCCAGTTGAAGAACTGT	scaffold12.1	-	10,545,557	10,545,672	gp2	middle
8	pma-miR-23a-5p_L-2	GGGTTCCTGGCACGGTGATTT	scaffold22.1	-	245,606	245,735	gp2	middle
9	oan-miR-23b-3p_R+2_1ss18AT	ATCACATTGCCAGGGATTACCACT	scaffold22.1	-	245,606	245,735	gp2	middle
10	fru-mir-23a-3-p5	GGATTCCTGGCAGAGTGATTTT	scaffold44.1	-	1,750,802	1,750,924	gp2	middle
11	fru-miR-24-5p_L-1	TGCCTACTGAACTGGTATCAGT	scaffold44.1	-	3,007,332	3,007,416	gp2	middle
12	dre-miR-24_1ss21AC	TGGCTCAGTTCAGCAGGAACCG	scaffold90.1	+	688,365	688,454	gp2	middle
13	dre-miR-26b	TTCAAGTAATCCAGGATAGGTT	scaffold62.1	-	2,247,988	2,248,081	gp2	middle
14	hsa-miR-26a-2-3p_R+1	CCTATTCTTGATTACTTGTTTCT	scaffold62.1	-	2,247,988	2,248,081	gp2	middle
15	dre-miR-26a-5p_R-1_1ss21CT	TTCAAGTAATCCAGGATAGGT	scaffold32.1	-	5,486,567	5,486,681	gp2	middle
16	ola-miR-27c-3p_R+3	TTCACAGTGGTTAAGTTCTGCCA	328,521,904	+	503	635	gp2	middle
17	hsa-miR-27a-3p_R+1	TTCACAGTGGCTAAGTTCCGCG	scaffold44.1	-	1,749,934	1,750,014	gp2	middle
18	dre-miR-27e_1ss22GA	TTCACAGTGGCTAAGTTCAGTA	scaffold44.1	-	3,007,696	3,007,823	gp2	middle
19	dre-miR-27d_R-1	TTCACAGTGGCTAAGTTCTTC	scaffold54.1	-	2,162,907	2,163,018	gp2	middle
20	hsa-miR-29b-3p_R-1_1ss10TA	TAGCACCATATGAAATCAGTGT	scaffold6.1	-	860,709	860,815	gp2	middle
21	ola-miR-30d-5p_R+1_1ss12CT	TGTAAACATCCTCGACTGGAAGCT	scaffold7.1	-	4,318,621	4,318,725	gp2	middle
22	ola-miR-30a-3p_R+4	CTTTCAGTCGGATGTTTGCAGCT	scaffold7.1	-	4,318,621	4,318,725	gp2	middle
23	dre-miR-30a-5p_R-2_2ss10TC12C	TGTAAACATCCTCGACTGGA	scaffold9.1	+	3,842,729	3,842,840	gp2	middle
24	dre-miR-30a-3p	CTTTCAGTCTGATGTTTGCTGC	scaffold9.1	+	3,842,729	3,842,840	gp2	middle
25	ola-miR-30c_1ss21AG	TGTAAACATCCTACACTCTCGGC	scaffold7.1	-	4,316,464	4,316,588	gp2	middle
26	dre-miR-31_L+1	AGGCAAGATGTTGGCATAGCTG	scaffold38.1	+	683,764	683,841	gp2	middle
27	dre-miR-92a-5p_2ss5TG12GA	AGGTGGGGATTAGTAGCAATGCT	scaffold145.1	+	644,543	644,648	gp2	middle

28	dre-miR-92a-3p_R+1	TATTGCACTTGTCCCGGCCTGTT	scaffold145.1	+	644,543	644,648	gp2	high
29	dre-miR-93_R+1	AAAAGTGCTGTTTGTGCAGGTAG	scaffold46.1	+	1,400,949	1,401,037	gp2	middle
30	dre-miR-99_R+1_1	AACCCGTAGATCCGATCTTGTGA	scaffold10.1	+	10,697,194	10,697,290	gp2	high
31	dre-miR-99_R+1_2	AACCCGTAGATCCGATCTTGTGT	scaffold190.1	+	665,314	665,374	gp2	high
32	dre-miR-125b-5p_R+1	TCCCTGAGACCCTAACTTGTGAT	scaffold10.1	+	10,705,652	10,705,738	gp2	middle
33	aca-miR-129b-3p_R+2_1ss20CT	AAGCCCTTACCCCAAAAAGTATT	scaffold6.1	+	3,602,555	3,602,661	gp2	middle
34	dre-miR-133c-3p_L-1R+2_1ss10T	TTGGTCCCCTTCAACCAGCTATT	scaffold14.1	+	5,270,985	5,271,101	gp2	high
35	dre-miR-133c-3p_R+1_1ss10TC	TTTGGTCCCCTTCAACCAGCTAT	scaffold37.1	-	1,903,986	1,904,075	gp2	high
36	dre-miR-133c-3p_2ss10TC22AT	TTTGGTCCCCTTCAACCAGCTT	scaffold44.1	-	1,458,735	1,458,842	gp2	middle
37	oan-miR-139-3p_R-1_1ss8AC	TGGAGACCCAGCTCTGTTGGA	scaffold18.1	-	5,201,399	5,201,492	gp2	middle
38	dre-miR-145-5p_L+1	GGTCCAGTTTTCCCAGGAATCCC	scaffold53.12	-	598,600	598,691	gp2	middle
39	dre-miR-145-3p_L-2R+2	ATTCCTGGAAATACTGTTCTTT	scaffold53.12	-	598,600	598,691	gp2	middle
40	dre-miR-146a	TGAGAACTGAATTCCATAGATGG	scaffold16.1	-	3,774,363	3,774,468	gp2	high
41	ola-mir-146a-p3_1ss12CT	ATCTATGGGCTTAGTTCTTTTG	scaffold16.1	-	3,774,363	3,774,468	gp2	middle
42	ola-miR-150_R+2	ACTCCCAATCCTTGTACCAGTGT	scaffold180.1	-	299,366	299,475	gp2	middle
43	ipu-miR-152_R+1_2ss9TC10GT	TCAGTGCACTACAGAACTTTGT	scaffold50.1	+	1,403,363	1,403,487	gp2	middle
44	dre-miR-181c-5p_R+1_1ss10TC	CACATTCATCGCTGTCGGTGGGT	scaffold27.1	-	39,357	39,441	gp2	middle
45	dre-miR-181a-2-3p_2ss16CT20AC	ACCATCGACCGTTGATTGTGCC	scaffold32.1	-	353,327	353,434	gp2	middle
46	mdo-miR-187-3p_R+3	TCGTGTCTTGTGTTGCAGCCAGA	scaffold9.1	-	9,650,845	9,650,922	gp2	middle
47	dre-miR-194a_R+1	TGTAACAGCAACTCCATGTGGA	scaffold10.1	+	7,345,368	7,345,461	gp2	middle
48	ola-miR-199a-5p_R+1_1ss11AG	CCCAGTGTTCGGACTACCTGTTC	scaffold32.1	-	4,298,851	4,298,944	gp2	middle
49	ola-miR-199a-3p_R-1_1ss20TC	ACAGTAGTCTGCACATTGGCT	scaffold32.1	-	4,298,851	4,298,944	gp2	middle
50	ola-miR-199a-5p_1ss22TC	CCCAGTGTTCAGACTACCTGTC	scaffold82.1	+	752,228	752,327	gp2	middle
51	dre-miR-200b-3p_R+2	TAATACTGCCTGGTAATGATGATT	scaffold19.1	+	2,364,518	2,364,609	gp2	middle
52	dre-miR-203b-3p_L-1R+2_1ss11C	TGAAATGTTTAGGACCACTTGAT	scaffold15.1	-	2,069,451	2,069,540	gp2	middle
53	dre-miR-204-5p_R+2	TTCCCTTTGTCATCCTATGCCTGT	scaffold2.1	-	1,829,758	1,829,901	gp2	middle
54	dre-miR-204-5p_L+1	TTTCCCTTTGTCATCCTATGCCT	scaffold52.1	-	2,330,483	2,330,569	gp2	middle
55	hsa-miR-205-5p_R+2	TCCTTCATTCCACCGGAGTCTGTT	scaffold41.1	-	2,874,490	2,874,591	gp2	middle
56	dre-miR-206-3p_L-2R+2	GAATGTAAGGAAGTGTGTGGCT	scaffold14.1	+	5,270,160	5,270,250	gp2	middle
57	dre-miR-222a-5p_R-1_1ss2GT	TTCTCAGTAGTCAGTGTAGATC	scaffold131.1	-	542,697	542,788	gp2	middle
58	dre-miR-222a-3p_R+2	AGCTACATCTGGCTACTGGGTCTC	scaffold131.1	-	542,697	542,788	gp2	middle
59	dre-miR-375_R+1	TTTGTTCGTTCGGCTCGCGTTAA	scaffold8.1	-	5,438,652	5,438,769	gp2	middle

60	dre-miR-458-5p_R-1_1ss11AT	AGCGCCATTTTCAGAGCTATA	scaffold143.1	+	794,813	794,901	gp2	middle
61	dre-miR-458-3p_1ss11GA	ATAGCTCTTTAAATGGTACTGC	scaffold143.1	+	794,813	794,901	gp2	middle
62	dre-miR-462_R+3	TAACGGAACCCATAATGCAGCTGT	scaffold19.1	+	3,953,867	3,953,948	gp2	middle
63	ipu-miR-499_R+2	TTAAGACTTGCAGTGATGTTTAA	scaffold11.1	+	2,482,184	2,482,272	gp2	middle
64	ipu-miR-551_R+1_1ss14GA	GCGACCCATCCTTAGTTTCTG	scaffold18.1	-	4,544,768	4,544,841	gp2	middle
65	dre-miR-725-3p_R-3_1ss17AG	TTCAGTCATTGTTTCTGGT	scaffold43.1	-	1,862,391	1,862,477	gp2	middle
66	dre-miR-736_R+1_2ss1GA21TG	ATAAGACGAACAAAAAGTTTGT	scaffold32.1	-	8,458,682	8,458,767	gp2	middle
67	dre-miR-2184	AACAGTAAGAGTTTATGTGCT	scaffold74.1	-	584,233	584,304	gp2	middle
68	dre-miR-2188-5p_R+1	AAGGTCCAACCTCACATGTCCT	scaffold94.1	-	2,151,877	2,152,027	gp2	high
69	dre-miR-2188-3p_L+1R-1_1ss13T	GCTGTGTGAGGTCAGACCTAT	scaffold94.1	-	2,151,877	2,152,027	gp2	middle
70	ccr-mir-7132-p5_1ss10TA	GACTTGGTCAAAGCTCCTCAGT	scaffold1.1	-	1,055,458	1,055,530	gp2	middle
71	ipu-mir-7552-p5	TTACAATTAAAGGATATTTCTT	scaffold123.1	-	2,480,286	2,480,377	gp2	middle
72	mmu-let-7c-5p_R+2_2	TGAGGTAGTAGGTTGTATGGTTTT	scaffold10.1	+	10,697,571	10,697,672	gp2	middle
73	hsa-let-7g-5p	TGAGGTAGTAGTTTGTACAGTT	scaffold100.1	-	494,808	494,909	gp2	middle
74	dre-let-7d-5p_L+1	CTGAGGTAGTTGGTTGTATGGTT	scaffold30.1	-	634,835	634,935	gp2	middle
75	hsa-let-7e-5p_2ss9GT18TA	TGAGGTAGTAGGTTGTAAAGTT	scaffold41.1	-	2,315,232	2,315,358	gp2	middle
