# Supplementary material for: The integrated analysis of RNA-seq and microRNA-seq depicts miRNA-mRNA networks involved in Japanese flounder (Paralichthys olivaceus) albinism
Source: PLoS One. 2017 Aug 4;12(8):e0181761. doi: 10.1371/journal.pone.0181761 (PMC5544202; doi:10.1371/journal.pone.0181761)
Supplement: S12 Table — (PDF) [file pone.0181761.s016.pdf]

S12 Table. 65 unique miRNAs mapping to selected species (except for Japanese flounder) miRNAs/pre-miRNAs in miRbase, but both pre-miRNA and miRNA do not map to the genome.

miRNA_Index	miR_name	miR_seq	#mir/MIR	group	Expression level
1	pma-miR-1c-3p_1ss2GT	TTGAATGTAAAGAAGTATGTAC	5	gp3	middle
2	pma-miR-15a_R-1	TAGCAGCACGTAATGGTTTGT	20	gp3	middle
3	xtr-miR-15c_R-1_1ss21TA	TAGCAGCACATCATGGTTTGA	18	gp3	middle
4	oan-miR-16b-5p_1ss22GA	TAGCAGCACGTAAATATTGGTA	44	gp3	middle
5	ola-miR-19b_R+4_2	TGTGCAAATCCATGCAAAGCTC	31	gp3	middle
6	hsa-miR-21-5p	TAGCTTATCAGACTGATGTTGA	17	gp3	middle
7	cfa-miR-22_1ss21GT	AAGCTGCCAGTTGAAGAACTTT	15	gp3	middle
8	pma-miR-23b_R+1_1ss23CT	ATCACATTGCCAGGGATTACCATT	22	gp3	middle
9	ola-miR-24b-5p_1ss17AT	TGCCTACTGAGCTGATTACAGT	42	gp3	middle
10	ola-miR-24b-3p_R+5	TGGCTCAGTTCAGCAGGAACCGT	42	gp3	middle
11	aca-miR-26-5p_L+2R+2	TCTTCAAGTAATCCAGGATAGGCT	37	gp3	middle
12	dre-miR-27b-3p_R+1_1ss22AT	TTCACAGTGGCTAAGTTCTGCTT	28	gp3	middle
13	cgr-miR-29a-3p_R+1	TAGCACCATCTGAAATCGGTTT	27	gp3	middle
14	aca-miR-30a-3p_R+1_1ss22CT	CTTTCAGTCGGATGTTTGCAGTT	42	gp3	middle
15	tgu-miR-30e_R+1	TGTAAACATCCTACACTCAGCTA	39	gp3	middle
16	xtr-miR-92a_R+4	TATTGCACTTGTCCCGGCCTGTTTT	49	gp3	middle
17	mdo-miR-92a-3p_R+1_1ss22TA	TATTGCACTTGTCCCGGCCTGAT	5	gp3	middle
18	dre-miR-93_R+2	AAAAGTGCTGTTTGTGCAGGTATT	16	gp3	middle
19	ccr-miR-99_R+2_1ss20GT	AACCCGTAGATCCGATCTTTTGA	27	gp3	middle
20	hsa-miR-99b-5p	CACCCGTAGAACCGACCTTGCG	3	gp3	middle
21	ola-miR-106a_R+2	TAAAGTGCTTACAGTGCAGGTAG	42	gp3	middle
22	bta-miR-125b_R+1_1ss22AT	TCCCTGAGACCCTAACTTGTGTT	42	gp3	middle
23	cgr-miR-125a-5p_R-2	TCCCTGAGACCCTTTAACCTGT	7	gp3	middle
24	rno-miR-126a-3p_L-1R+1_1ss22GC	CGTACCGTGAGTAATAATGCCT	21	gp3	middle
25	xtr-miR-128_R+2	TCACAGTGAACCGGTCTCTTTTAT	23	gp3	middle
26	hsa-miR-130b-5p_R+1	ACTCTTTCCCTGTTGCACTACT	34	gp3	middle
27	xtr-miR-133b_L+1R+1_1ss2TG	TGTGGTCCCCTTCAACCAGCTAT	19	gp3	middle

28	dre-miR-141-3p	TAACACTGTCTGGTAACGATGC	33	gp3	middle
29	ola-miR-146a-5p_R-1_1ss2GT	TTAGAACTGAATTCCATAGATGGT	20	gp3	middle
30	cfa-miR-146a	TGAGAACTGAATTCCATGGGTT	17	gp3	middle
31	bta-miR-150_R-1	TCTCCCAACCCTTGTACCAGTG	14	gp3	middle
32	pma-miR-181a-5p_L+3	ATCAACATTCAACGCTGTCGGTGAGT	22	gp3	middle
33	pma-miR-181a-3p_R+2_1ss20AG	ACCATCGACCGTTGACTGTGCCTT	22	gp3	middle
34	mdo-miR-181b-5p_R+3	AACATTCATTGCTGTCGGTGGGTTGT	19	gp3	middle
35	aca-miR-191-5p_R-1	CAACGGAATCCCAAAAGCAGCT	14	gp3	middle
36	mmu-miR-195a-5p_R+1	TAGCAGCACAGAAATATTGGCA	14	gp3	middle
37	pma-miR-196a-5p_L+3	ATCTAGGTAGTTTCATGTTGTTGGG	21	gp3	middle
38	pma-miR-199a-5p_L+3	ATCCCCAGTGTTCAGACTACCTGTTC	32	gp3	middle
39	pma-miR-199b-3p_L+1_2ss20TG22A	ACAGTAGTCTGCACATTGGGTT	32	gp3	middle
40	aca-miR-200b-3p_R+3	TAATACTGCCTGGTAATGATGATTT	23	gp3	middle
41	ccr-miR-203a_L-1R+2	TGAAATGTTTAGGACCACTTGTT	15	gp3	middle
42	pma-miR-204-5p_R+2	TTCCCTTTGTCATCCTATGCCTTT	16	gp3	middle
43	oan-miR-205-5p_R+5	TCCTTCATTCCACCGGAGTCTGTTTT	26	gp3	middle
44	ssc-miR-206_1ss2GT	TTGAATGTAAGGAAGTGTGTGA	9	gp3	middle
45	mdo-miR-210-3p_L-1R+3	CTGTGCGTGTGACAGCGGCTACT	15	gp3	middle
46	tgu-miR-214-3p_L+1R+2	TACAGCAGGCACAGACAGGCAGTAG	3	gp3	middle
47	ola-miR-214_L-1R+8	TACAGCAGGCACAGACAGGCAGAAAA	1	gp3	middle
48	bta-miR-218_R+2	TTGTGCTTGATCTAACCATGTGTT	36	gp3	middle
49	pma-miR-221	AGCTACATTGTCTGCTGGGTTC	21	gp3	middle
50	bta-miR-374a_R-1	TTATAATACAACCTGATAAGT	17	gp3	middle
51	ggo-miR-424	CAGCAGCAATTCATGTTTTGA	9	gp3	middle
52	cfa-miR-429_R+2	TAATACTGTCTGGTAATGCCGTTT	7	gp3	middle
53	hhi-miR-430b_R+1	TAAGTGCTTCTCTTTGGGGTTGT	6	gp3	middle
54	gga-miR-456-3p_R+1_1ss22AT	CAGGCTGGTTAGATGGTTGTCTT	6	gp3	middle
55	mdo-miR-499-5p_R+3	TTAAGACTTGCAGTGATGTTTATT	16	gp3	middle
56	oan-miR-1386_L+2	GACTCCTGGCTGGCTCGCCA	1	gp3	middle
57	gga-miR-2188-5p_R+3	AAGGTCCAACCTCACATGTCCTTTA	5	gp3	middle
58	bta-miR-2478_L+2	TCGTATCCCACTTCTGACACCA	1	gp3	middle
59	hsa-miR-4286_R+1	ACCCCACTCCTGGTACCA	3	gp3	middle

60	hsa-miR-4454_L-2_1ss8AG	ATCCGGGTCACGGCACCA	1	gp3	middle
61	mmu-miR-6240_L-1R-1_1ss25CT	CAAAGCATCGCGAAGGCCCACGGT	2	gp3	middle
62	hsa-miR-7977_1ss6AG	TTCCCGGCCAACGCACCA	1	gp3	middle
63	mmu-let-7c-5p_R+2_1	TGAGGTAGTAGGTTGTATGGTTAT	89	gp3	middle
64	tni-let-7j_1ss2GT	TTAGGTAGTTTTTTGTACAGTT	15	gp3	middle
65	hsa-let-7e-5p_1ss6TG	TGAGGGAGGAGGTTGTATAGTT	8	gp3	middle
