# Supplementary material for: The integrated analysis of RNA-seq and microRNA-seq depicts miRNA-mRNA networks involved in Japanese flounder (Paralichthys olivaceus) albinism
Source: PLoS One. 2017 Aug 4;12(8):e0181761. doi: 10.1371/journal.pone.0181761 (PMC5544202; doi:10.1371/journal.pone.0181761)
Supplement: S13 Table — (PDF) [file pone.0181761.s017.pdf]

S13 Table. 64 novel unique miRNAs which do not map to selected pre-miRNAs in miRbase, but the reads map to genome & the extended genome sequences from genome may form hairpins.

miRNA_Index	miR_name	miR_seq	genomeID	strand	start	end	Sequence in miRbase	group	Expression level
1	PC-5p-49538_17	GAACTATTCTGCAACATTTGTT	scaffold8.1	+	3,440,394	3,440,477	New	gp4	middle
2	PC-3p-27631_54	TAAATGCTGCAGAATTGTGCTC	scaffold8.1	+	3,440,394	3,440,477	New	gp4	middle
3	PC-5p-16384_114	TCAGTCTTCAGTTCCTCCCAGCT	scaffold58.1	+	3,301,509	3,301,605	New	gp4	middle
4	PC-3p-34584_37	TTGAGGCGAGTTGAAGACTTCT	scaffold58.1	+	3,301,509	3,301,605	New	gp4	middle
5	PC-5p-74680_7	GATCCTGTAGAGAAGACATTTTGTTT	86,201,401	+	419	571	New	gp4	middle
6	PC-3p-237956_2	TCTGTAAAGTGTAAAAAATTGACT	86,201,401	+	419	571	New	gp4	middle
7	PC-5p-53714_14	TCATGCACCCCTCTGGAGAATGT	scaffold113.1	+	263,936	264,026	New	gp4	middle
8	PC-3p-56229_13	TTATTCAGGGTTCGGTGCATGTTT	scaffold113.1	+	263,936	264,026	New	gp4	middle
9	PC-5p-20206_87	TTCAGGTCCTGGTCTCAGTACT	scaffold151.1	-	2,643,968	2,644,106	New	gp4	middle
10	PC-3p-36824_33	TTCAGATCCTGGTCTCAGTACT	scaffold151.1	-	2,643,968	2,644,106	New	gp4	middle
11	PC-5p-1227_1088	AGCTAGAAACTTTGCCTGGGAAT	scaffold43.1	-	1,862,388	1,862,497	New	gp4	middle
12	PC-3p-53_52086	TTCAGTCATTGTTTCTGGTCGT	scaffold43.1	-	1,862,388	1,862,497	New	gp4	high
13	PC-5p-59688_11	TTCAAGTAATCCAGGATAGGTAT	scaffold49.1	+	2,642,987	2,643,088	New	gp4	middle
14	PC-3p-13617_140	CCTATTCTGGATGACTTGGGTC	scaffold49.1	+	2,642,987	2,643,088	New	gp4	middle
15	PC-5p-16752_111	GAAACCAAGTGTGGGTGTGGCCT	scaffold18.1	-	4,544,767	4,544,840	New	gp4	middle
16	PC-3p-654_1952	GCGACCCATCCTTAGTTTCTGT	scaffold18.1	-	4,544,767	4,544,840	New	gp4	middle
17	PC-3p-25255_63	TGTTGCTGTCACATGGTAAATT	scaffold11.1	+	2,488,703	2,488,812	New	gp4	middle
18	PC-5p-69891_8	GACCTGCTGGAGCTACTGGCCCATC	scaffold9.1	-	8,599,989	8,600,092	New	gp4	middle
19	PC-3p-264971_2	GGTGCTGTTGGCCCCGTTGGTCCCGC	scaffold9.1	-	8,599,989	8,600,092	New	gp4	middle
20	PC-5p-27999_53	CAGGACTTGACCCACATGTGAAA	328,521,904	+	497	624	New	gp4	middle
21	PC-3p-6752_280	TTACATTTGTTTTCTTGGCATG	scaffold220.1	+	170,623	170,712	New	gp4	middle
22	PC-3p-38603_30	TTCAGTTGCTGCGGACATTCT	scaffold52.1	-	1,601,989	1,602,098	New	gp4	middle
23	PC-3p-55961_13	CCTGGACCTGGATTCCTCATGC	scaffold60.1	+	2,037,655	2,037,794	New	gp4	middle
24	PC-5p-31696_43	TAGGTAACCGGACTTGCTTGTT	scaffold4.1	-	8,810,375	8,810,491	New	gp4	middle
25	PC-5p-24589_65	CAGCTTTTTGTTTGTGTTATTT	scaffold32.1	-	8,458,750	8,458,835	New	gp4	middle

26	PC-5p-169817_2	CTATGACTCTCTTAATGTAGC	scaffold25.1	-	5,078,542	5,078,699	New	gp4	middle
27	PC-5p-42145_25	CTCCAGTGTACCCAGATTTTGA	scaffold6.1	-	5,845,815	5,845,902	New	gp4	middle
28	PC-5p-43206_23	TGAGCTTCTGTCTTTCTCTCATT	scaffold125.1	+	646,882	646,978	New	gp4	middle
29	PC-3p-5079_357	CTGGGAGAGGGGTGTTTACGCT	scaffold9.1	+	10,248,971	10,249,060	New	gp4	middle
30	PC-3p-35576_35	TCCTGTCTGTTGGTCAGTAGGTC	scaffold62.1	-	2,492,053	2,492,157	New	gp4	middle
31	PC-3p-7808_245	ATCTATGGGCTTAGTTCTTTTGT	scaffold16.1	-	3,774,283	3,774,388	New	gp4	middle
32	PC-3p-26319_59	CAATGTGTCTGCAGTGCAGTAT	scaffold110.1	-	2,144,983	2,145,123	New	gp4	middle
33	PC-5p-46961_19	CCCCTTTTCTCTGCCGTCGTCCC	scaffold7.1	-	3,076,113	3,076,221	New	gp4	middle
34	PC-3p-42021_25	TGCTATGCCATCAAATTGTCATC	scaffold38.1	+	683,764	683,841	New	gp4	middle
35	PC-5p-37503_31	TCGTCAGCTGCGAGTCGACCT	scaffold136.1	+	178,245	178,355	New	gp4	middle
36	PC-3p-89852_5	TCTGAAACGTTTCCTACCGGAGT	scaffold94.1	-	2,150,708	2,150,809	New	gp4	middle
37	PC-5p-15232_124	ACTGGTTTCAGATGGTGTCTTAGA	scaffold41.1	+	3,009,498	3,009,619	New	gp4	middle
38	PC-3p-45646_21	TGCTCCGTGATTGGCTACTTGC	scaffold98.1	-	1,977,679	1,977,767	New	gp4	middle
39	PC-3p-51633_16	ACTGCAAAACCAGCACTTCAGG	scaffold46.1	+	1,400,920	1,401,054	New	gp4	middle
40	PC-5p-59593_11	GGACCCGTAGATCCGAACTTGT	scaffold190.1	+	665,277	665,406	New	gp4	middle
41	PC-5p-1022_1281	AGGACTTGGCTCGCTCCGTGA	scaffold44.1	-	1,749,999	1,750,113	New	gp4	middle
42	PC-3p-5807_319	TATTGCACTTGTCCCGGCCTGTATT	scaffold50.1	-	2,082,282	2,082,372	New	gp4	middle
43	PC-5p-401_3759	GACTTGGTCAAAGCTCCTCAGC	scaffold1.1	-	1,055,458	1,055,530	New	gp4	middle
44	PC-3p-42954_24	TTCACGTGGCTCGAGTCTTCCT	scaffold163.1	+	563,556	563,654	New	gp4	middle
45	PC-3p-28057_53	AGGGCGAAGCCAGAGGAA	scaffold60.1	+	2,872,352	2,872,413	New	gp4	middle
46	PC-5p-22736_74	AAATCAGATCCGTAACTTC	scaffold162.1	-	337,210	337,276	New	gp4	middle
47	PC-5p-50298_16	TTTCCCTGCTTAGCTTGAGACT	scaffold32.1	-	5,471,811	5,471,967	New	gp4	middle
48	PC-3p-44336_22	TGGCTCAGTCCAGCAGGAACAA	scaffold44.1	-	3,007,244	3,007,355	New	gp4	middle
49	PC-5p-81051_6	CGGGGCCGGGACGCTGTCTGAGAA	scaffold203.1	-	226,620	226,758	New	gp4	middle
50	PC-3p-39711_28	CCCGAGCTGCTGCGATCACCGCT	scaffold151.1	-	2,772,641	2,772,734	New	gp4	middle
51	PC-5p-43190_23	TAACAGAACACACAACTTACTG	scaffold1.1	-	9,119,362	9,119,514	New	gp4	middle
52	PC-5p-62148_10	TGTAACAGCATCTCCATATGGA	scaffold5.1	-	2,110,770	2,110,852	New	gp4	middle
53	PC-5p-28653_51	TACCCTTCCAGCTGAAATCTG	scaffold110.1	+	1,947,550	1,947,656	New	gp4	middle
54	PC-5p-72016_8	CGTCTTACCTTACAGTGCTGGA	scaffold19.1	+	2,364,694	2,364,777	New	gp4	middle
55	PC-5p-45747_20	TCTCCGGCTCCTGAGATGAAC	scaffold42.1	+	520,443	520,499	New	gp4	middle
56	PC-5p-14199_134	TCTAATCGCTGTGCTGAAGTGT	scaffold57.1	+	1,098,908	1,099,027	New	gp4	middle
57	PC-5p-66778_9	ACTGTTGTTCCTCTTTGGTTGC	scaffold92.1	-	2,085,183	2,085,261	New	gp4	middle

58	PC-3p-43498_23	CTATACAACTTACTGCCTTCCTA	scaffold100.1	-	494,406	494,528	New	gp4	middle
59	PC-5p-42988_24	ATTGCACAGTATGTGCTCTGGTCCT	scaffold192.1	+	333,011	333,111	New	gp4	middle
60	PC-3p-38354_30	GATTTCAGTGGTGTGAAGAGTAT	scaffold41.1	-	2,874,406	2,874,524	New	gp4	middle
61	PC-3p-52560_15	TAACTCGTCGCTCTGTCCTCCCC	scaffold56.1	-	990,434	990,542	New	gp4	middle
62	PC-5p-14246_134	AGAGCTTAGTCGACTGCTGAAC	scaffold54.1	-	2,162,976	2,163,111	New	gp4	middle
63	PC-5p-26963_57	TATGGATCATGAGTGATGTTCT	scaffold146.1	-	153,809	153,946	New	gp4	middle
64	PC-3p-1069_1227	ACCATCGACCGTTAAATGTACC	scaffold53.11	+	1,278,385	1,278,487	New	gp4	middle
