# Supplementary material for: The integrated analysis of RNA-seq and microRNA-seq depicts miRNA-mRNA networks involved in Japanese flounder (Paralichthys olivaceus) albinism
Source: PLoS One. 2017 Aug 4;12(8):e0181761. doi: 10.1371/journal.pone.0181761 (PMC5544202; doi:10.1371/journal.pone.0181761)
Supplement: S14 Table — (PDF) [file pone.0181761.s018.pdf]

S14 Table. The significant enrichment of GO terms for target genes of miRNAs (p≤0.001).

GO ID	Function	GO_term	S gene number	TS gene number	B gene number	TB gene number	Pvalue of Fisher's Exact Test
GO:0043565	molecular_function	sequence-specific DNA binding	398	10,676	966	41,389	0.00E+00
GO:0005739	cellular_component	mitochondrion	302	10,676	734	41,389	0.00E+00
GO:0003700	molecular_function	sequence-specific DNA binding transcription factor activity	514	10,676	1,349	41,389	0.00E+00
GO:0003735	molecular_function	structural constituent of ribosome	100	10,676	145	41,389	1.40E-27
GO:0006412	biological_process	translation	96	10,676	147	41,389	8.79E-24
GO:0009055	molecular_function	electron carrier activity	90	10,676	139	41,389	5.31E-22
GO:0005743	cellular_component	mitochondrial inner membrane	129	10,676	234	41,389	1.32E-21
GO:0004984	molecular_function	olfactory receptor activity	43	10,676	48	41,389	1.88E-20
GO:0005840	cellular_component	ribosome	77	10,676	118	41,389	2.44E-19
GO:0006364	biological_process	rRNA processing	48	10,676	61	41,389	7.94E-18
GO:0005179	molecular_function	hormone activity	55	10,676	77	41,389	6.62E-17
GO:0003723	molecular_function	RNA binding	351	10,676	898	41,389	2.22E-16
GO:0030170	molecular_function	pyridoxal phosphate binding	46	10,676	61	41,389	7.26E-16
GO:0005634	cellular_component	nucleus	1,982	10,676	6,451	41,389	7.77E-16
GO:0005730	cellular_component	nucleolus	206	10,676	490	41,389	2.33E-15
GO:0003677	molecular_function	DNA binding	761	10,676	2,316	41,389	3.22E-15
GO:0022900	biological_process	electron transport chain	55	10,676	83	41,389	1.16E-14
GO:0016338	biological_process	calcium-independent cell-cell adhesion via plasma membrane cell-adhesion molecules	37	10,676	49	41,389	4.68E-13
GO:0005576	cellular_component	extracellular region	440	10,676	1,283	41,389	4.08E-12
GO:0003924	molecular_function	GTPase activity	137	10,676	318	41,389	1.45E-11
GO:0005922	cellular_component	connexon complex	32	10,676	43	41,389	3.50E-11
GO:0016787	molecular_function	hydrolase activity	95	10,676	201	41,389	4.40E-11
GO:0006096	biological_process	glycolytic process	42	10,676	65	41,389	5.38E-11
GO:0020037	molecular_function	heme binding	79	10,676	158	41,389	6.02E-11
GO:0008168	molecular_function	methyltransferase activity	43	10,676	68	41,389	9.03E-11
GO:0016491	molecular_function	oxidoreductase activity	62	10,676	116	41,389	2.04E-10
GO:0051537	molecular_function	2 iron, 2 sulfur cluster binding	20	10,676	22	41,389	2.21E-10

GO:0006397	biological_process	mRNA processing	144	10,676	353	41,389	4.84E-10
GO:0015020	molecular_function	glucuronosyltransferase activity	17	10,676	18	41,389	1.34E-09
GO:0070936	biological_process	protein K48-linked ubiquitination	30	10,676	43	41,389	1.92E-09
GO:0006457	biological_process	protein folding	81	10,676	173	41,389	1.98E-09
GO:0005741	cellular_component	mitochondrial outer membrane	63	10,676	127	41,389	7.40E-09
GO:0005681	cellular_component	spliceosomal complex	45	10,676	82	41,389	2.04E-08
GO:0008380	biological_process	RNA splicing	106	10,676	254	41,389	2.15E-08
GO:0016706	molecular_function	[illegible] incorporation or reduction of molecular oxygen, 2-oxoglutarate as one donor, and incorporation of one atom each of oxygen into both donors	13	10,676	13	41,389	2.23E-08
GO:0031902	cellular_component	late endosome membrane	38	10,676	66	41,389	4.50E-08
GO:0055038	cellular_component	recycling endosome membrane	25	10,676	36	41,389	5.04E-08
GO:0005328	molecular_function	neurotransmitter:sodium symporter activity	29	10,676	45	41,389	5.63E-08
GO:0031625	molecular_function	ubiquitin protein ligase binding	37	10,676	64	41,389	5.82E-08
GO:0008378	molecular_function	galactosyltransferase activity	19	10,676	24	41,389	6.82E-08
GO:0005212	molecular_function	structural constituent of eye lens	28	10,676	43	41,389	6.86E-08
GO:0008270	molecular_function	zinc ion binding	1,083	10,676	3,680	41,389	1.02E-07
GO:0000932	cellular_component	cytoplasmic mRNA processing body	32	10,676	53	41,389	1.12E-07
GO:0030433	biological_process	ER-associated ubiquitin-dependent protein catabolic process	15	10,676	17	41,389	1.16E-07
GO:0005215	molecular_function	transporter activity	73	10,676	163	41,389	1.17E-07
GO:0016301	molecular_function	kinase activity	16	10,676	19	41,389	1.61E-07
GO:0008233	molecular_function	peptidase activity	16	10,676	19	41,389	1.61E-07
GO:0005125	molecular_function	cytokine activity	39	10,676	71	41,389	1.70E-07
GO:0006783	biological_process	heme biosynthetic process	13	10,676	14	41,389	2.37E-07
GO:0070403	molecular_function	NAD+ binding	11	10,676	11	41,389	3.35E-07
GO:0005845	cellular_component	mRNA cap binding complex	11	10,676	11	41,389	3.35E-07
GO:0004303	molecular_function	estradiol 17-beta-dehydrogenase activity	11	10,676	11	41,389	3.35E-07
GO:0003755	molecular_function	peptidyl-prolyl cis-trans isomerase activity	30	10,676	50	41,389	3.37E-07
GO:0005792	cellular_component	microsome	106	10,676	266	41,389	3.46E-07
GO:0006914	biological_process	autophagy	22	10,676	32	41,389	4.29E-07
GO:0015031	biological_process	protein transport	286	10,676	860	41,389	5.10E-07
GO:0045087	biological_process	innate immune response	75	10,676	174	41,389	5.15E-07

GO:0005789	cellular_component	endoplasmic reticulum membrane	349	10,676	1,077	41,389	5.41E-07
GO:0019835	biological_process	cytolysis	20	10,676	28	41,389	5.52E-07
GO:0008137	molecular_function	NADH dehydrogenase (ubiquinone) activity	16	10,676	20	41,389	6.09E-07
GO:0019843	molecular_function	rRNA binding	18	10,676	24	41,389	6.38E-07
GO:0005758	cellular_component	mitochondrial intermembrane space	17	10,676	22	41,389	6.43E-07
GO:0007586	biological_process	digestion	24	10,676	37	41,389	6.65E-07
GO:0005759	cellular_component	mitochondrial matrix	83	10,676	199	41,389	7.01E-07
GO:0005654	cellular_component	nucleoplasm	186	10,676	527	41,389	7.49E-07
GO:0051287	molecular_function	NAD binding	33	10,676	59	41,389	8.54E-07
GO:0009982	molecular_function	pseudouridine synthase activity	12	10,676	13	41,389	8.56E-07
GO:0050662	molecular_function	coenzyme binding	12	10,676	13	41,389	8.56E-07
GO:0005507	molecular_function	copper ion binding	22	10,676	33	41,389	9.71E-07
GO:0030145	molecular_function	manganese ion binding	22	10,676	33	41,389	9.71E-07
GO:0000166	molecular_function	nucleotide binding	200	10,676	577	41,389	1.22E-06
GO:0001619	molecular_function	lysosphingolipid and lysophosphatidic acid receptor activity	10	10,676	10	41,389	1.30E-06
GO:0009411	biological_process	response to UV	14	10,676	17	41,389	1.71E-06
GO:0000398	biological_process	mRNA splicing, via spliceosome	34	10,676	63	41,389	1.78E-06
GO:0005975	biological_process	carbohydrate metabolic process	67	10,676	155	41,389	1.79E-06
GO:0006986	biological_process	response to unfolded protein	17	10,676	23	41,389	1.87E-06
GO:0005525	molecular_function	GTP binding	312	10,676	963	41,389	2.15E-06
GO:0030529	cellular_component	ribonucleoprotein complex	45	10,676	93	41,389	2.34E-06
GO:0004181	molecular_function	metallocarboxypeptidase activity	20	10,676	30	41,389	3.06E-06
GO:0001944	biological_process	vasculature development	11	10,676	12	41,389	3.07E-06
GO:0031227	cellular_component	intrinsic component of endoplasmic reticulum membrane	11	10,676	12	41,389	3.07E-06
GO:0032088	biological_process	negative regulation of NF-kappaB transcription factor activity	23	10,676	37	41,389	3.37E-06
GO:0008083	molecular_function	growth factor activity	87	10,676	218	41,389	3.43E-06
GO:0015991	biological_process	ATP hydrolysis coupled proton transport	25	10,676	42	41,389	3.93E-06
GO:0005000	molecular_function	vasopressin receptor activity	9	10,676	9	41,389	5.04E-06
GO:0008180	cellular_component	COP9 signalosome	9	10,676	9	41,389	5.04E-06
GO:0005811	cellular_component	lipid particle	14	10,676	18	41,389	5.86E-06
GO:0005814	cellular_component	centriole	25	10,676	43	41,389	7.09E-06
GO:0005778	cellular_component	peroxisomal membrane	32	10,676	61	41,389	7.88E-06

GO:0006888	biological_process	ER to Golgi vesicle-mediated transport	19	10,676	29	41,389	7.98E-06
GO:0043169	molecular_function	cation binding	33	10,676	64	41,389	9.26E-06
GO:0030308	biological_process	negative regulation of cell growth	33	10,676	64	41,389	9.26E-06
GO:0040008	biological_process	regulation of growth	33	10,676	64	41,389	9.26E-06
GO:0007602	biological_process	phototransduction	26	10,676	46	41,389	9.49E-06
GO:0006310	biological_process	DNA recombination	34	10,676	67	41,389	1.07E-05
GO:0008235	molecular_function	metalloexopeptidase activity	10	10,676	11	41,389	1.09E-05
GO:0009103	biological_process	lipopolysaccharide biosynthetic process	10	10,676	11	41,389	1.09E-05
GO:0016918	molecular_function	retinal binding	10	10,676	11	41,389	1.09E-05
GO:0045028	molecular_function	G-protein coupled purinergic nucleotide receptor activity	17	10,676	25	41,389	1.15E-05
GO:0034641	biological_process	cellular nitrogen compound metabolic process	16	10,676	23	41,389	1.35E-05
GO:0006071	biological_process	glycerol metabolic process	16	10,676	23	41,389	1.35E-05
GO:0005694	cellular_component	chromosome	47	10,676	104	41,389	1.45E-05
GO:0019841	molecular_function	retinol binding	11	10,676	13	41,389	1.53E-05
GO:0004983	molecular_function	neuropeptide Y receptor activity	11	10,676	13	41,389	1.53E-05
GO:0009058	biological_process	biosynthetic process	15	10,676	21	41,389	1.54E-05
GO:0070330	molecular_function	aromatase activity	15	10,676	21	41,389	1.54E-05
GO:0000922	cellular_component	spindle pole	44	10,676	96	41,389	1.74E-05
GO:0055072	biological_process	iron ion homeostasis	12	10,676	15	41,389	1.74E-05
GO:0004806	molecular_function	triglyceride lipase activity	13	10,676	17	41,389	1.78E-05
GO:0051536	molecular_function	iron-sulfur cluster binding	8	10,676	8	41,389	1.96E-05
GO:0001614	molecular_function	purinergic nucleotide receptor activity	8	10,676	8	41,389	1.96E-05
GO:0004982	molecular_function	N-formyl peptide receptor activity	8	10,676	8	41,389	1.96E-05
GO:0005777	cellular_component	peroxisome	36	10,676	74	41,389	1.99E-05
GO:0003729	molecular_function	mRNA binding	18	10,676	28	41,389	2.05E-05
GO:0004091	molecular_function	carboxylesterase activity	23	10,676	40	41,389	2.11E-05
GO:0005643	cellular_component	nuclear pore	30	10,676	58	41,389	2.16E-05
GO:0006631	biological_process	fatty acid metabolic process	34	10,676	69	41,389	2.39E-05
GO:0051781	biological_process	positive regulation of cell division	28	10,676	53	41,389	2.42E-05
GO:0018298	biological_process	protein-chromophore linkage	26	10,676	48	41,389	2.64E-05
GO:0000079	biological_process	regulation of cyclin-dependent protein serine/threonine kinase activity	22	10,676	38	41,389	2.75E-05

GO:0000082	biological_process	G1/S transition of mitotic cell cycle	24	10,676	43	41,389	2.77E-05
GO:0009968	biological_process	negative regulation of signal transduction	32	10,676	64	41,389	2.83E-05
GO:0031901	cellular_component	early endosome membrane	44	10,676	98	41,389	3.27E-05
GO:0006805	biological_process	xenobiotic metabolic process	23	10,676	41	41,389	3.64E-05
GO:0043066	biological_process	negative regulation of apoptotic process	52	10,676	122	41,389	3.85E-05
GO:0042446	biological_process	hormone biosynthetic process	9	10,676	10	41,389	3.87E-05
GO:0015355	molecular_function	secondary active monocarboxylate transmembrane transporter activity	9	10,676	10	41,389	3.87E-05
GO:0006626	biological_process	protein targeting to mitochondrion	9	10,676	10	41,389	3.87E-05
GO:0003810	molecular_function	protein-glutamine gamma-glutamyltransferase activity	9	10,676	10	41,389	3.87E-05
GO:0006183	biological_process	GTP biosynthetic process	9	10,676	10	41,389	3.87E-05
GO:0001522	biological_process	pseudouridine synthesis	9	10,676	10	41,389	3.87E-05
GO:0006298	biological_process	mismatch repair	9	10,676	10	41,389	3.87E-05
GO:0015250	molecular_function	water channel activity	9	10,676	10	41,389	3.87E-05
GO:0030968	biological_process	endoplasmic reticulum unfolded protein response	9	10,676	10	41,389	3.87E-05
GO:0055085	biological_process	transmembrane transport	101	10,676	275	41,389	3.93E-05
GO:0071013	cellular_component	catalytic step 2 spliceosome	18	10,676	29	41,389	4.10E-05
GO:0006144	biological_process	purine nucleobase metabolic process	14	10,676	20	41,389	4.30E-05
GO:0004527	molecular_function	exonuclease activity	13	10,676	18	41,389	4.88E-05
GO:0005801	cellular_component	cis-Golgi network	10	10,676	12	41,389	5.03E-05
GO:0009798	biological_process	axis specification	10	10,676	12	41,389	5.03E-05
GO:0008202	biological_process	steroid metabolic process	10	10,676	12	41,389	5.03E-05
GO:0032781	biological_process	positive regulation of ATPase activity	10	10,676	12	41,389	5.03E-05
GO:0007338	biological_process	single fertilization	10	10,676	12	41,389	5.03E-05
GO:0035098	cellular_component	ESC/E(Z) complex	10	10,676	12	41,389	5.03E-05
GO:0001541	biological_process	ovarian follicle development	11	10,676	14	41,389	5.44E-05
GO:0002474	biological_process	antigen processing and presentation of peptide antigen via MHC class I	16	10,676	25	41,389	6.48E-05
GO:0051289	biological_process	protein homotetramerization	16	10,676	25	41,389	6.48E-05
GO:0004931	molecular_function	extracellular ATP-gated cation channel activity	7	10,676	7	41,389	7.59E-05
GO:0030127	cellular_component	COPII vesicle coat	7	10,676	7	41,389	7.59E-05
GO:0010524	biological_process	positive regulation of calcium ion transport into cytosol	7	10,676	7	41,389	7.59E-05

GO:0033179	cellular_component	proton-transporting V-type ATPase, V0 domain	7	10,676	7	41,389	7.59E-05
GO:0045736	biological_process	negative regulation of cyclin-dependent protein serine/threonine kinase activity	7	10,676	7	41,389	7.59E-05
GO:0007093	biological_process	mitotic cell cycle checkpoint	7	10,676	7	41,389	7.59E-05
GO:0004176	molecular_function	ATP-dependent peptidase activity	7	10,676	7	41,389	7.59E-05
GO:0015467	molecular_function	activity	7	10,676	7	41,389	7.59E-05
GO:0030983	molecular_function	mismatched DNA binding	7	10,676	7	41,389	7.59E-05
GO:0051920	molecular_function	peroxiredoxin activity	7	10,676	7	41,389	7.59E-05
GO:0045454	biological_process	cell redox homeostasis	36	10,676	78	41,389	8.17E-05
GO:0004519	molecular_function	endonuclease activity	25	10,676	48	41,389	8.88E-05
GO:0030133	cellular_component	transport vesicle	23	10,676	43	41,389	9.90E-05
GO:0004252	molecular_function	serine-type endopeptidase activity	82	10,676	220	41,389	1.12E-04
GO:0004197	molecular_function	cysteine-type endopeptidase activity	27	10,676	54	41,389	1.17E-04
GO:0007623	biological_process	circadian rhythm	13	10,676	19	41,389	1.18E-04
GO:0006369	biological_process	termination of RNA polymerase II transcription	13	10,676	19	41,389	1.18E-04
GO:0015288	molecular_function	porin activity	8	10,676	9	41,389	1.36E-04
GO:0004415	molecular_function	hyalurononglucosaminidase activity	8	10,676	9	41,389	1.36E-04
GO:0006241	biological_process	CTP biosynthetic process	8	10,676	9	41,389	1.36E-04
GO:0005319	molecular_function	lipid transporter activity	8	10,676	9	41,389	1.36E-04
GO:0006968	biological_process	cellular defense response	8	10,676	9	41,389	1.36E-04
GO:0006839	biological_process	mitochondrial transport	8	10,676	9	41,389	1.36E-04
GO:0006228	biological_process	UTP biosynthetic process	8	10,676	9	41,389	1.36E-04
GO:0046934	molecular_function	phosphatidylinositol-4,5-bisphosphate 3-kinase activity	8	10,676	9	41,389	1.36E-04
GO:0004550	molecular_function	nucleoside diphosphate kinase activity	8	10,676	9	41,389	1.36E-04
GO:0033077	biological_process	T cell differentiation in thymus	12	10,676	17	41,389	1.38E-04
GO:0005506	molecular_function	iron ion binding	46	10,676	109	41,389	1.39E-04
GO:0004803	molecular_function	transposase activity	11	10,676	15	41,389	1.56E-04
GO:0004602	molecular_function	glutathione peroxidase activity	11	10,676	15	41,389	1.56E-04
GO:0008156	biological_process	negative regulation of DNA replication	9	10,676	11	41,389	1.64E-04
GO:0004089	molecular_function	carbonate dehydratase activity	9	10,676	11	41,389	1.64E-04
GO:0009168	biological_process	purine ribonucleoside monophosphate biosynthetic process	9	10,676	11	41,389	1.64E-04
GO:0008199	molecular_function	ferric iron binding	9	10,676	11	41,389	1.64E-04

GO:0008271	molecular_function	secondary active sulfate transmembrane transporter activity	9	10,676	11	41,389	1.64E-04
GO:0000421	cellular_component	autophagic vacuole membrane	9	10,676	11	41,389	1.64E-04
GO:0031124	biological_process	mRNA 3'-end processing	10	10,676	13	41,389	1.67E-04
GO:0050660	molecular_function	flavin adenine dinucleotide binding	42	10,676	98	41,389	1.77E-04
GO:0050821	biological_process	protein stabilization	24	10,676	47	41,389	1.82E-04
GO:0006313	biological_process	transposition, DNA-mediated	14	10,676	22	41,389	2.06E-04
GO:0006260	biological_process	DNA replication	62	10,676	160	41,389	2.14E-04
GO:0030176	cellular_component	integral component of endoplasmic reticulum membrane	30	10,676	64	41,389	2.21E-04
GO:0051258	biological_process	protein polymerization	16	10,676	27	41,389	2.39E-04
GO:0009881	molecular_function	photoreceptor activity	23	10,676	45	41,389	2.43E-04
GO:0007126	biological_process	meiotic nuclear division	23	10,676	45	41,389	2.43E-04
GO:0009331	cellular_component	glycerol-3-phosphate dehydrogenase complex	6	10,676	6	41,389	2.94E-04
GO:0006646	biological_process	phosphatidylethanolamine biosynthetic process	6	10,676	6	41,389	2.94E-04
GO:0007342	biological_process	fusion of sperm to egg plasma membrane	6	10,676	6	41,389	2.94E-04
GO:0000375	biological_process	RNA splicing, via transesterification reactions	6	10,676	6	41,389	2.94E-04
GO:0032784	biological_process	regulation of DNA-templated transcription, elongation	6	10,676	6	41,389	2.94E-04
GO:0051881	biological_process	regulation of mitochondrial membrane potential	6	10,676	6	41,389	2.94E-04
GO:0006271	biological_process	DNA strand elongation involved in DNA replication	6	10,676	6	41,389	2.94E-04
GO:0006536	biological_process	glutamate metabolic process	6	10,676	6	41,389	2.94E-04
GO:0045861	biological_process	negative regulation of proteolysis	6	10,676	6	41,389	2.94E-04
GO:0043249	biological_process	erythrocyte maturation	6	10,676	6	41,389	2.94E-04
GO:0008967	molecular_function	phosphoglycolate phosphatase activity	6	10,676	6	41,389	2.94E-04
GO:0006703	biological_process	estrogen biosynthetic process	6	10,676	6	41,389	2.94E-04
GO:0008276	molecular_function	protein methyltransferase activity	6	10,676	6	41,389	2.94E-04
GO:0070328	biological_process	triglyceride homeostasis	6	10,676	6	41,389	2.94E-04
GO:0006979	biological_process	response to oxidative stress	39	10,676	91	41,389	2.95E-04
GO:0040007	biological_process	growth	25	10,676	51	41,389	3.07E-04
GO:0031418	molecular_function	L-ascorbic acid binding	15	10,676	25	41,389	3.10E-04
GO:0010494	cellular_component	cytoplasmic stress granule	12	10,676	18	41,389	3.17E-04
GO:0003964	molecular_function	RNA-directed DNA polymerase activity	22	10,676	43	41,389	3.24E-04
GO:0006508	biological_process	proteolysis	265	10,676	856	41,389	3.49E-04
GO:0008406	biological_process	gonad development	11	10,676	16	41,389	3.83E-04

GO:0043507	biological_process	positive regulation of JUN kinase activity	11	10,676	16	41,389	3.83E-04
GO:0005528	molecular_function	FK506 binding	11	10,676	16	41,389	3.83E-04
GO:0006417	biological_process	regulation of translation	40	10,676	95	41,389	3.87E-04
GO:0009615	biological_process	response to virus	24	10,676	49	41,389	4.09E-04
GO:0046983	molecular_function	protein dimerization activity	58	10,676	151	41,389	4.37E-04
GO:0045579	biological_process	positive regulation of B cell differentiation	7	10,676	8	41,389	4.70E-04
GO:0004952	molecular_function	dopamine neurotransmitter receptor activity	7	10,676	8	41,389	4.70E-04
GO:0012507	cellular_component	ER to Golgi transport vesicle membrane	7	10,676	8	41,389	4.70E-04
GO:0016620	molecular_function	oxidoreductase activity, acting on the aldehyde or oxo group of donors, NAD or NADP as acceptor	7	10,676	8	41,389	4.70E-04
GO:0048038	molecular_function	quinone binding	7	10,676	8	41,389	4.70E-04
GO:0006297	biological_process	nucleotide-excision repair, DNA gap filling	7	10,676	8	41,389	4.70E-04
GO:0042761	biological_process	very long-chain fatty acid biosynthetic process	7	10,676	8	41,389	4.70E-04
GO:0000178	cellular_component	exosome (RNase complex)	7	10,676	8	41,389	4.70E-04
GO:0008121	molecular_function	ubiquinol-cytochrome-c reductase activity	7	10,676	8	41,389	4.70E-04
GO:0008308	molecular_function	voltage-gated anion channel activity	7	10,676	8	41,389	4.70E-04
GO:0016607	cellular_component	nuclear speck	71	10,676	193	41,389	4.79E-04
GO:0003746	molecular_function	translation elongation factor activity	22	10,676	44	41,389	4.91E-04
GO:0006103	biological_process	2-oxoglutarate metabolic process	9	10,676	12	41,389	5.04E-04
GO:0006468	biological_process	protein phosphorylation	27	10,676	58	41,389	5.15E-04
GO:0015078	molecular_function	hydrogen ion transmembrane transporter activity	13	10,676	21	41,389	5.16E-04
GO:0070469	cellular_component	respiratory chain	13	10,676	21	41,389	5.16E-04
GO:0000245	biological_process	spliceosomal complex assembly	8	10,676	10	41,389	5.24E-04
GO:0048662	biological_process	negative regulation of smooth muscle cell proliferation	8	10,676	10	41,389	5.24E-04
GO:0046930	cellular_component	pore complex	8	10,676	10	41,389	5.24E-04
GO:0060027	biological_process	convergent extension involved in gastrulation	8	10,676	10	41,389	5.24E-04
GO:0043488	biological_process	regulation of mRNA stability	8	10,676	10	41,389	5.24E-04
GO:0006913	biological_process	nucleocytoplasmic transport	8	10,676	10	41,389	5.24E-04
GO:0015935	cellular_component	small ribosomal subunit	8	10,676	10	41,389	5.24E-04
GO:0006749	biological_process	glutathione metabolic process	8	10,676	10	41,389	5.24E-04
GO:0017091	molecular_function	AU-rich element binding	8	10,676	10	41,389	5.24E-04
GO:0016829	molecular_function	lyase activity	8	10,676	10	41,389	5.24E-04

GO:0017046	molecular_function	peptide hormone binding	8	10,676	10	41,389	5.24E-04
GO:0006520	biological_process	cellular amino acid metabolic process	15	10,676	26	41,389	5.59E-04
GO:0006278	biological_process	RNA-dependent DNA replication	21	10,676	42	41,389	6.55E-04
GO:0006606	biological_process	protein import into nucleus	12	10,676	19	41,389	6.58E-04
GO:0008654	biological_process	phospholipid biosynthetic process	23	10,676	48	41,389	7.94E-04
GO:0007030	biological_process	Golgi organization	11	10,676	17	41,389	8.32E-04
GO:0043015	molecular_function	gamma-tubulin binding	11	10,676	17	41,389	8.32E-04
GO:0030902	biological_process	hindbrain development	17	10,676	32	41,389	8.80E-04
GO:0045768	biological_process	positive regulation of anti-apoptosis	15	10,676	27	41,389	9.58E-04
GO:0007498	biological_process	mesoderm development	13	10,676	22	41,389	9.63E-04
