# Supplementary material for: The integrated analysis of RNA-seq and microRNA-seq depicts miRNA-mRNA networks involved in Japanese flounder (Paralichthys olivaceus) albinism
Source: PLoS One. 2017 Aug 4;12(8):e0181761. doi: 10.1371/journal.pone.0181761 (PMC5544202; doi:10.1371/journal.pone.0181761)
Supplement: S15 Table — (PDF) [file pone.0181761.s019.pdf]

S15 Table.The significant enrichment of KEGG pathways for target genes of miRNAs (p≤0.001).

Pathway ID	Pathway description	S gene number	TS gene number	B gene number	TB gene number	Pvalue of Fisher's Exact
ko03010	Ribosome	74	4,507	108	19,272	2.61E-23
ko00350	Tyrosine metabolism	59	4,507	78	19,272	2.18E-22
ko00983	Drug metabolism - other enzymes	46	4,507	54	19,272	1.04E-21
ko00140	Steroid hormone biosynthesis	44	4,507	51	19,272	2.79E-21
ko00190	Oxidative phosphorylation	81	4,507	132	19,272	1.03E-20
ko00860	Porphyrin and chlorophyll metabolism	38	4,507	45	19,272	7.00E-18
ko00520	Amino sugar and nucleotide sugar metabolism	54	4,507	78	19,272	1.06E-17
ko03320	PPAR signaling pathway	80	4,507	142	19,272	2.50E-17
ko00100	Steroid biosynthesis	28	4,507	30	19,272	5.25E-16
ko00010	Glycolysis / Gluconeogenesis	66	4,507	112	19,272	7.09E-16
ko00480	Glutathione metabolism	49	4,507	72	19,272	1.02E-15
ko00982	Drug metabolism - cytochrome P450	37	4,507	48	19,272	5.39E-15
ko00240	Pyrimidine metabolism	93	4,507	189	19,272	7.44E-15
ko00380	Tryptophan metabolism	44	4,507	65	19,272	4.01E-14
ko04620	Toll-like receptor signaling pathway	80	4,507	158	19,272	7.67E-14
ko00980	Metabolism of xenobiotics by cytochrome P450	35	4,507	47	19,272	1.79E-13
ko00040	Pentose and glucuronate interconversions	24	4,507	27	19,272	9.36E-13
ko05012	Parkinson's disease	92	4,507	199	19,272	1.08E-12
ko00830	Retinol metabolism	50	4,507	84	19,272	1.32E-12
ko05140	Leishmaniasis	49	4,507	82	19,272	1.81E-12
ko00632	Benzoate degradation via CoA ligation	24	4,507	28	19,272	5.09E-12
ko04142	Lysosome	123	4,507	300	19,272	6.73E-12
ko04612	Antigen processing and presentation	36	4,507	54	19,272	1.67E-11
ko04744	Phototransduction	43	4,507	72	19,272	4.20E-11
ko04111	Cell cycle - yeast	66	4,507	134	19,272	5.45E-11
ko00051	Fructose and mannose metabolism	52	4,507	96	19,272	6.78E-11
ko04130	SNARE interactions in vesicular transport	37	4,507	60	19,272	2.60E-10
ko04146	Peroxisome	49	4,507	91	19,272	3.15E-10

ko00770	Pantothenate and CoA biosynthesis	18	4,507	20	19,272	4.92E-10
ko00030	Pentose phosphate pathway	31	4,507	48	19,272	1.41E-09
ko00590	Arachidonic acid metabolism	39	4,507	68	19,272	1.76E-09
ko00310	Lysine degradation	57	4,507	117	19,272	1.85E-09
ko00500	Starch and sucrose metabolism	49	4,507	96	19,272	3.48E-09
ko04140	Regulation of autophagy	20	4,507	25	19,272	3.52E-09
ko00624	1- and 2-Methylnaphthalene degradation	17	4,507	20	19,272	9.89E-09
ko00071	Fatty acid metabolism	40	4,507	75	19,272	1.88E-08
ko04740	Olfactory transduction	83	4,507	203	19,272	1.98E-08
ko04950	Maturity onset diabetes of the young	30	4,507	50	19,272	3.17E-08
ko05340	Primary immunodeficiency	20	4,507	27	19,272	3.57E-08
ko05320	Autoimmune thyroid disease	16	4,507	19	19,272	3.62E-08
ko00531	Glycosaminoglycan degradation	29	4,507	49	19,272	8.23E-08
ko04610	Complement and coagulation cascades	55	4,507	123	19,272	1.46E-07
ko04622	RIG-I-like receptor signaling pathway	42	4,507	85	19,272	1.47E-07
ko00360	Phenylalanine metabolism	21	4,507	31	19,272	1.95E-07
ko00450	Selenoamino acid metabolism	35	4,507	67	19,272	2.82E-07
ko00260	Glycine, serine and threonine metabolism	35	4,507	67	19,272	2.82E-07
ko00920	Sulfur metabolism	13	4,507	15	19,272	3.98E-07
ko00790	Folate biosynthesis	15	4,507	19	19,272	4.87E-07
ko04672	Intestinal immune network for IgA production	15	4,507	19	19,272	4.87E-07
ko00053	Ascorbate and aldarate metabolism	22	4,507	35	19,272	7.10E-07
ko00052	Galactose metabolism	38	4,507	78	19,272	8.94E-07
ko00740	Riboflavin metabolism	22	4,507	36	19,272	1.42E-06
ko00511	Other glycan degradation	15	4,507	20	19,272	1.52E-06
ko05310	Asthma	9	4,507	9	19,272	2.08E-06
ko00710	Carbon fixation in photosynthetic organisms	25	4,507	45	19,272	3.25E-06
ko00120	Primary bile acid biosynthesis	16	4,507	23	19,272	3.41E-06
ko03018	RNA degradation	15	4,507	21	19,272	4.17E-06
ko00750	Vitamin B6 metabolism	10	4,507	11	19,272	4.21E-06
ko00460	Cyanoamino acid metabolism	18	4,507	28	19,272	4.66E-06
ko00903	Limonene and pinene degradation	22	4,507	38	19,272	5.05E-06

ko00340	Histidine metabolism	28	4,507	54	19,272	5.21E-06
ko04621	NOD-like receptor signaling pathway	34	4,507	71	19,272	5.38E-06
ko00642	Ethylbenzene degradation	11	4,507	13	19,272	5.47E-06
ko05330	Allograft rejection	11	4,507	13	19,272	5.47E-06
ko02020	Two-component system	11	4,507	13	19,272	5.47E-06
ko00521	Streptomycin biosynthesis	13	4,507	17	19,272	5.55E-06
ko04115	p53 signaling pathway	71	4,507	188	19,272	6.38E-06
ko00410	beta-Alanine metabolism	23	4,507	41	19,272	6.43E-06
ko00281	Geraniol degradation	8	4,507	8	19,272	8.90E-06
ko00601	Glycosphingolipid biosynthesis - lacto and neolacto se	30	4,507	61	19,272	9.71E-06
ko00760	Nicotinate and nicotinamide metabolism	27	4,507	53	19,272	1.17E-05
ko00563	Glycosylphosphatidylinositol	19	4,507	32	19,272	1.36E-05
ko00400	Phenylalanine, tyrosine and tryptophan biosynthesis	9	4,507	10	19,272	1.64E-05
ko00603	Glycosphingolipid biosynthesis - globo series	12	4,507	16	19,272	1.83E-05
ko05130	Pathogenic Escherichia coli infection	61	4,507	160	19,272	1.98E-05
ko00960	Tropane, piperidine and pyridine alkaloid biosynthesis	14	4,507	21	19,272	3.03E-05
ko04614	Renin-angiotensin system	15	4,507	24	19,272	4.82E-05
ko00130	Ubiquinone and other terpenoid-quinone biosynthesis	8	4,507	9	19,272	6.35E-05
ko05210	Colorectal cancer	76	4,507	217	19,272	6.52E-05
ko00430	Taurine and hypotaurine metabolism	14	4,507	22	19,272	6.53E-05
ko03440	Homologous recombination	23	4,507	46	19,272	7.52E-05
ko04110	Cell cycle	115	4,507	358	19,272	8.76E-05
ko00280	Valine, leucine and isoleucine degradation	37	4,507	89	19,272	1.04E-04
ko00680	Methane metabolism	12	4,507	18	19,272	1.15E-04
ko04623	Cytosolic DNA-sensing pathway	20	4,507	39	19,272	1.40E-04
ko04210	Apoptosis	76	4,507	222	19,272	1.51E-04
ko03020	RNA polymerase	29	4,507	66	19,272	1.80E-04
ko03410	Base excision repair	26	4,507	57	19,272	1.84E-04
ko00950	Isoquinoline alkaloid biosynthesis	10	4,507	14	19,272	1.88E-04
ko03040	Spliceosome	19	4,507	37	19,272	2.01E-04
ko00930	Caprolactam degradation	8	4,507	10	19,272	2.52E-04
ko05332	Graft-versus-host disease	8	4,507	10	19,272	2.52E-04

ko00604	Glycosphingolipid biosynthesis - ganglio series	18	4,507	35	19,272	2.87E-04
ko00600	Sphingolipid metabolism	41	4,507	106	19,272	2.99E-04
ko05142	Chagas disease	90	4,507	277	19,272	3.17E-04
ko05212	Pancreatic cancer	74	4,507	220	19,272	3.34E-04
ko00626	Naphthalene and anthracene degradation	12	4,507	20	19,272	4.85E-04
ko04080	Neuroactive ligand-receptor interaction	312	4,507	1,138	19,272	6.30E-04
ko04060	Cytokine-cytokine receptor interaction	87	4,507	272	19,272	6.90E-04
ko00232	Caffeine metabolism	5	4,507	5	19,272	6.98E-04
ko04710	Circadian rhythm - mammal	12	4,507	21	19,272	8.91E-04
ko00330	Arginine and proline metabolism	52	4,507	149	19,272	9.43E-04
ko04670	Leucyte transendothelial migration	157	4,507	538	19,272	9.67E-04
