# Supplementary material for: The integrated analysis of RNA-seq and microRNA-seq depicts miRNA-mRNA networks involved in Japanese flounder (Paralichthys olivaceus) albinism
Source: PLoS One. 2017 Aug 4;12(8):e0181761. doi: 10.1371/journal.pone.0181761 (PMC5544202; doi:10.1371/journal.pone.0181761)
Supplement: S16 Table — (PDF) [file pone.0181761.s020.pdf]

S16 Table. The overlapped genes between differentially expressed genes in RNA-seq and miRNAs' predicted target genes in miRNA-seq.

Gene ID	Gene Symbol	Gene Annotation
GS_000169	RGS8	sp|Q6DGI0|RGS8_DANRE Regulator of G-protein signaling 8 OS=Danio rerio GN=rgs8
GS_000465	CO1A2	sp|Q28668|CO1A2_RABIT Collagen alpha-2(I) chain (Fragment) OS=Oryctolagus cuniculus
GS_000622	MUCM	sp|P23735|MUCM_ICTPU Ig mu chain C region membrane-bound form OS=Ictalurus punctatus
GS_000805	-	-
GS_000806	-	-
GS_000869	MAR1	sp|Q16655|MAR1_HUMAN Melanoma antigen recognized by T-cells 1 OS=Homo sapiens GN=MLANA
GS_001154	KIF5C	sp|O60282|KIF5C_HUMAN Kinesin heavy chain isoform 5C OS=Homo sapiens GN=KIF5C
GS_001157	ABP1	sp|P36633|ABP1_RAT Amiloride-sensitive amine oxidase [copper-containing] OS=Rattus norvegicus GN=Abp1
GS_001591	NKX11	sp|Q15270|NKX11_HUMAN NK1 transcription factor-related protein 1 OS=Homo sapiens GN=NKX1-1
GS_001664	CYTSA	sp|Q2KN97|CYTSA_CHICK Cytospin-A OS=Gallus gallus GN=SPECC1L
GS_001783	KNG	sp|P83857|KNG_ANAMI Kininogen (Fragments) OS=Anarhichas minor
GS_002043	MITF	sp|O75030|MITF_HUMAN Microphthalmia-associated transcription factor OS=Homo sapiens GN=MITF
GS_002131	MSTN1	sp|Q05AX4|MSTN1_XENLA Musculoskeletal embryonic nuclear protein 1 OS=Xenopus laevis GN=mustn1
GS_002188	AQP7	sp|O54794|AQP7_MOUSE Aquaporin-7 OS=Mus musculus GN=Aqp7
GS_002614	GPR21	sp|Q99679|GPR21_HUMAN Probable G-protein coupled receptor 21 OS=Homo sapiens GN=GPR21
GS_002681	DRD5L	sp|P53454|DRD5L_TAKRU D(5)-like dopamine receptor OS=Takifugu rubripes GN=dl
GS_002818	POL	sp|O92815|POL_WDSV Gag-Pol polyprotein OS=Walleye dermal sarcoma virus GN=gag-pol
GS_002994	CRP	sp|Q07203|CRP_XENLA C-reactive protein OS=Xenopus laevis GN=crp
GS_003073	HSP70	sp|P08108|HSP70_ONCMY Heat shock cognate 70 kDa protein OS=Oncorhynchus mykiss GN=hsc71
GS_003590	PMEL	sp|Q98917|PMEL_CHICK Melanocyte protein PMEL OS=Gallus gallus GN=PMEL
GS_003635	PDIA1	sp|P09102|PDIA1_CHICK Protein disulfide-isomerase OS=Gallus gallus GN=P4HB
GS_003739	BDH	sp|P29147|BDH_RAT D-beta-hydroxybutyrate dehydrogenase, mitochondrial OS=Rattus norvegicus GN=Bdh1
GS_003949	S47A1	sp|A4IIS8|S47A1_XENTR Multidrug and toxin extrusion protein 1 OS=Xenopus tropicalis GN=slc47a1
GS_004023	ARF6	sp|Q9Y7Z2|ARF6_SCHPO ADP-ribosylation factor 6 OS=Schizosaccharomyces pombe (strain 972 / ATCC 24843) GN=arf6
GS_004597	MREG	sp|Q6GQM0|MREG_DANRE Melanoregulin OS=Danio rerio GN=mreg
GS_005047	F123A	sp|Q9TUM6|PLIN2_BOVIN Perilipin-2 OS=Bos taurus GN=PLIN2
GS_005244	-	-
GS_005575	NCF1	sp|O77774|NCF1_BOVIN Neutrophil cytosol factor 1 OS=Bos taurus GN=NCF1
GS_005670	EZRI	sp|P15311|EZRI_HUMAN Ezrin OS=Homo sapiens GN=EZR
GS_005871	REP15	sp|Q6BDI9|REP15_HUMAN Rab15 effector protein OS=Homo sapiens GN=REP15
GS_005902	GCH1	sp|O61573|GCH1_OSTOS GTP cyclohydrolase 1 OS=Ostertagia ostertagi GN=gch
GS_005903	GCH1	sp|O61573|GCH1_OSTOS GTP cyclohydrolase 1 OS=Ostertagia ostertagi GN=gch
GS_005905	GILT	sp|Q499T2|GILT_RAT Gamma-interferon-inducible lysosomal thiol reductase OS=Rattus norvegicus GN=Ifi30

GS_006243	S6A13	sp|A5PJX7|S6A13_BOVIN Sodium- and chloride-dependent GABA transporter 2 OS=Bos taurus GN=SLC6A13
GS_006323	DLRB2	sp|Q32P85|DLRB2_BOVIN Dynein light chain roadblock-type 2 OS=Bos taurus GN=DYNLRB2
GS_006488	P2RY8	sp|Q5ZI82|P2RY8_CHICK P2Y purinoceptor 8 OS=Gallus gallus GN=P2RY8
GS_006925	PGS1	sp|P21810|PGS1_HUMAN Biglycan OS=Homo sapiens GN=BGN
GS_006975	GLRK	sp|P19439|GLRK_CHICK Probable glutamate receptor OS=Gallus gallus GN=KBP
GS_007052	DYR	sp|P09503|DYR_SHV21 Viral dihydrofolate reductase OS=Saimiriine herpesvirus 2 (strain 11) GN=DHFR
GS_007314	GRN	sp|P28799|GRN_HUMAN Granulins OS=Homo sapiens GN=GRN
GS_007375	S22A7	sp|Q3YAW7|S22A7_RABIT Solute carrier family 22 member 7 OS=Oryctolagus cuniculus GN=SLC22A7
GS_007378	MC1R	sp|P55167|MSHR_CHICK Melanocyte-stimulating hormone receptor OS=Gallus gallus GN=MC1R
GS_007378	MSHR	sp|P55167|MSHR_CHICK Melanocyte-stimulating hormone receptor OS=Gallus gallus GN=MC1R
GS_007481	KCC1G	sp|Q7TNJ7|KCC1G_RAT Calcium/calmodulin-dependent protein kinase type 1G OS=Rattus norvegicus GN=Camk1g
GS_007697	SOSD1	sp|Q9CQN4|SOSD1_MOUSE Sclerostin domain-containing protein 1 OS=Mus musculus GN=Sostdc1
GS_007760	CP26C	sp|Q6V0L0|CP26C_HUMAN Cytochrome P450 26C1 OS=Homo sapiens GN=CYP26C1
GS_008156	CHLE	sp|Q03311|CHLE_MOUSE Cholinesterase OS=Mus musculus GN=Bche
GS_008205	FXI1C	sp|Q8JIT5|FXI1C_XENLA Forkhead box protein I1c OS=Xenopus laevis GN=foxi1c
GS_008220	AHNK	sp|Q09666|AHNK_HUMAN Neuroblast differentiation-associated protein AHNAK OS=Homo sapiens GN=AHNAK
GS_008287	PRS35	sp|Q5E9X7|PRS35_BOVIN Inactive serine protease 35 OS=Bos taurus GN=PRSS35
GS_008590	FICA	sp|Q9YGN5|FICA_AGKHB Salmorin subunit A OS=Agkistrodon halys brevicaudus
GS_008611	ADRB1	sp|O42574|ADRB1_XENLA Beta-1 adrenergic receptor OS=Xenopus laevis GN=adrb1
GS_008748	GCH1	sp|P48596|GCH1_DROME GTP cyclohydrolase 1 OS=Drosophila melanogaster GN=Pu
GS_008785	CAH4	sp|P48283|CAH4_RABIT Carbonic anhydrase 4 OS=Oryctolagus cuniculus GN=CA4
GS_009046	KITA	sp|Q8JFR5|KITA_DANRE Mast/stem cell growth factor receptor kita OS=Danio rerio GN=kita
GS_009380	FRPA	sp|Q9K0K9|FRPA_NEIMB Iron-regulated protein frpA OS=Neisseria meningitidis serogroup B GN=frpA
GS_009467	TNNT2	sp|P02642|TNNT2_CHICK Troponin T, cardiac muscle isoforms OS=Gallus gallus GN=TNNT2
GS_009601	PLD3A	sp|A5D6R3|PLD3A_DANRE 1-phosphatidylinositol 4,5-bisphosphate phosphodiesterase delta-3-A OS=Danio rerio GN=plcd3a
GS_009938	DHB14	sp|Q9BPX1|DHB14_HUMAN 17-beta-hydroxysteroid dehydrogenase 14 OS=Homo sapiens GN=HSD17B14
GS_010021	PHOP1	sp|Q6DBV4|PHOP1_DANRE Probable phosphatase phospho1 OS=Danio rerio GN=phospho1
GS_010174	LYG	sp|Q90VZ3|LYG_PAROL Lysozyme g OS=Paralichthys olivaceus
GS_010268	VEGFAA	sp|O73682|VGFAA_DANRE Vascular endothelial growth factor A-A OS=Danio rerio GN=vegfaa
GS_010295	MMP9	sp|P41245|MMP9_MOUSE Matrix metalloproteinase-9 OS=Mus musculus GN=Mmp9
GS_010316	GPR61	sp|Q9BZJ8|GPR61_HUMAN Probable G-protein coupled receptor 61 OS=Homo sapiens GN=GPR61
GS_010330	CAH6	sp|Q865C0|CAH6_CANFA Carbonic anhydrase 6 OS=Canis familiaris GN=CA6
GS_010503	-	-
GS_010525	FABPI	sp|Q56JX9|FABPI_BOVIN Fatty acid-binding protein, intestinal OS=Bos taurus GN=FABP2
GS_011013	HEBP2	sp|Q9Y5Z4|HEBP2_HUMAN Heme-binding protein 2 OS=Homo sapiens GN=HEBP2
GS_011014	-	-
GS_011090	RAP1GAP	sp|P47736|RPGP1_HUMAN Rap1 GTPase-activating protein 1 OS=Homo sapiens GN=RAP1GAP

GS_011506	ARG33	sp|Q8BW86|ARG33_MOUSE Rho guanine nucleotide exchange factor 33 OS=Mus musculus GN=Arhgef33
GS_011788	-	-
GS_011843	GNS	sp|P50426|GNS_CAPHI N-acetylglucosamine-6-sulfatase OS=Capra hircus GN=GNS
GS_012001	PLIN2	sp|Q99541|PLIN2_HUMAN Perilipin-2 OS=Homo sapiens GN=PLIN2
GS_012029	TYRP1	sp|P55028|TYRP1_CARAU 5,6-dihydroxyindole-2-carboxylic acid oxidase OS=Carassius auratus GN=tyrp1
GS_012394	K1B27	sp|Q9JM71|K1B27_MOUSE Kallikrein 1-related peptidase b27 OS=Mus musculus GN=Klk1b27
GS_012501	BMP1	sp|P98063|BMP1_MOUSE Bone morphogenetic protein 1 OS=Mus musculus GN=Bmp1
GS_012545	K1C17	sp|A5A6M0|K1C17_PANTR Keratin, type I cytoskeletal 17 OS=Pan troglodytes GN=KRT17
GS_012647	EDNRB	sp|Q9N0W7|EDNRB_RABIT Endothelin B receptor OS=Oryctolagus cuniculus GN=EDNRB
GS_012661	SOX10	sp|Q9W757|SOX10_CHICK Transcription factor SOX-10 OS=Gallus gallus GN=SOX10
GS_013015	PE2R3	sp|P46069|PE2R3_RABIT Prostaglandin E2 receptor EP3 subtype OS=Oryctolagus cuniculus GN=PTGER3
GS_013228	GCH1	sp|O61573|GCH1_OSTOS GTP cyclohydrolase 1 OS=Ostertagia ostertagi GN=gch
GS_013252	SATT	sp|O35874|SATT_MOUSE Neutral amino acid transporter A OS=Mus musculus GN=Slc1a4
GS_013312	DFP	sp|Q86RS3|DFP_MANSE Putative defense protein Hdd11-like OS=Manduca sexta
GS_013459	SIX3	sp|O95343|SIX3_HUMAN Homeobox protein SIX3 OS=Homo sapiens GN=SIX3
GS_013586	RASEF	sp|Q08CX1|RASEF_XENTR Ras and EF-hand domain-containing protein OS=Xenopus tropicalis GN=rasef
GS_013618	PRDM1	sp|Q60636|PRDM1_MOUSE PR domain zinc finger protein 1 OS=Mus musculus GN=Prdm1
GS_013701	RASFA	sp|B1A193|RASFA_XENLA Ras association domain-containing protein 10 OS=Xenopus laevis GN=rassf10
GS_014186	MSLNL	sp|Q96KJ4|MSLNL_HUMAN Mesothelin-like protein OS=Homo sapiens GN=MSLNL
GS_014384	RETST	sp|Q5BLE8|RETST_DANRE Putative all-trans-retinol 13,14-reductase OS=Danio rerio GN=retsat
GS_014478	TBX19	sp|Q5XNS0|TBX19_CANFA T-box transcription factor TBX19 OS=Canis familiaris GN=TBX19
GS_014490	XDH	sp|P47989|XDH_HUMAN Xanthine dehydrogenase/oxidase OS=Homo sapiens GN=XDH
GS_014502	CDK15	sp|Q1RLU9|CDK15_DANRE Cyclin-dependent kinase 15 OS=Danio rerio GN=cdk15
GS_014594	FCAMR	sp|Q2TB54|FCAMR_MOUSE High affinity immunoglobulin alpha and immunoglobulin mu Fc receptor OS=Mus musculus GN=Fcamr
GS_014684	GTR5	sp|Q5RET7|GTR5_PONAB Solute carrier family 2, facilitated glucose transporter member 5 OS=Pongo abelii GN=SLC2A5
GS_014685	GTR5	sp|Q9WV38|GTR5_MOUSE Solute carrier family 2, facilitated glucose transporter member 5 OS=Mus musculus GN=Slc2a5
GS_014776	PCD10	sp|Q9P2E7|PCD10_HUMAN Protocadherin-10 OS=Homo sapiens GN=PCDH10
GS_015091	PCDH10	sp|Q3U6Q4|PCD10_HUMAN Protocadherin-10 OS=Homo sapiens GN=PCDH10
GS_015155	PCDH9	sp|Q9HC56|PCDH9_HUMAN Protocadherin-9 OS=Homo sapiens GN=PCDH9
GS_015243	VTCN1	sp|Q501W4|VTCN1_RAT V-set domain-containing T-cell activation inhibitor 1 OS=Rattus norvegicus GN=Vtcn1
GS_015700	DMBT1	sp|Q8CIZ5|DMBT1_RAT Deleted in malignant brain tumors 1 protein OS=Rattus norvegicus GN=Dmbt1
GS_015726	RFT2	sp|B5X4H8|RFT2_SALSA Riboflavin transporter 2 OS=Salmo salar GN=rft2
GS_015779	CA216	sp|Q52KN3|CA216_XENLA UPF0500 protein C1orf216 homolog OS=Xenopus laevis
GS_016285	S10I	sp|Q91061|S10I_ICTPU Ictacalcin OS=Ictalurus punctatus
GS_016413	C1ORF106	sp|Q3KP66|CA106_HUMAN Uncharacterized protein C1orf106 OS=Homo sapiens GN=C1orf106
GS_016685	MSMB	sp|O02826|MSMB_PIG Beta-microseminoprotein OS=Sus scrofa GN=MSMB
GS_016790	AHI1	sp|Q8N157|AHI1_HUMAN Jouberin OS=Homo sapiens GN=AHI1

GS_016931	TAC2N	sp|Q8N9U0|TAC2N_HUMAN Tandem C2 domains nuclear protein OS=Homo sapiens GN=TC2N
GS_017127	CCD18	sp|Q5T9S5|CCD18_HUMAN Coiled-coil domain-containing protein 18 OS=Homo sapiens GN=CCDC18
GS_017738	NPDC1	sp|Q9NQX5|NPDC1_HUMAN Neural proliferation differentiation and control protein 1 OS=Homo sapiens GN=NPDC1
GS_017984	LIX1	sp|Q8UVV7|LIX1_CHICK Protein limb expression 1 OS=Gallus gallus GN=LIX1
GS_017985	CCD42	sp|A6QQM8|CCD42_BOVIN Coiled-coil domain-containing protein 42A OS=Bos taurus GN=CCDC42
GS_018185	HAVR1	sp|Q5QNS5|HAVR1_MOUSE Hepatitis A virus cellular receptor 1 homolog OS=Mus musculus GN=Havcr1
GS_018226	ES1	sp|P30042|ES1_HUMAN ES1 protein homolog, mitochondrial OS=Homo sapiens GN=C21orf33
GS_018359	EDNRB	sp|Q9N0W7|EDNRB_RABIT Endothelin B receptor OS=Oryctolagus cuniculus GN=EDNRB
GS_018739	EPD	sp|P38528|EPD_CYPCA Ependymin OS=Cyprinus carpio GN=epd
GS_018934	RGR	sp|P47804|RGR_HUMAN RPE-retinal G protein-coupled receptor OS=Homo sapiens GN=RGR
GS_019376	HS3S6	sp|Q5GFD5|HS3S6_MOUSE Heparan sulfate glucosamine 3-O-sulfotransferase 6 OS=Mus musculus GN=Hs3st6
GS_019504	TRPC2	sp|Q9R283|TRPC2_RAT Short transient receptor potential channel 2 OS=Rattus norvegicus GN=Trpc2
GS_019550	CXA3	sp|Q64448|CXA3_MOUSE Gap junction alpha-3 protein OS=Mus musculus GN=Gja3
GS_019678	MPDZ	sp|Q8VBX6|MPDZ_MOUSE Multiple PDZ domain protein OS=Mus musculus GN=Mpdz
GS_019698	MAP6	sp|O73737|MAP6_CHICK Microtubule-associated protein 6 homolog OS=Gallus gallus GN=MAP6
GS_020150	KINH	sp|Q2PQA9|KINH_RAT Kinesin-1 heavy chain OS=Rattus norvegicus GN=Kif5b
GS_020318	DESM	sp|P23239|DESM_XENLA Desmin OS=Xenopus laevis GN=des
GS_020430	DES	sp|Q5XFN2|DESM_CANFA Desmin OS=Canis familiaris GN=DES
GS_020470	TT39B	sp|Q28DB0|TT39B_XENTR Tetratricopeptide repeat protein 39B OS=Xenopus tropicalis GN=ttc39b
GS_020687	TMEM130	sp|Q8N3G9|TM130_HUMAN Transmembrane protein 130 OS=Homo sapiens GN=TMEM130
GS_020906	BACE1	sp|Q1KLR6|BACE1_CAVPO Beta-secretase 1 OS=Cavia porcellus GN=BACE1
GS_020975	TYR	sp|P55025|TYRO_ORYLA Tyrosinase OS=Oryzias latipes GN=tyr
GS_021002	PNPH	sp|P55859|PNPH_BOVIN Purine nucleoside phosphorylase OS=Bos taurus GN=PNP
GS_021037	IL17REL	sp|Q6ZVW7|I17EL_HUMAN Putative interleukin-17 receptor E-like OS=Homo sapiens GN=IL17REL
GS_021046	WNT7B	sp|Q3L254|WNT7B_CHICK Protein Wnt-7b OS=Gallus gallus GN=WNT7B
GS_021169	NSG2	sp|Q9Y328|NSG2_HUMAN Neuron-specific protein family member 2 OS=Homo sapiens GN=NSG2
GS_021359	PANX3	sp|Q8CEG0|PANX3_MOUSE Pannexin-3 OS=Mus musculus GN=Panx3
