# Supplementary material for: The integrated analysis of RNA-seq and microRNA-seq depicts miRNA-mRNA networks involved in Japanese flounder (Paralichthys olivaceus) albinism
Source: PLoS One. 2017 Aug 4;12(8):e0181761. doi: 10.1371/journal.pone.0181761 (PMC5544202; doi:10.1371/journal.pone.0181761)
Supplement: S17 Table — (PDF) [file pone.0181761.s021.pdf]

S17 Table. The comparison of expression profiles between miRNAs and the predicted mRNA targets in PO_alb versus PO_con.

mRNA ID	mRNA symbol	mRNA annotation	mRNA profiles	miRNAs	miRNA profiles
GS_000169	RGS8	Regulator of G-protein signaling 8	down	dre-miR-25-3p	up
GS_000169	RGS8	Regulator of G-protein signaling 8	down	xtr-miR-92a_R+4	up
GS_000622	MUCM	Ig mu chain C region membrane-bound form	down	mmu-miR-143-5p_R+2	up
GS_000622	MUCM	Ig mu chain C region membrane-bound form	down	xtr-miR-222_R-1	up
GS_000805	-	-	down	mmu-miR-143-5p_R+2	up
GS_000805	-	-	down	ola-miR-30c_1ss21AG	up
GS_000869	MAR1	Melanoma antigen recognized by T-cells 1	down	dre-miR-25-3p	up
GS_000869	MAR1	Melanoma antigen recognized by T-cells 1	down	xtr-miR-92a_R+4	up
GS_001154	KIF5C	Kinesin heavy chain isoform 5C	down	hsa-miR-27a-3p_R+1	up
GS_001664	CYTSA	Cytospin-A	down	hsa-miR-205-5p_R+2	up
GS_001664	CYTSA	Cytospin-A	down	mmu-miR-143-5p_R+2	up
GS_001664	CYTSA	Cytospin-A	down	tni-miR-205	up
GS_002043	MITF	Microphthalmia-associated transcription factor	down	hsa-miR-27a-3p_R+1	up
GS_002681	DRD5L	D(5)-like dopamine receptor	down	hsa-miR-27a-3p_R+1	up
GS_002818	POL	Gag-Pol polyprotein	down	hsa-miR-27a-3p_R+1	up
GS_003590	PMEL	Melanocyte protein PMEL	down	hsa-miR-27a-3p_R+1	up
GS_004597	MREG	Melanoregulin	down	ola-miR-30c_1ss21AG	up
GS_004597	MREG	Melanoregulin	down	xtr-miR-222_R-1	up
GS_005047	F123A	Perilipin-2	down	pol-miR-199a-5p_R+1	up
GS_005575	NCF1	Neutrophil cytosol factor 1	down	hsa-miR-27a-3p_R+1	up
GS_005871	REP15	Rab15 effector protein	down	hsa-miR-205-5p_R+2	up
GS_005871	REP15	Rab15 effector protein	down	mmu-miR-143-5p_R+2	up
GS_005871	REP15	Rab15 effector protein	down	tni-miR-205	up
GS_005902	GCH1	GTP cyclohydrolase 1	down	mmu-miR-143-5p_R+2	up
GS_005902	GCH1	GTP cyclohydrolase 1	down	xtr-miR-222_R-1	up
GS_006243	S6A13	Sodium- and chloride-dependent GABA transporter 2	down	ola-miR-30c_1ss21AG	up
GS_007375	S22A7	Solute carrier family 22 member 7	down	oan-miR-139-3p_R-1_1ss8AC	up
GS_007378	MC1R	Melanocyte-stimulating hormone receptor	down	dre-miR-25-3p	up
GS_007378	MC1R	Melanocyte-stimulating hormone receptor	down	hsa-miR-205-5p_R+2	up
GS_007378	MC1R	Melanocyte-stimulating hormone receptor	down	hsa-miR-27a-3p_R+1	up
GS_007378	MC1R	Melanocyte-stimulating hormone receptor	down	tni-miR-205	up
GS_007378	MC1R	Melanocyte-stimulating hormone receptor	down	xtr-miR-92a_R+4	up

GS_008156	CHLE	Melanocyte-stimulating hormone receptor	down	pol-miR-199a-5p_R+1	up
GS_008205	FXI1C	Forkhead box protein I1c	down	hsa-miR-27a-3p_R+1	up
GS_008205	FXI1C	Forkhead box protein I1c	down	oan-miR-139-3p_R-1_1ss8AC	up
GS_008220	AHNK	Neuroblast differentiation-associated protein AHNAK	down	ola-miR-30c_1ss21AG	up
GS_008748	GCH1	GTP cyclohydrolase 1	down	hsa-miR-27a-3p_R+1	up
GS_009601	PLD3A	1-phosphatidylinositol 4,5-bisphosphate phosphodiesterase delta-3-A	down	ola-miR-30c_1ss21AG	up
GS_010316	GPR61	Probable G-protein coupled receptor 61	down	hsa-miR-205-5p_R+2	up
GS_010316	GPR61	Probable G-protein coupled receptor 61	down	mmu-miR-143-5p_R+2	up
GS_010316	GPR61	Probable G-protein coupled receptor 61	down	tni-miR-205	up
GS_010330	CAH6	Carbonic anhydrase 6	down	hsa-miR-205-5p_R+2	up
GS_010330	CAH6	Carbonic anhydrase 6	down	tni-miR-205	up
GS_010503	-	-	down	pol-miR-199a-5p_R+1	up
GS_011013	HEBP2	Heme-binding protein 2	down	xtr-miR-222_R-1	up
GS_012545	K1C17	Keratin, type I cytoskeletal 17	down	mmu-miR-143-5p_R+2	up
GS_013312	DFP	Putative defense protein Hdd11-like	down	mmu-miR-143-5p_R+2	up
GS_013586	RASEF	Ras and EF-hand domain-containing protein	down	dre-miR-25-3p	up
GS_013586	RASEF	Ras and EF-hand domain-containing protein	down	xtr-miR-92a_R+4	up
GS_014384	RETST	Putative all-trans-retinol 13,14-reductase	down	xtr-miR-222_R-1	up
GS_014490	XDH	Xanthine dehydrogenase/oxidase	down	oan-miR-139-3p_R-1_1ss8AC	up
GS_014594	FCAMR	High affinity immunoglobulin alpha and immunoglobulin mu Fc receptor	down	mmu-miR-143-5p_R+2	up
GS_014776	PCD10	Protocadherin-10	down	ola-miR-30c_1ss21AG	up
GS_015155	PCDH9	Protocadherin-9	down	xtr-miR-92a_R+4	up
GS_015243	VTCN1	V-set domain-containing T-cell activation inhibitor 1	down	mmu-miR-143-5p_R+2	up
GS_015779	CA216	UPF0500 protein C1orf216 homolog	down	ola-miR-30c_1ss21AG	up
GS_016285	S10I	Ictacalcin	down	hsa-miR-27a-3p_R+1	up
GS_016285	S10I	Ictacalcin	down	ola-miR-30c_1ss21AG	up
GS_016285	S10I	Ictacalcin	down	pol-miR-199a-5p_R+1	up
GS_016685	MSMB	Beta-microseminoprotein	down	ola-miR-30c_1ss21AG	up
GS_016790	AHI1	Jouberin	down	hsa-miR-27a-3p_R+1	up
GS_017127	CCD18	Coiled-coil domain-containing protein 18	down	hsa-miR-205-5p_R+2	up
GS_017127	CCD18	Coiled-coil domain-containing protein 18	down	tni-miR-205	up
GS_017738	NPDC1	Neural proliferation differentiation and control protein 1	down	ola-miR-30c_1ss21AG	up
GS_018226	ES1	ES1 protein homolog, mitochondrial	down	pol-miR-199a-5p_R+1	up
GS_018359	EDNRB	Endothelin B receptor	down	xtr-miR-222_R-1	up
GS_018934	RGR	RPE-retinal G protein-coupled receptor	down	hsa-miR-27a-3p_R+1	up
GS_019504	TRPC2	Short transient receptor potential channel 2	down	ola-miR-30c_1ss21AG	up

GS_019678	MPDZ	Multiple PDZ domain protein	down	dre-miR-25-3p	up
GS_019678	MPDZ	Multiple PDZ domain protein	down	xtr-miR-92a_R+4	up
GS_019698	MAP6	Microtubule-associated protein 6 homolog	down	pol-miR-199a-5p_R+1	up
GS_020150	KINH	Kinesin-1 heavy chain	down	hsa-miR-27a-3p_R+1	up
GS_020687	TMEM130	Transmembrane protein 130	down	xtr-miR-222_R-1	up
GS_020906	BACE1	Beta-secretase 1	down	hsa-miR-27a-3p_R+1	up
GS_020906	BACE1	Beta-secretase 1	down	pol-miR-199a-5p_R+1	up
GS_021037	IL17REL	Putative interleukin-17 receptor E-like	down	oan-miR-139-3p_R-1_1ss8AC	up
GS_021169	NSG2	Neuron-specific protein family member 2	down	hsa-miR-205-5p_R+2	up
GS_021169	NSG2	Neuron-specific protein family member 2	down	tni-miR-205	up
GS_021359	PANX3	Pannexin-3	down	hsa-miR-205-5p_R+2	up
GS_021359	PANX3	Pannexin-3	down	tni-miR-205	up
GS_000465	CO1A2	Collagen alpha-2(I) chain (Fragment)	up	dre-miR-203b-3p_L-1R+2_1ss11CT	down
GS_000465	CO1A2	Collagen alpha-2(I) chain (Fragment)	up	dre-miR-204-5p_R+2	down
GS_001591	NKX11	NK1 transcription factor-related protein 1	up	dre-miR-18b-5p_1ss11TC	down
GS_001591	NKX11	NK1 transcription factor-related protein 1	up	pma-miR-1c-3p_1ss2GT	down
GS_002188	AQP7	Aquaporin-7	up	dre-miR-20a-3p_2ss7GA11GA	down
GS_002994	CRP	C-reactive protein	up	ola-miR-135b_R+3	down
GS_002994	CRP	C-reactive protein	up	PC-5p-43190_23	down
GS_002994	CRP	C-reactive protein	up	tni-miR-135b_R+1	down
GS_003073	HSP70	Heat shock cognate 70 kDa protein	up	dre-miR-203b-3p_L-1R+2_1ss11CT	down
GS_003073	HSP70	Heat shock cognate 70 kDa protein	up	ola-miR-106a_R+2	down
GS_004023	ARF6	ADP-ribosylation factor 6	up	pma-miR-1c-3p_1ss2GT	down
GS_006488	P2RY8	P2Y purinoceptor 8	up	dre-miR-203b-3p_L-1R+2_1ss11CT	down
GS_006488	P2RY8	P2Y purinoceptor 8	up	dre-miR-20a-3p_2ss7GA11GA	down
GS_006975	GLRK	Probable glutamate receptor	up	oan-miR-16b-5p_1ss22GA	down
GS_006975	GLRK	Probable glutamate receptor	up	ola-miR-16	down
GS_006975	GLRK	Probable glutamate receptor	up	PC-5p-26963_57	down
GS_006975	GLRK	Probable glutamate receptor	up	pma-miR-1c-3p_1ss2GT	down
GS_006975	GLRK	Probable glutamate receptor	up	tni-miR-15a_R-1	down
GS_006975	GLRK	Probable glutamate receptor	up	tni-miR-16_R-1	down
GS_007314	GRN	Granulins	up	dre-miR-20a-3p_2ss7GA11GA	down
GS_007314	GRN	Granulins	up	PC-5p-43190_23	down
GS_007481	KCC1G	Calcium/calmodulin-dependent protein kinase type 1G	up	ola-miR-106a_R+2	down
GS_007697	SOSD1	Sclerostin domain-containing protein 1	up	ola-miR-205_L-1R+1_1ss20TA	down
GS_008785	CAH4	Carbonic anhydrase 4	up	dre-miR-203b-3p_L-1R+2_1ss11CT	down
GS_009467	TNNT2	Troponin T, cardiac muscle isoforms	up	ola-miR-205_L-1R+1_1ss20TA	down

GS_009938	DHB14	17-beta-hydroxysteroid dehydrogenase 14	up	dre-miR-203b-3p_L-1R+2_1ss11CT	down
GS_009938	DHB14	17-beta-hydroxysteroid dehydrogenase 14	up	dre-miR-20a-3p_2ss7GA11GA	down
GS_010174	LYG	Lysozyme g	up	dre-miR-203b-3p_L-1R+2_1ss11CT	down
GS_010268	VEGFAA	Vascular endothelial growth factor A-A	up	oan-miR-16b-5p_1ss22GA	down
GS_010268	VEGFAA	Vascular endothelial growth factor A-A	up	ola-miR-16	down
GS_010268	VEGFAA	Vascular endothelial growth factor A-A	up	PC-5p-26963_57	down
GS_010268	VEGFAA	Vascular endothelial growth factor A-A	up	PC-5p-43190_23	down
GS_010268	VEGFAA	Vascular endothelial growth factor A-A	up	tni-miR-15a_R-1	down
GS_010268	VEGFAA	Vascular endothelial growth factor A-A	up	tni-miR-16_R-1	down
GS_010525	FABPI	Fatty acid-binding protein, intestinal	up	PC-5p-26963_57	down
GS_012001	PLIN2	Perilipin-2	up	dre-miR-9-3p_R+1	down
GS_013015	PE2R3	Prostaglandin E2 receptor EP3 subtype	up	pma-miR-1c-3p_1ss2GT	down
GS_013015	PE2R3	Prostaglandin E2 receptor EP3 subtype	up	tni-miR-135b_R+1	down
GS_014186	MSLNL	Mesothelin-like protein	up	dre-miR-202-5p_R-1	down
GS_014186	MSLNL	Mesothelin-like protein	up	dre-miR-203b-3p_L-1R+2_1ss11CT	down
GS_014186	MSLNL	Mesothelin-like protein	up	ola-miR-106a_R+2	down
GS_014186	MSLNL	Mesothelin-like protein	up	ola-miR-205_L-1R+1_1ss20TA	down
GS_014186	MSLNL	Mesothelin-like protein	up	PC-5p-26963_57	down
GS_014186	MSLNL	Mesothelin-like protein	up	pma-miR-1c-3p_1ss2GT	down
GS_015700	DMBT1	Deleted in malignant brain tumors 1 protein	up	dre-miR-202-5p_R-1	down
GS_019550	CXA3	Gap junction alpha-3 protein	up	oan-miR-16b-5p_1ss22GA	down
GS_019550	CXA3	Gap junction alpha-3 protein	up	ola-miR-16	down
GS_019550	CXA3	Gap junction alpha-3 protein	up	tni-miR-15a_R-1	down
GS_019550	CXA3	Gap junction alpha-3 protein	up	tni-miR-16_R-1	down
GS_020318	DESM	Desmin	up	ola-miR-106a_R+2	down
GS_000622	MUCM	Ig mu chain C region membrane-bound form	down	ola-miR-106a_R+2	down
GS_000805	-	-	down	ola-miR-205_L-1R+1_1ss20TA	down
GS_000806	-	-	down	dre-miR-203b-3p_L-1R+2_1ss11CT	down
GS_000806	-	-	down	ola-miR-205_L-1R+1_1ss20TA	down
GS_000869	MAR1	Melanoma antigen recognized by T-cells 1	down	oan-miR-16b-5p_1ss22GA	down
GS_000869	MAR1	Melanoma antigen recognized by T-cells 1	down	ola-miR-135b_R+3	down
GS_000869	MAR1	Melanoma antigen recognized by T-cells 1	down	ola-miR-16	down
GS_000869	MAR1	Melanoma antigen recognized by T-cells 1	down	tni-miR-135b_R+1	down
GS_000869	MAR1	Melanoma antigen recognized by T-cells 1	down	tni-miR-15a_R-1	down
GS_000869	MAR1	Melanoma antigen recognized by T-cells 1	down	tni-miR-16_R-1	down
GS_001154	KIF5C	Kinesin heavy chain isoform 5C	down	dre-miR-204-5p_R+2	down
GS_001157	ABP1	Amiloride-sensitive amine oxidase [copper-containing]	down	dre-miR-204-5p_L+1	down

GS_001664	CYTSA	Cytospin-A	down	dre-miR-203b-3p_L-1R+2_1ss11CT	down
GS_001664	CYTSA	Cytospin-A	down	ola-miR-106a_R+2	down
GS_001783	KNG	Kininogen (Fragments)	down	dre-miR-203b-3p_L-1R+2_1ss11CT	down
GS_002043	MITF	Microphthalmia-associated transcription factor	down	dre-miR-204-5p_R+2	down
GS_002043	MITF	Microphthalmia-associated transcription factor	down	PC-5p-46961_19	down
GS_002614	GPR21	Probable G-protein coupled receptor 21	down	oan-miR-16b-5p_1ss22GA	down
GS_002614	GPR21	Probable G-protein coupled receptor 21	down	ola-miR-16	down
GS_002614	GPR21	Probable G-protein coupled receptor 21	down	tni-miR-15a_R-1	down
GS_002614	GPR21	Probable G-protein coupled receptor 21	down	tni-miR-16_R-1	down
GS_002681	DRD5L	D(5)-like dopamine receptor	down	dre-miR-203b-3p_L-1R+2_1ss11CT	down
GS_002681	DRD5L	D(5)-like dopamine receptor	down	dre-miR-9-3p_R+1	down
GS_002818	POL	Gag-Pol polyprotein	down	oan-miR-16b-5p_1ss22GA	down
GS_002818	POL	Gag-Pol polyprotein	down	ola-miR-16	down
GS_002818	POL	Gag-Pol polyprotein	down	tni-miR-15a_R-1	down
GS_002818	POL	Gag-Pol polyprotein	down	tni-miR-16_R-1	down
GS_003590	PMEL	Melanocyte protein PMEL	down	oan-miR-16b-5p_1ss22GA	down
GS_003590	PMEL	Melanocyte protein PMEL	down	ola-miR-16	down
GS_003590	PMEL	Melanocyte protein PMEL	down	ola-miR-205_L-1R+1_1ss20TA	down
GS_003590	PMEL	Melanocyte protein PMEL	down	tni-miR-15a_R-1	down
GS_003590	PMEL	Melanocyte protein PMEL	down	tni-miR-16_R-1	down
GS_003635	PDIA1	Protein disulfide-isomerase	down	PC-5p-26963_57	down
GS_003739	BDH	D-beta-hydroxybutyrate dehydrogenase, mitochondrial	down	dre-miR-203b-3p_L-1R+2_1ss11CT	down
GS_003739	BDH	D-beta-hydroxybutyrate dehydrogenase, mitochondrial	down	PC-5p-43190_23	down
GS_003949	S47A1	Multidrug and toxin extrusion protein 1	down	dre-miR-202-5p_R-1	down
GS_004597	MREG	Melanoregulin	down	ola-miR-16	down
GS_004597	MREG	Melanoregulin	down	tni-miR-15a_R-1	down
GS_004597	MREG	Melanoregulin	down	tni-miR-16_R-1	down
GS_005047	F123A	Perilipin-2	down	ola-miR-205_L-1R+1_1ss20TA	down
GS_005244	-	-	down	ola-miR-106a_R+2	down
GS_005575	NCF1	Neutrophil cytosol factor 1	down	dre-miR-18b-5p_1ss11TC	down
GS_005670	EZRI	Ezrin	down	dre-miR-203b-3p_L-1R+2_1ss11CT	down
GS_005670	EZRI	Ezrin	down	dre-miR-9-3p_R+1	down
GS_005670	EZRI	Ezrin	down	ola-miR-106a_R+2	down
GS_005871	REP15	Rab15 effector protein	down	PC-5p-43190_23	down
GS_005903	GCH1	GTP cyclohydrolase 1	down	pma-miR-1c-3p_1ss2GT	down
GS_005905	GILT	Gamma-interferon-inducible lysosomal thiol reductase	down	oan-miR-16b-5p_1ss22GA	down
GS_005905	GILT	Gamma-interferon-inducible lysosomal thiol reductase	down	ola-miR-16	down

GS_005905	GILT	Gamma-interferon-inducible lysosomal thiol reductase	down	tni-miR-15a_R-1	down
GS_005905	GILT	Gamma-interferon-inducible lysosomal thiol reductase	down	tni-miR-16_R-1	down
GS_006243	S6A13	Sodium- and chloride-dependent GABA transporter 2	down	dre-miR-203b-3p_L-1R+2_1ss11CT	down
GS_006243	S6A13	Sodium- and chloride-dependent GABA transporter 2	down	oan-miR-16b-5p_1ss22GA	down
GS_006243	S6A13	Sodium- and chloride-dependent GABA transporter 2	down	ola-miR-16	down
GS_006243	S6A13	Sodium- and chloride-dependent GABA transporter 2	down	tni-miR-15a_R-1	down
GS_006243	S6A13	Sodium- and chloride-dependent GABA transporter 2	down	tni-miR-16_R-1	down
GS_006323	DLRB2	Dynein light chain roadblock-type 2	down	dre-miR-204-5p_R+2	down
GS_006323	DLRB2	Dynein light chain roadblock-type 2	down	oan-miR-16b-5p_1ss22GA	down
GS_006323	DLRB2	Dynein light chain roadblock-type 2	down	ola-miR-16	down
GS_006323	DLRB2	Dynein light chain roadblock-type 2	down	tni-miR-15a_R-1	down
GS_006323	DLRB2	Dynein light chain roadblock-type 2	down	tni-miR-16_R-1	down
GS_006925	PGS1	Biglycan	down	dre-miR-204-5p_R+2	down
GS_006925	PGS1	Biglycan	down	dre-miR-20a-3p_2ss7GA11GA	down
GS_007052	DYR	Viral dihydrofolate reductase	down	dre-miR-203b-3p_L-1R+2_1ss11CT	down
GS_007378	MC1R	Melanocyte-stimulating hormone receptor	down	oan-miR-16b-5p_1ss22GA	down
GS_007378	MC1R	Melanocyte-stimulating hormone receptor	down	ola-miR-16	down
GS_007378	MC1R	Melanocyte-stimulating hormone receptor	down	pma-miR-1c-3p_1ss2GT	down
GS_007378	MC1R	Melanocyte-stimulating hormone receptor	down	tni-miR-15a_R-1	down
GS_007378	MC1R	Melanocyte-stimulating hormone receptor	down	tni-miR-16_R-1	down
GS_007760	CP26C	Cytochrome P450 26C1	down	ola-miR-205_L-1R+1_1ss20TA	down
GS_008156	CHLE	Cholinesterase	down	dre-miR-20a-3p_2ss7GA11GA	down
GS_008156	CHLE	Cholinesterase	down	oan-miR-16b-5p_1ss22GA	down
GS_008156	CHLE	Cholinesterase	down	ola-miR-16	down
GS_008156	CHLE	Cholinesterase	down	pma-miR-1c-3p_1ss2GT	down
GS_008156	CHLE	Cholinesterase	down	tni-miR-15a_R-1	down
GS_008156	CHLE	Cholinesterase	down	tni-miR-16_R-1	down
GS_008220	AHNK	Neuroblast differentiation-associated protein AHNAK	down	dre-miR-203b-3p_L-1R+2_1ss11CT	down
GS_008287	PRS35	Inactive serine protease 35	down	dre-miR-9-3p_R+1	down
GS_008590	FICA	Salmorin subunit A	down	dre-miR-203b-3p_L-1R+2_1ss11CT	down
GS_008590	FICA	Salmorin subunit A	down	dre-miR-20a-3p_2ss7GA11GA	down
GS_008611	ADRB1	Beta-1 adrenergic receptor	down	dre-miR-204-5p_L+1	down
GS_008748	GCH1	GTP cyclohydrolase 1	down	dre-miR-202-5p_R-1	down
GS_009046	KITA	Mast/stem cell growth factor receptor kita	down	oan-miR-16b-5p_1ss22GA	down
GS_009046	KITA	Mast/stem cell growth factor receptor kita	down	ola-miR-16	down
GS_009046	KITA	Mast/stem cell growth factor receptor kita	down	ola-miR-205_L-1R+1_1ss20TA	down
GS_009046	KITA	Mast/stem cell growth factor receptor kita	down	tni-miR-15a_R-1	down

GS_009046	KITA	Mast/stem cell growth factor receptor kita	down	tni-miR-16_R-1	down
GS_009380	FRPA	Iron-regulated protein frpA	down	ola-miR-205_L-1R+1_1ss20TA	down
GS_009601	PLD3A	1-phosphatidylinositol 4,5-bisphosphate phosphodiesterase delta-3-A	down	dre-miR-203b-3p_L-1R+2_1ss11CT	down
GS_009601	PLD3A	1-phosphatidylinositol 4,5-bisphosphate phosphodiesterase delta-3-A	down	dre-miR-20a-3p_2ss7GA11GA	down
GS_009601	PLD3A	1-phosphatidylinositol 4,5-bisphosphate phosphodiesterase delta-3-A	down	oan-miR-16b-5p_1ss22GA	down
GS_009601	PLD3A	1-phosphatidylinositol 4,5-bisphosphate phosphodiesterase delta-3-A	down	ola-miR-16	down
GS_009601	PLD3A	1-phosphatidylinositol 4,5-bisphosphate phosphodiesterase delta-3-A	down	tni-miR-16_R-1	down
GS_010021	PHOP1	Probable phosphatase phospho1	down	dre-miR-20a-3p_2ss7GA11GA	down
GS_010295	MMP9	Matrix metalloproteinase-9	down	dre-miR-20a-3p_2ss7GA11GA	down
GS_010316	GPR61	Probable G-protein coupled receptor 61	down	dre-miR-203b-3p_L-1R+2_1ss11CT	down
GS_010503	-	-	down	pma-miR-1c-3p_1ss2GT	down
GS_011013	HEBP2	Heme-binding protein 2	down	PC-5p-43190_23	down
GS_011014	-	-	down	dre-miR-26a-5p_R-1_1ss21CT	down
GS_011014	-	-	down	hhi-miR-26_R+1	down
GS_011014	-	-	down	ola-miR-106a_R+2	down
GS_011014	-	-	down	ola-miR-205_L-1R+1_1ss20TA	down
GS_011090	RAP1GAP	Rap1 GTPase-activating protein 1	down	ola-miR-205_L-1R+1_1ss20TA	down
GS_011506	ARG33	Rho guanine nucleotide exchange factor 33	down	PC-5p-26963_57	down
GS_012029	TYRP1	5,6-dihydroxyindole-2-carboxylic acid oxidase	down	dre-miR-203b-3p_L-1R+2_1ss11CT	down
GS_012394	K1B27	Kallikrein 1-related peptidase b27	down	ola-miR-205_L-1R+1_1ss20TA	down
GS_012394	K1B27	Kallikrein 1-related peptidase b27	down	PC-5p-26963_57	down
GS_012501	BMP1	Bone morphogenetic protein 1	down	dre-miR-9-3p_R+1	down
GS_012501	BMP1	Bone morphogenetic protein 1	down	ola-miR-106a_R+2	down
GS_012647	EDNRB	Endothelin B receptor	down	dre-miR-9-3p_R+1	down
GS_012661	SOX10	Transcription factor SOX-10	down	dre-miR-26a-5p_R-1_1ss21CT	down
GS_012661	SOX10	Transcription factor SOX-10	down	hhi-miR-26_R+1	down
GS_013228	GCH1	GTP cyclohydrolase 1	down	dre-miR-204-5p_L+1	down
GS_013228	GCH1	GTP cyclohydrolase 1	down	dre-miR-204-5p_R+2	down
GS_013252	SATT	Neutral amino acid transporter A	down	dre-miR-203b-3p_L-1R+2_1ss11CT	down
GS_013252	SATT	Neutral amino acid transporter A	down	oan-miR-16b-5p_1ss22GA	down
GS_013252	SATT	Neutral amino acid transporter A	down	ola-miR-16	down
GS_013252	SATT	Neutral amino acid transporter A	down	tni-miR-15a_R-1	down
GS_013252	SATT	Neutral amino acid transporter A	down	tni-miR-16_R-1	down
GS_013312	DFP	Putative defense protein Hdd11-like	down	dre-miR-203b-3p_L-1R+2_1ss11CT	down
GS_013459	SIX3	Homeobox protein SIX3	down	dre-miR-204-5p_L+1	down
GS_013459	SIX3	Homeobox protein SIX3	down	dre-miR-204-5p_R+2	down
GS_013459	SIX3	Homeobox protein SIX3	down	PC-5p-46961_19	down

GS_013586	RASEF	Ras and EF-hand domain-containing protein	down	PC-5p-43190_23	down
GS_013618	PRDM1	PR domain zinc finger protein 1	down	dre-miR-204-5p_L+1	down
GS_013618	PRDM1	PR domain zinc finger protein 1	down	dre-miR-204-5p_R+2	down
GS_013701	RASFA	Ras association domain-containing protein 10	down	oan-miR-16b-5p_1ss22GA	down
GS_013701	RASFA	Ras association domain-containing protein 10	down	ola-miR-16	down
GS_013701	RASFA	Ras association domain-containing protein 10	down	tni-miR-15a_R-1	down
GS_013701	RASFA	Ras association domain-containing protein 10	down	tni-miR-16_R-1	down
GS_014384	RETST	Putative all-trans-retinol 13,14-reductase	down	ola-miR-205_L-1R+1_1ss20TA	down
GS_014478	TBX19	T-box transcription factor TBX19	down	dre-miR-20a-3p_2ss7GA11GA	down
GS_014490	XDH	Xanthine dehydrogenase/oxidase	down	ola-miR-205_L-1R+1_1ss20TA	down
GS_014502	CDK15	Cyclin-dependent kinase 15	down	pma-miR-1c-3p_1ss2GT	down
GS_014594	FCAMR	High affinity immunoglobulin alpha and immunoglobulin mu Fc	down	dre-miR-203b-3p_L-1R+2_1ss11CT	down
GS_014684	GTR5	Solute carrier family 2, facilitated glucose transporter member 5	down	dre-miR-204-5p_L+1	down
GS_014684	GTR5	Solute carrier family 2, facilitated glucose transporter member 5	down	dre-miR-204-5p_R+2	down
GS_014685	GTR5	Solute carrier family 2, facilitated glucose transporter member 5	down	dre-miR-9-3p_R+1	down
GS_014685	GTR5	Solute carrier family 2, facilitated glucose transporter member 5	down	pma-miR-1c-3p_1ss2GT	down
GS_014685	GTR5	Solute carrier family 2, facilitated glucose transporter member 5	down	tni-miR-15a_R-1	down
GS_015091	PCDH10	Protocadherin-10	down	dre-miR-26a-5p_R-1_1ss21CT	down
GS_015091	PCDH10	Protocadherin-10	down	hhi-miR-26_R+1	down
GS_015091	PCDH10	Protocadherin-10	down	ola-miR-135b_R+3	down
GS_015091	PCDH10	Protocadherin-10	down	tni-miR-135b_R+1	down
GS_015243	VTCN1	V-set domain-containing T-cell activation inhibitor 1	down	ola-miR-205_L-1R+1_1ss20TA	down
GS_015726	RFT2	Riboflavin transporter 2	down	dre-miR-26a-5p_R-1_1ss21CT	down
GS_015726	RFT2	Riboflavin transporter 2	down	hhi-miR-26_R+1	down
GS_016285	S10I	Ictacalcin	down	PC-5p-26963_57	down
GS_016413	C1ORF10(	Uncharacterized protein C1orf106	down	ola-miR-135b_R+3	down
GS_016413	C1ORF10(	Uncharacterized protein C1orf106	down	tni-miR-135b_R+1	down
GS_016685	MSMB	Beta-microseminoprotein	down	dre-miR-203b-3p_L-1R+2_1ss11CT	down
GS_016685	MSMB	Beta-microseminoprotein	down	dre-miR-9-3p_R+1	down
GS_016931	TAC2N	Tandem C2 domains nuclear protein	down	dre-miR-20a-3p_2ss7GA11GA	down
GS_017984	LIX1	Protein limb expression 1	down	dre-miR-204-5p_L+1	down
GS_017984	LIX1	Protein limb expression 1	down	dre-miR-204-5p_R+2	down
GS_017985	CCD42	Coiled-coil domain-containing protein 42A	down	PC-5p-59593_11	down
GS_018226	ES1	ES1 protein homolog, mitochondrial	down	dre-miR-20a-3p_2ss7GA11GA	down
GS_018226	ES1	ES1 protein homolog, mitochondrial	down	ola-miR-205_L-1R+1_1ss20TA	down
GS_018934	RGR	RPE-retinal G protein-coupled receptor	down	ola-miR-99_R-1	down
GS_019376	HS3S6	Heparan sulfate glucosamine 3-O-sulfotransferase 6	down	PC-5p-46961_19	down

GS_019504	TRPC2	Short transient receptor potential channel 2	down	oan-miR-16b-5p_1ss22GA	down
GS_019504	TRPC2	Short transient receptor potential channel 2	down	ola-miR-16	down
GS_019504	TRPC2	Short transient receptor potential channel 2	down	tni-miR-15a_R-1	down
GS_019504	TRPC2	Short transient receptor potential channel 2	down	tni-miR-16_R-1	down
GS_019678	MPDZ	Multiple PDZ domain protein	down	oan-miR-16b-5p_1ss22GA	down
GS_019678	MPDZ	Multiple PDZ domain protein	down	ola-miR-135b_R+3	down
GS_019678	MPDZ	Multiple PDZ domain protein	down	ola-miR-16	down
GS_019678	MPDZ	Multiple PDZ domain protein	down	tni-miR-135b_R+1	down
GS_019678	MPDZ	Multiple PDZ domain protein	down	tni-miR-15a_R-1	down
GS_019678	MPDZ	Multiple PDZ domain protein	down	tni-miR-16_R-1	down
GS_020150	KINH	Kinesin-1 heavy chain	down	dre-miR-204-5p_L+1	down
GS_020150	KINH	Kinesin-1 heavy chain	down	dre-miR-204-5p_R+2	down
GS_020150	KINH	Kinesin-1 heavy chain	down	dre-miR-20a-3p_2ss7GA11GA	down
GS_020150	KINH	Kinesin-1 heavy chain	down	ola-miR-205_L-1R+1_1ss20TA	down
GS_020430	DES	Desmin	down	dre-miR-202-5p_R-1	down
GS_020470	TT39B	Tetratricopeptide repeat protein 39B	down	ola-miR-205_L-1R+1_1ss20TA	down
GS_020687	TMEM130	Transmembrane protein 130	down	ola-miR-205_L-1R+1_1ss20TA	down
GS_020906	BACE1	Beta-secretase 1	down	dre-miR-204-5p_L+1	down
GS_020906	BACE1	Beta-secretase 1	down	dre-miR-204-5p_R+2	down
GS_020975	TYR	Tyrosinase	down	pma-miR-1c-3p_1ss2GT	down
GS_021002	PNPH	Purine nucleoside phosphorylase	down	dre-miR-204-5p_R+2	down
GS_021002	PNPH	Purine nucleoside phosphorylase	down	ola-miR-135b_R+3	down
GS_021037	IL17REL	Putative interleukin-17 receptor E-like	down	dre-miR-204-5p_L+1	down
GS_021046	WNT7B	Protein Wnt-7b	down	ola-miR-205_L-1R+1_1ss20TA	down
GS_021169	NSG2	Neuron-specific protein family member 2	down	dre-miR-20a-3p_2ss7GA11GA	down
GS_021169	NSG2	Neuron-specific protein family member 2	down	dre-miR-26a-5p_R-1_1ss21CT	down
GS_021169	NSG2	Neuron-specific protein family member 2	down	hhi-miR-26_R+1	down
GS_000465	CO1A2	Collagen alpha-2(I) chain (Fragment)	up	hsa-miR-27a-3p_R+1	up
GS_001591	NKX11	transcription factor-related protein 1	up	pol-miR-199a-5p_R+1	up
GS_002131	MSTN1	Musculoskeletal embryonic nuclear protein 1	up	pol-miR-199a-5p_R+1	up
GS_002994	CRP	C-reactive protein	up	hsa-miR-27a-3p_R+1	up
GS_002994	CRP	C-reactive protein	up	mmu-miR-143-5p_R+2	up
GS_006975	GLRK	Probable glutamate receptor	up	pol-miR-199a-5p_R+1	up
GS_007481	KCC1G	Calcium/calmodulin-dependent protein kinase type 1G	up	oan-miR-139-3p_R-1_1ss8AC	up
GS_009938	DHB14	17-beta-hydroxysteroid dehydrogenase 14	up	mmu-miR-143-5p_R+2	up
GS_010525	FABPI	Fatty acid-binding protein, intestinal	up	pol-miR-199a-5p_R+1	up
GS_011788	-	-	up	hsa-miR-205-5p_R+2	up

GS_011788	-	-	up	tni-miR-205	up
GS_011843	GNS	N-acetylglucosamine-6-sulfatase	up	hsa-miR-27a-3p_R+1	up
GS_013015	PE2R3	Prostaglandin E2 receptor EP3 subtype	up	pol-miR-199a-5p_R+1	up
GS_014186	MSLNL	Mesothelin-like protein	up	mmu-miR-143-5p_R+2	up
GS_018185	HAVR1	Hepatitis A virus cellular receptor 1 homolog	up	hsa-miR-205-5p_R+2	up
GS_018185	HAVR1	Hepatitis A virus cellular receptor 1 homolog	up	tni-miR-205	up
GS_018739	EPD	Ependymin	up	ola-miR-30c_1ss21AG	up
GS_019550	CXA3	Gap junction alpha-3 protein	up	mmu-miR-143-5p_R+2	up
GS_019550	CXA3	Gap junction alpha-3 protein	up	pol-miR-199a-5p_R+1	up
